# Birth order, sibship size, and risk of atopic dermatitis, food allergy, and atopy: A systematic review and meta‐analysis

**DOI:** 10.1002/clt2.12270

**Published:** 2023-06-17

**Authors:** Daniil Lisik, Saliha Selin Özuygur Ermis, Athina Ioannidou, Gregorio Paolo Milani, Sungkutu Nyassi, Giulia Carla Immacolata Spolidoro, Hannu Kankaanranta, Emma Goksör, Göran Wennergren, Bright Ibeabughichi Nwaru

**Affiliations:** ^1^ Krefting Research Centre Department of Internal Medicine and Clinical Nutrition Institute of Medicine University of Gothenburg Gothenburg Sweden; ^2^ Department of Clinical Science and Community Health University of Milan Milan Italy; ^3^ Pediatric Unit Fondazione IRCCS Ca’ Granda Ospedale Maggiore Policlinico Milan Italy; ^4^ Tampere University Respiratory Research Group Faculty of Medicine and Health Technology Tampere University Tampere Finland; ^5^ Department of Respiratory Medicine Seinäjoki Central Hospital Seinäjoki Finland; ^6^ Department of Pediatrics Sahlgrenska Academy at University of Gothenburg Gothenburg Sweden; ^7^ Wallenberg Centre for Molecular and Translational Medicine University of Gothenburg Gothenburg Sweden

**Keywords:** allergic sensitization, atopic dermatitis, atopy, food allergy, systematic review

## Abstract

**Background:**

Atopic dermatitis and food allergy are two frequently concomitant manifestations of the presence of atopy. A substantial number of studies have been published on the association of birth order and sibship size (number of siblings) with atopic dermatitis, food allergy, and atopy. The present work is the first systematic synthesis of the existing literature on this topic.

**Methods:**

Fifteen databases were searched. Screening, data extraction, and quality assessment were performed by independent pairs. Comparable numerical data were statistically synthesized using random‐effects robust variance estimation.

**Results:**

In total, 114 studies were included out of 8819 papers obtained from database searches. Birth order ≥2 versus 1 was associated with lower risk of ever atopic dermatitis (pooled risk ratio [RR] 0.91, 95% CI 0.84–0.98), current food allergy (RR 0.77, 95% CI 0.66–0.90), and positive skin prick test (SPT) to common aeroallergens (RR 0.86, 95% CI 0.77–0.97). Sibship size ≥2 versus 1 was associated with decreased risk of current atopic dermatitis (RR 0.90, 95% CI 0.83–0.98), ever atopic dermatitis (RR 0.92, 95% CI 0.86–0.97), and positive SPT to common aeroallergens (RR 0.88, 95% CI 0.83–0.92). No putative associations were seen regarding atopy assessed through allergen‐specific immunoglobulin E with common allergens.

**Conclusion:**

The presence of siblings and being second‐born or later may decrease the lifetime risk of atopic dermatitis and food allergy, albeit marginally. Similar association was seen with SPT sensitization. However, significant protection was not found for IgE sensitization.

## 1 INTRODUCTION

Atopic dermatitis and food allergy are two common atopic diseases.^1^ Atopic dermatitis, the most common chronic relapsing inflammatory skin disease, is particularly often seen in children, with reports indicating that up to a third are affected in Northern Europe. Studies in adults estimate that it affects, on average, close to 10% of the general population in the Europe and United States.^2^, ^3^, ^4^ An increase in atopic dermatitis has been noted in several regions of the world in recent decades, including Africa, eastern Asia, and parts of Europe.^5^ Similarly, the most widely recognized form of food allergy,^6^ immunoglobulin E (IgE)‐mediated food allergy, affects 1%–10% in the general population, with higher prevalence seen in Western than non‐Western countries and in young children.^7^, ^8^, ^9^, ^10^ Also food allergy has increased in prevalence in recent decades, particularly in developed countries,^9^ but a substantial heterogeneity remains in distribution and recent trends between continents and countries.^7^, ^11^, ^12^ Atopic dermatitis and food allergy are associated^13^ and commonly co‐exist in the same individual,^14^ not seldom as components in the “atopic march,” which typically begins with atopic dermatitis, subsequently progressing into food allergy and other atopic diseases.^15^, ^16^, ^17^ Both conditions, especially in early life, are common manifestations of atopy, which is an immunological predisposition characterized by exaggerated IgE production against otherwise commonly innocuous environmental allergenic molecules,^18^ but such sensitization is asymptomatic in some individuals, meaning that no symptoms are seen upon exposure to the allergen.^19^, ^20^

The underlying reasons for the heterogeneous triggers, clinical presentations, and trajectories of atopic dermatitis, food allergy, and atopy, are not yet fully elucidated, but are thought to be constituted of a complex set of interrelated (epi)genetic, immunological, and environmental factors.^21^, ^22^, ^23^ As a substantial increase in the prevalence of atopic diseases has been reported in developed as well as rapidly developing and industrializing/urbanizing countries,^8^, ^24^ the role of changes in lifestyle and environment in these regions as contributors to the observed increase have been of interest in research. Strachan popularized the “hygiene hypothesis,” which suggested that the presence of older siblings at home may confer protection against the development of allergy.^25^ While initially highlighting the association with allergic rhinitis, a substantial body of research has subsequently been published on the role of sibship composition with related outcomes. We undertook the present systematic review and meta‐analysis to synthesize the existing literature on the association between birth order and sibship size (number of siblings) and risk of atopic dermatitis, food allergy, and atopy. To the best of our knowledge, this is the first systematic review of this topic.

## 2 METHODS

This work was performed following a prospectively registered (International prospective register of systematic reviews [PROSPERO]; CRD42020207905) and published^26^ protocol, based on the Preferred Reporting Items for Systematic Review and Meta‐Analysis Protocols (PRISMA‐P) guidelines.^27^ Reporting of the present work was based on the Preferred Reporting Items for Systematic Reviews and Meta‐Analyses (PRISMA)^28^ checklist (Supporting Information S1: Table E1) and the Meta‐analysis of Observational Studies in Epidemiology (MOOSE)^29^ reporting guidelines (Supporting Information S1: Table E2).

### 2.1 Inclusion and exclusion criteria

Studies—regardless of sample size or medical/sociodemographic background of subjects—fulfilling the following criteria were eligible for inclusion:

1. *Study design*: observational studies (cross‐sectional, case‐control, and cohort studies).
2. *Exposure*: sibship composition, that is, birth order and/or sibship size (number of siblings).
3. *Outcome*: any of (a) atopic dermatitis (self‐reported or clinically assessed/diagnosed), (b) food allergy (self‐reported or clinically assessed/diagnosed to any food[s]), (c) atopy (positive skin prick test [SPT] or allergen‐specific immunoglobulin E [sIgE] to any allergen; chosen as these are two of the most common assessment methods in the published literature^30^, ^31^, ^32^).

### 2.2 Data sources and search strategy

We searched the following databases without restriction on publication year: AMED, CABI, CINAHL, Embase, Google Scholar, OAIster, Open Access Theses and Dissertations, Open Grey, ProQuest Dissertations & Theses Global, PsycINFO, PubMed, SciELO, Scopus, Web of Science, and WHO Global Index Medicus were searched. An initial search was performed on September 30, 2020 and a follow‐up search on October 20, 2021. The first 300 results from Google Scholar were screened and added to the records obtained from other databases.^33^ Non‐English articles were translated with Google Translate.^34^ The database searches were complemented by hand‐searching of reference lists in the included studies. Queries used to perform the searches are presented in Supporting Information S1: Table E3A–I.

### 2.3 Study selection and data extraction

EndNote X9 (Clarivate Analytics, 2020) was used to host‐retrieved records and perform de‐duplication, following a semi‐automated method proposed by Bramer et al.^35^ Pairs of reviewers independently screened records following a two‐step approach, based on (1) title and abstract, and (2) full‐text of potentially relevant articles. Similarly, pairs of reviewers independently extracted data from the included studies. The following data were extracted from each article: main author, year of publication, study design, the source of subjects (e.g., among male conscripts undergoing medical examination at recruitment offices or from the general pediatric population), country, number, and age of subjects, definition/assessment method of exposure and outcome, and point estimates with 95% confidence interval (95% CI) for relevant exposure‐outcome pairs. After each step during screening and data extraction, the decisions were unblinded and compared for agreement. A third reviewer (BIN) arbitrated when needed.

### 2.4 Quality assessment

Risk of bias in the included studies was assessed using the Effective Public Health Practice Project^36^ tool, with slight modifications used in a work by Smith et al.^37^ to better fit the investigated data. Based on the rating of “weak,” “moderate,” or “strong” in six domains (study design, selection bias, confounding, blinding, data collection, and withdrawals/dropouts), an overall rating was given based on the number of “weak” domains: “weak” if more than one, “moderate” if one, and “strong” if none of the domains had given a “weak” rating. The risk of bias assessment was performed independently in pairs of reviewers. After completion, differences were discussed, and a third reviewer (BIN) arbitrated when needed.

### 2.5 Data synthesis and statistical analysis

Relevant data from the included studies are summarized and presented in tables of characteristics for each outcome (atopic dermatitis, food allergy, and atopy). The overall findings were synthesized narratively. Comparable numerical data—based on similarity in exposure, outcome, assessment methods and subject characteristics—were also synthesized statistically using meta‐analysis with random‐effects robust variance estimation (RVE).^38^ RVE enables the inclusion of data with various structures of dependency in the same meta‐analysis model, for example, different sibship sizes or birth orders compared to the same reference group (single children and first‐born, respectively), which constituted the majority of dependent estimates in our data.

The meta‐analyses were performed using the correlated effects model, with small sample‐correction (to increase accuracy)^39^ and the default value of rho (defining the intra‐study effect size correlation; 0.8), and implemented in R statistical software (version 4.2.0; R Core Team 2022) using the *robumeta* R package.^38^ Separate meta‐analyses were performed for each exposure type—(a) birth order, (b) sibship size—in relation to each outcome—(a) current (in last year) atopic dermatitis, (b) ever atopic dermatitis, (c) any current (in last year) food allergy, (d) any food allergy ever, (e) atopy (positive sIgE), (f) atopy (positive SPT)—for which comparable data were available from ≥2 studies.^40^ For birth order, being first‐born constituted the reference group, and for sibship size the reference group was single children. The reciprocal of the point estimate as well as the lower and upper bounds of the 95% CI was calculated in cases where the reference group was of higher cardinality, for example, birth order <3 versus ≥3. Forest plots from the meta‐analyses were produced using the *forestploter* R package.^41^

Subgroup analyses were performed where comparable data were available from ≥4 studies in ≥2 subgroups,^42^ to investigate sources of heterogeneity in findings, based on (a) study design; (b) exposure cardinality (e.g., sibship size 4); (c) overall rating; (d) year(s) of data collection, divided into <2000 and ≥2000; (e) World Bank classification^43^ of the study country in the year of publication into “high income,” “upper‐middle income,” “lower‐middle income,” and “low income” economy; (f) subject age, divided into children (<18 years) and adults. Additionally, sensitivity analyses were performed to assess the robustness of our pooled estimates by re‐running the meta‐analysis only on studies with (a) a “moderate” or a “high” overall rating and (b) physician/clinical outcome assessment (or report thereof). Sensitivity analysis was also performed based on the rho value, by re‐running the meta‐analysis using rho values ranging from 0 to 1, with 0.2 increments, using the *sensitivity()* function from the *robumeta* R package.^39^ Finally, publication bias was assessed in exposure‐outcome pairs with 10 studies^44^ using the *metafor* R package^45^ and a two‐fold approach: (1) visual assessment of funnel plots for signs of asymmetry; (2) statistical assessment using Egger's regression test^46^ and Begg and Mazumdar rank correlation, considering corresponding *p*‐values <0.05 as statistically significant.

Risk ratio (RR) was used as the measure of effect due to ease of interpretation.^47^, ^48^, ^49^ Prevalence ratio data were used without conversion as these are calculated identically with RR.^47^ Odds ratio and hazard ratio data were converted to estimates of RR in studies where the outcome prevalence was ≥15% (at the end of follow‐up) using the following formulae:

-
$$RR\approx \sqrt{OR}$$
-
$$RR\approx \;\frac{1-0.5^{\sqrt{HR}}}{1-0.5^{\sqrt{\frac{1}{HR}}}}$$

Inter‐study heterogeneity was assessed with the *I*‐squared (*I* ^2^) statistic,^50^ while inter‐study variance was estimated with Tau‐squared (*τ* ^2^).^51^ Meta‐analysis outputs with Satterwhite degrees of freedom (df) <4 were considered unreliable.^39^ All R scripts and data used to perform the meta‐analyses are freely available at the Open Science Framework (https://osf.io/fp3rw/).

## 3 RESULTS

**FIGURE 1:**
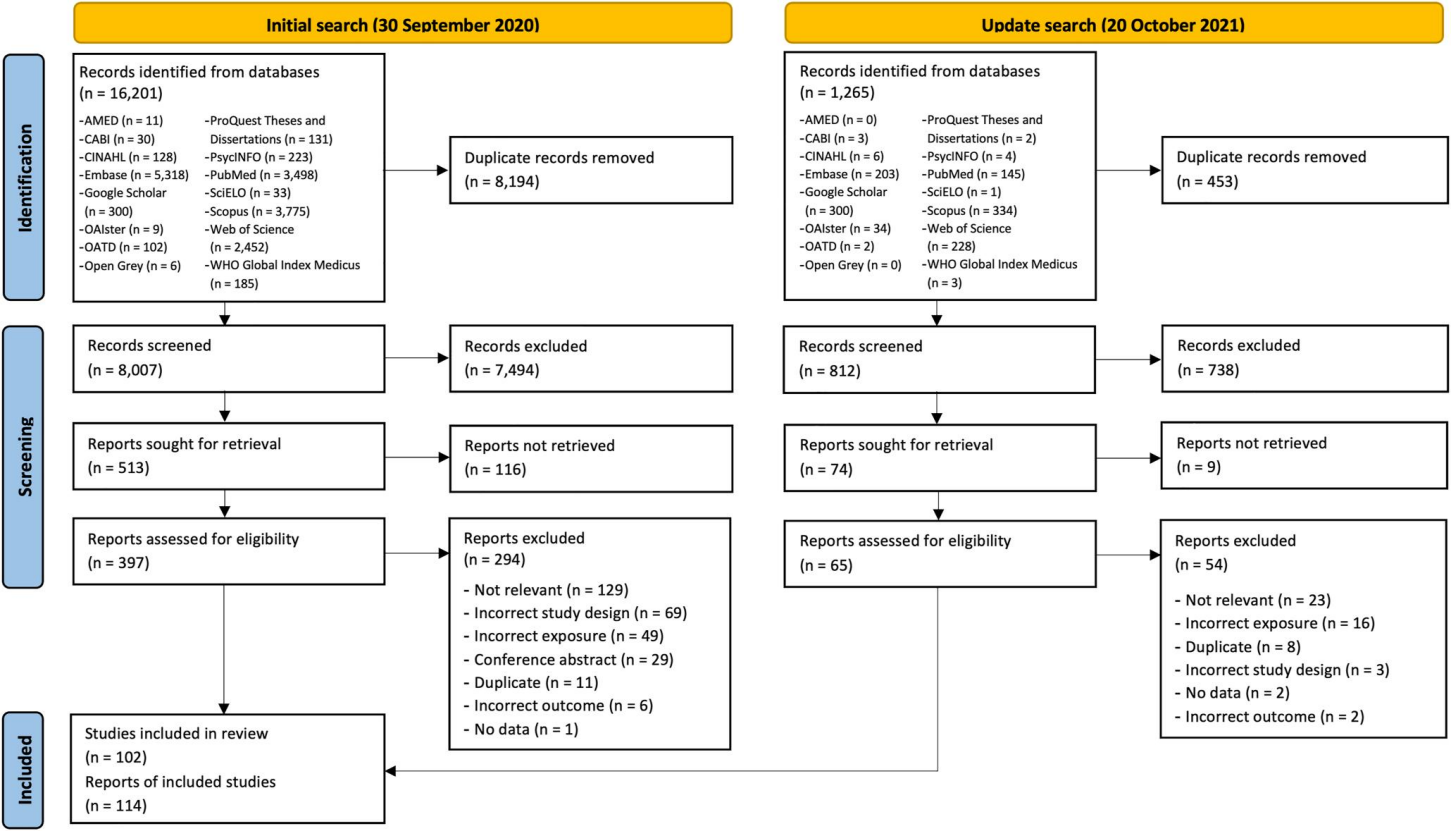
Preferred Reporting Items for Systematic Reviews and Meta‐Analyses (PRISMA) flow diagram.

The database searches yielded 17,466 records. Of these, 114 reports based on 102 studies met the full inclusion criteria (Figure 1).

### 3.1 Study characteristics

**FIGURE 2:**
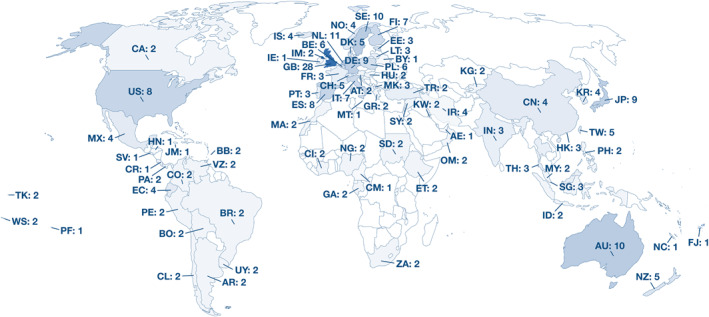
A map of the countries of participants in the included studies. The two letter code indicates the country name and the number indicates how many reports there are from said country. AE, United Arab Emirates; AR, Argentina; AT, Austria; AU, Australia; BB, Barbados; BE, Belgium; BO, Bolivia (Plurinational State of); BR, Brazil; BY, Belarus; CA, Canada; CH, Switzerland; CI, Côte d'Ivoire; CL, Chile; CM, Cameroon; CN, China; CO, Colombia; DE, Germany; DK, Denmark; EC, Ecuador; EE, Estonia; ES, Spain; ET, Ethiopia; FI, Finland; FJ, Fiji; FR, France; GA, Gabon; GB, United Kingdom of Great Britain and Northern Ireland; GR, Greece; HK, Hong Kong; HN, Honduras; HU, Hungary; ID, Indonesia; IE, Ireland; IM, Isle of Man; IN, India; IR, Iran (Islamic Republic of); IS, Iceland; IT, Italy; JM, Jamaica; JP, Japan; KG, Kyrgyzstan; KR, Korea, Republic of; KW, Kuwait; LT, Lithuania; MA, Morocco; MK, North Macedonia; MT, Malta; MX, Mexico; MY, Malaysia; NC, New Caledonia; NG, Nigeria; NL, Netherlands; NO, Norway; NZ, New Zealand; OM, Oman; PA, Panama; PE, Peru; PF, French Polynesia; PH, Philippines; PL, Poland; PT, Portugal; SD, Sudan; SE, Sweden; SG, Singapore; SV, El Salvador; SY, Syrian Arab Republic; TH, Thailand; TK, Tokelau; TR, Turkey; TW, Taiwan, Province of China; US, United States of America; UY, Uruguay; VE, Venezuela (Bolivarian Republic of); WS, Samoa; ZA, South Africa.

**FIGURE 3:**
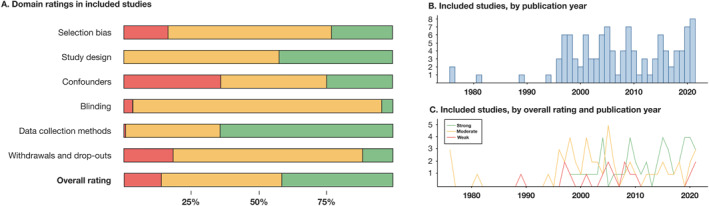
(A) Domain ratings and overall rating of the included studies (red: “weak,” yellow: “moderate,” green: “strong” rating). (B) Number of studies published by the year among the included studies. (C) Overall rating of the included studies by years.

Across the included studies, data from 75 countries and a total of >2 million subjects were available (Figure 2). The most common study design was cross‐sectional (*n* = 55), followed by cohort studies (*n* = 43), case‐control studies (*n* = 11), nested case‐control studies (*n* = 4), and one case‐cohort study. Most studies received a “moderate” (*n* = 51; 45%) or “strong” (*n* = 47; 41%) overall rating, while 16 (14%) were rated “weak” (Figure 3A, Supporting Information S1: Table E4). The vast majority of studies were published after the turn of the millennium. Similarly, the assessed quality increased substantially in studies published in recent years (Figure 3B,C). See Supporting Information S1: Table E5A–C for detailed characteristics of the included studies.

### 3.2 Current atopic dermatitis

**FIGURE 4:**
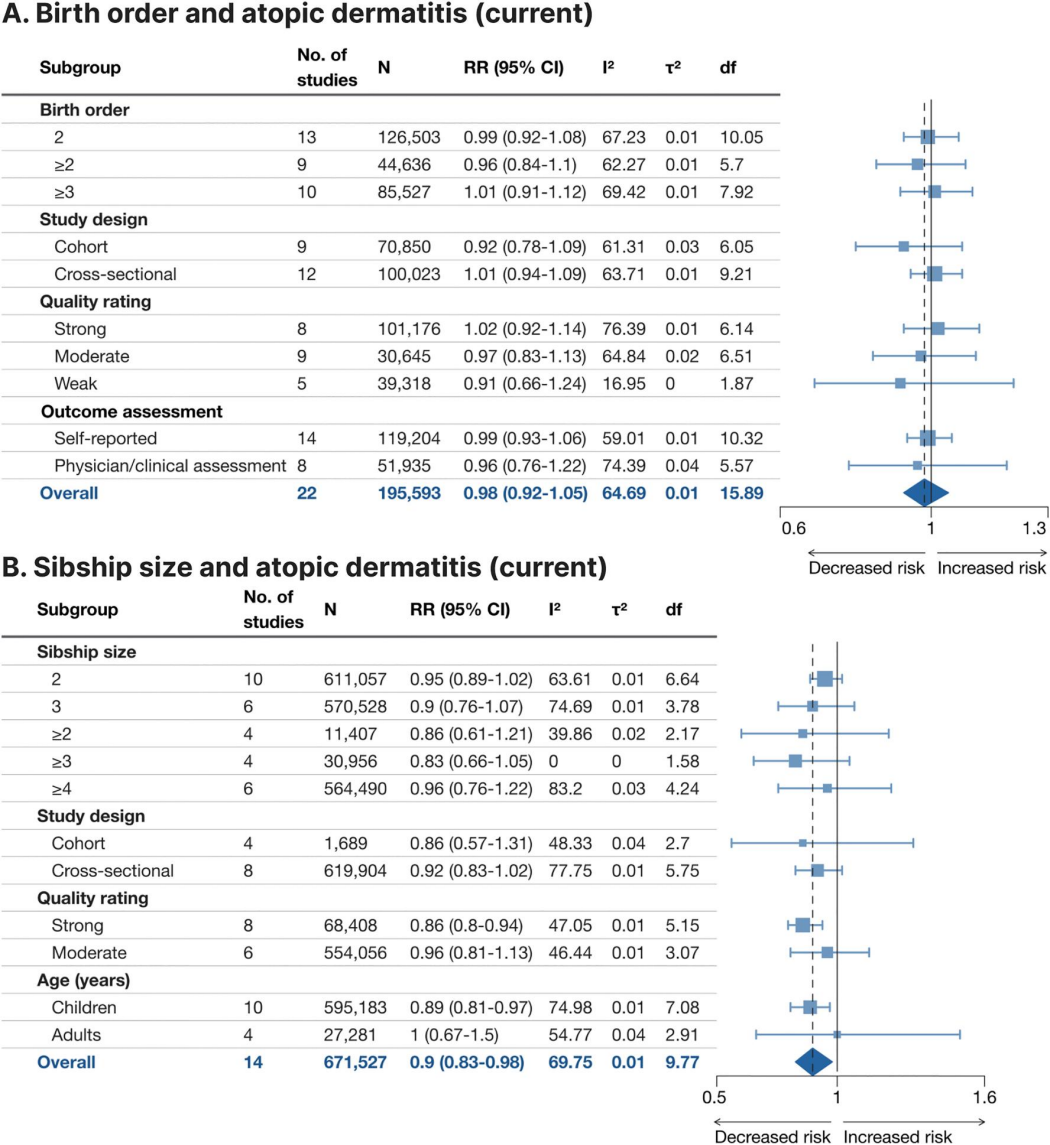
Forest plot for birth order ≥2 versus 1 (A) and sibship size ≥2 versus 1 (B) in relation to current (in last year) atopic dermatitis. df, Satterwhite degrees of freedom; *I* ^2^, *I*‐squared; *N*, number of subjects (if not available, the number of subjects for the most similar exposure‐outcome pair or for the whole study is stated); RR (95% CI), risk ratio (95% confidence interval); *τ* ^2^, Tau‐squared.

Current atopic dermatitis was assessed with meta‐analysis in 22 studies for birth order and 14 studies for sibship size (Figure 4, Supporting Information S1: Figure E1A,B). The pooled effect size indicated that sibship size ≥2 versus 1 was associated with a 10% lower risk (RR 0.90, 95% CI 0.83–0.98). The association was particularly clear in children with at least one sibling (RR 0.89, 95% CI 0.81–0.97). The pooled point estimate decreased relatively consistently with increased sibship size, but no single sibship size was associated with a statistically significant effect. The association with birth order ≥2 versus 1 was non‐significant (RR 0.98, 95% CI 0.92–1.05), and no clear association was found in the subgroup analyses. Heterogeneity was overall moderate for both birth order (*I* ^2^ = 64.7%, *τ* ^2^ = 0.01) and sibship size (*I* ^2^ = 69.8%, *τ* ^2^ = 0.01).

### 3.3 Ever atopic dermatitis

**FIGURE 5:**
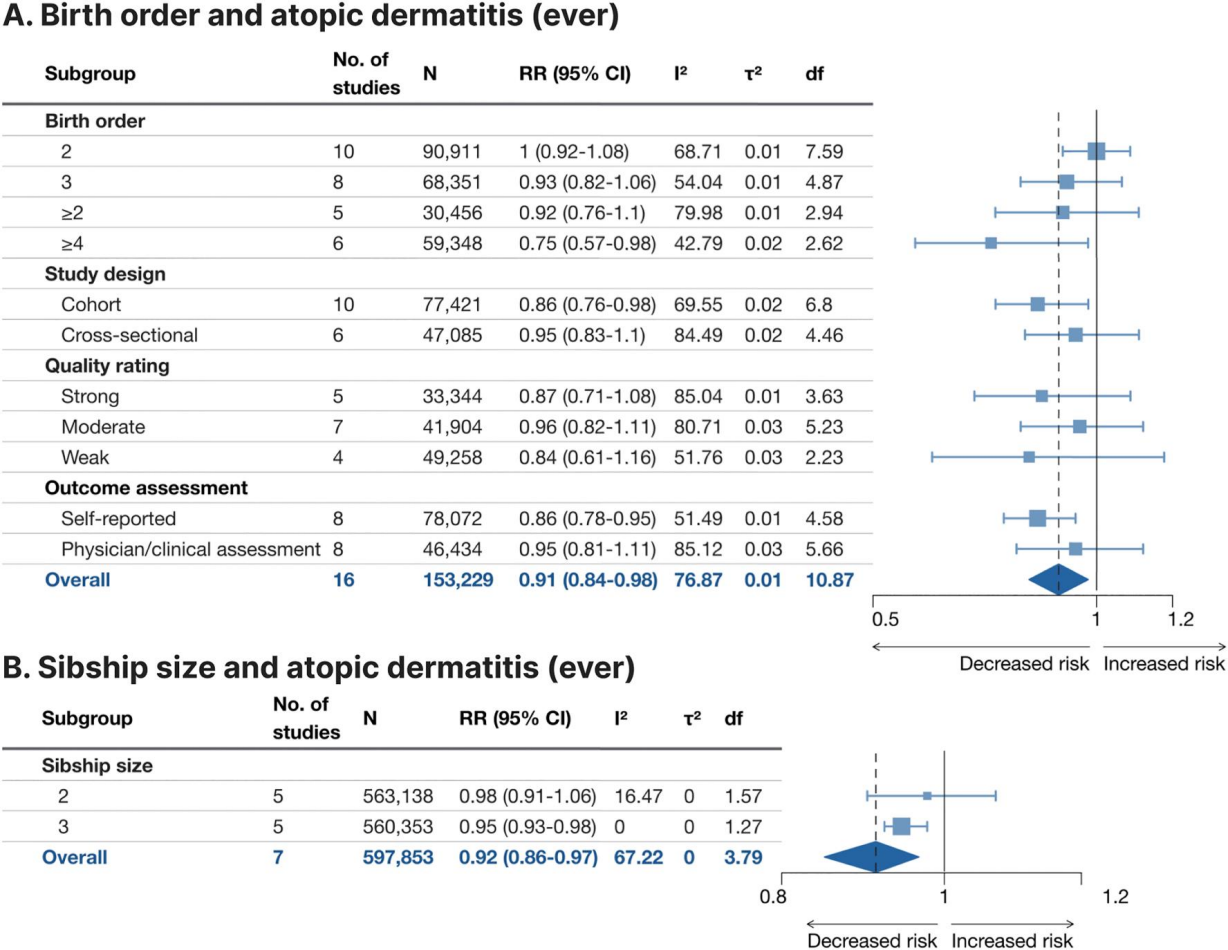
Forest plot for birth order ≥2 versus 1 (A) and sibship size ≥2 versus 1 (B) in relation to ever atopic dermatitis. df, Satterwhite degrees of freedom; *I* ^2^, I‐squared; *N*, number of subjects (if not available, the number of subjects for the most similar exposure‐outcome pair or for the whole study is stated); RR (95% CI), risk ratio (95% confidence interval); *τ* ^2^, Tau‐squared.

Ever atopic dermatitis was assessed with meta‐analysis in 16 studies for birth order and seven studies for sibship size (Figure 5, Supporting Information S1: Figure E2A,B). The overall pooled effect size indicated a 9% lower risk with birth order ≥2 versus 1 (RR 0.91, 95% CI 0.84–0.98) and an 8% lower risk with sibship size ≥2 versus 1 (RR 0.92, 95% CI 0.86–0.97). Point estimates decreased relatively consistently with increased birth order and sibship size, respectively. For birth order, the association with self‐reported atopic dermatitis was stronger (RR 0.86, 95% 0.78–0.95) than in studies with physician/clinical assessment of the outcome (RR 0.95, 95% CI 0.81–1.11). Heterogeneity was slightly high for birth order (*I* ^2^ = 77.9%, *τ* ^2^ = 0.01) and moderate for sibship size (*I* ^2^ = 67.2%, *τ* ^2^ = 0).

### 3.4 Current food allergy

**FIGURE 6:**
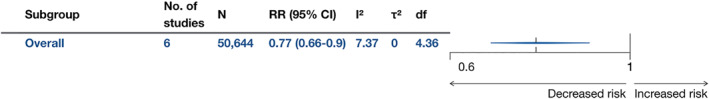
Forest plot for birth order ≥2 versus 1 in relation to any current (in last year) food allergy. df, Satterwhite degrees of freedom; *I* ^2^, I‐squared; *N*, number of subjects (if not available, the number of subjects for the most similar exposure‐outcome pair or for the whole study is stated); RR (95% CI), risk ratio (95% confidence interval); *τ* ^2^, Tau‐squared.

The current food allergy was assessed with meta‐analysis in six studies for birth order (Figure 6, Supporting Information S1: Figure E3). The pooled effect size indicated that having at least one older sibling was associated with a 23% lower risk of the outcome (RR 0.77, 95% CI 0.66–0.90). The number of studies was inadequate to perform subgroup analysis. All included studies were performed in children from infancy up to the age of 15 years. Two studies investigated the self‐reported food allergy, while the four other studies used either physician‐diagnosed food allergy as the outcome only or either physician‐diagnosed or self‐reported food allergy. Overall heterogeneity was low (*I* ^2^ = 7.4%, *τ* ^2^ = 0).

### 3.5 Ever food allergy

There were too few comparable studies to perform meta‐analysis on this outcome or to make a clear assessment narratively, as the included studies varied substantially in exposures investigated and subject characteristics (Supporting Information S1: Table E5B). In a study on cow's milk allergy specifically, a reduced risk of the outcome was seen in subjects with ≥4 older siblings.^52^ On the other hand, a register‐based study on any food allergy diagnosis found no association with birth order ≥2 versus 1.^53^ Similarly, Of the two population‐based studies on sibship size and any food allergy, one indicated a lower risk of the outcome in subjects with ≥6 versus <6 siblings,^54^ while the other reported no significant association.^55^

### 3.6 Atopy

**FIGURE 7:**
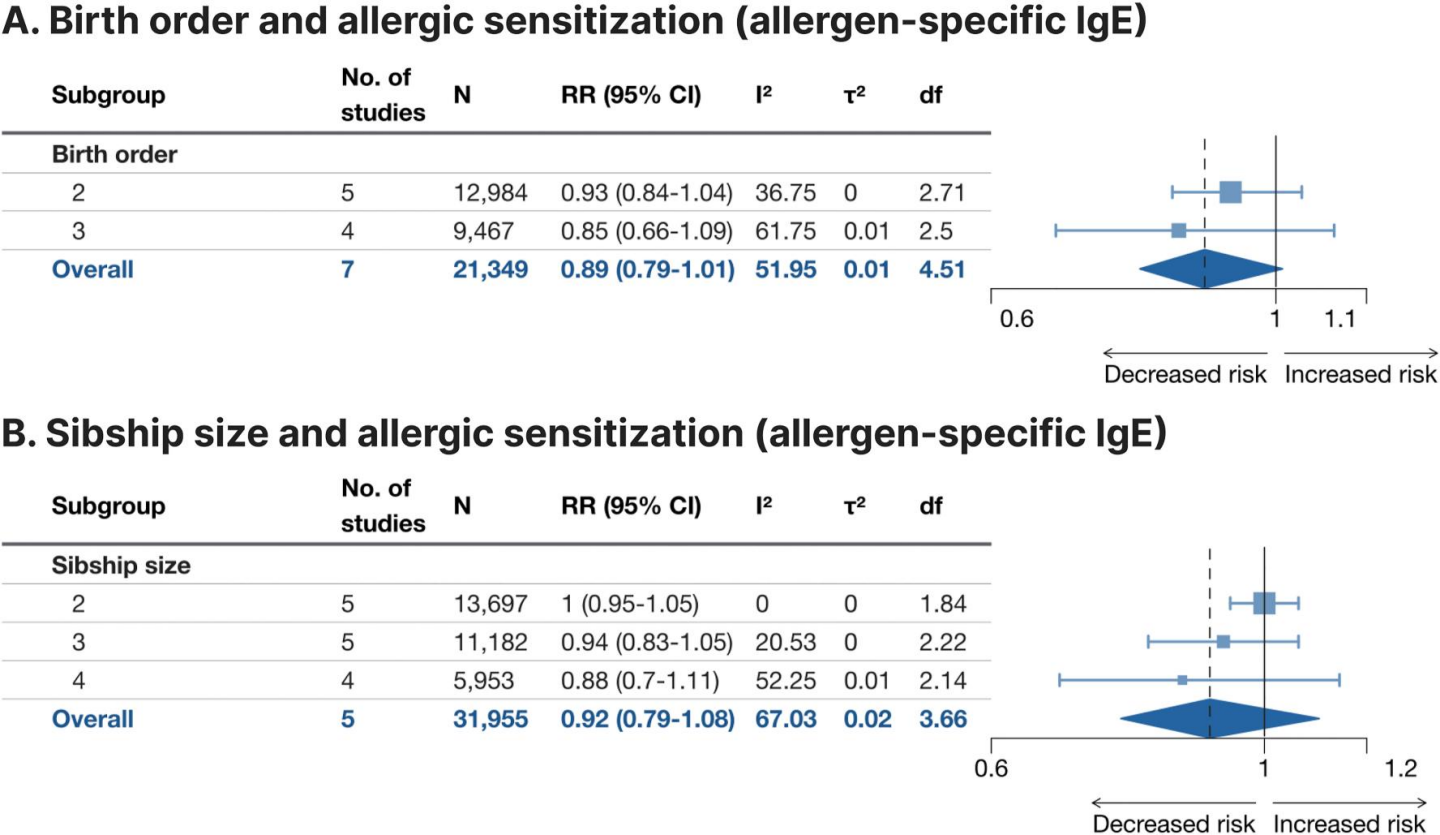
Forest plot for birth order ≥2 versus 1 (A) and sibship size ≥2 versus 1 (B) in relation to allergic sensitization assessed by measurement of allergen‐specific immunoglobulin E (sIgE) levels to palettes of common food allergens and aeroallergens (A) and common aeroallegergens (B). df, Satterwhite degrees of freedom; *I* ^2^, I‐squared; *N*, number of subjects (if not available, the number of subjects for the most similar exposure‐outcome pair or for the whole study is stated); RR (95% CI), risk ratio (95% confidence interval); *τ* ^2^, Tau‐squared.

**FIGURE 8:**
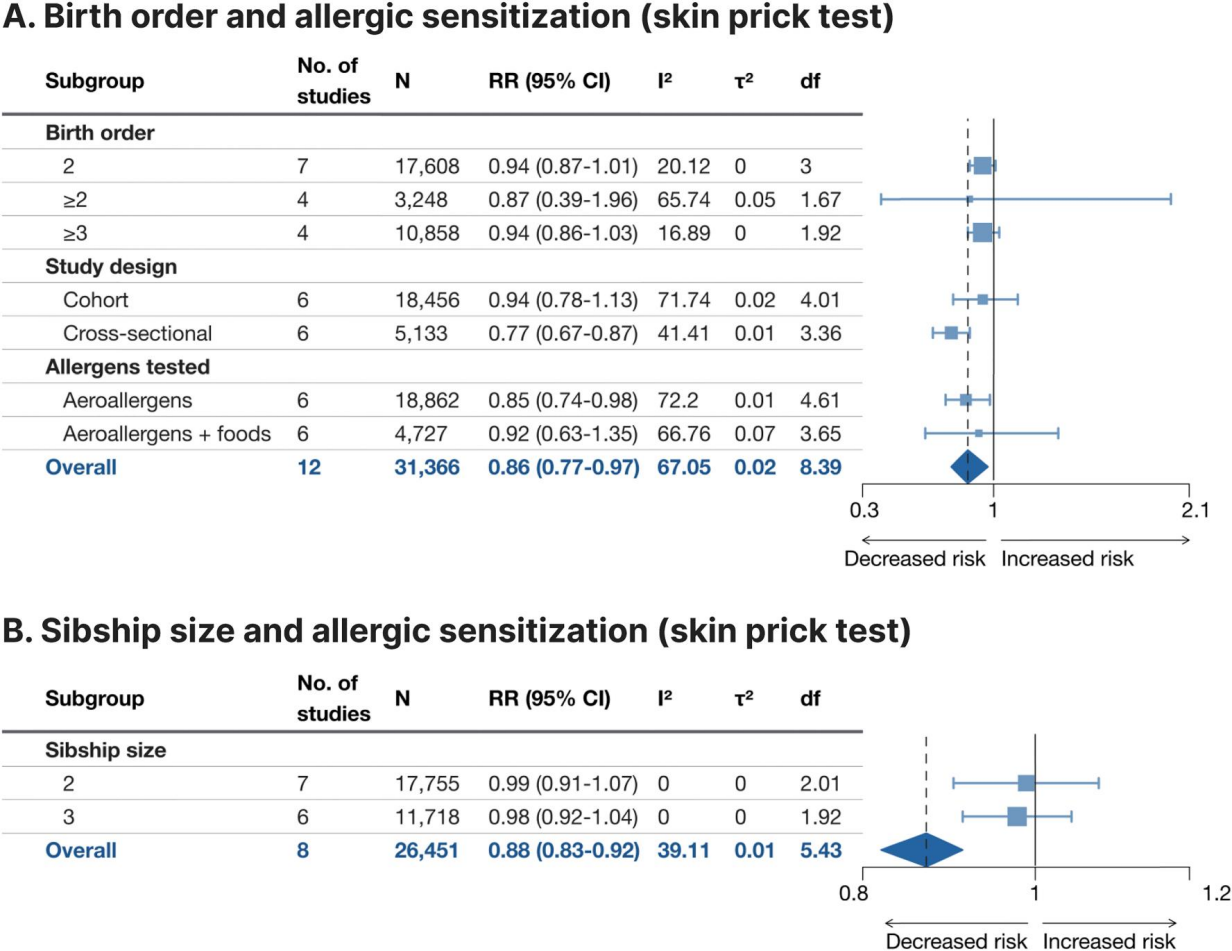
Forest plot for birth order ≥2 versus 1 (A) and sibship size ≥2 versus 1 (B) in relation to allergic sensitization assessed by skin prick tests (SPT) to common food allergens and aeroallergens (A) and common aeroallergens (B). df, Satterwhite degrees of freedom; *I* ^2^, I‐squared; *N*, number of subjects (if not available, the number of subjects for the most similar exposure‐outcome pair or for the whole study is stated); RR (95% CI), risk ratio (95% confidence interval); *τ* ^2^, Tau‐squared.

Atopy as defined through sIgE levels above the traditional^56^, ^57^ threshold of 0.35 kU_A_/L was assessed in seven studies for birth order (using combinations of aeroallergen, some of which included common foods) and five studies for sibship size (using combinations of aeroallergens; Figure 7, Supporting Information S1: Figure E4A,B). The effects of both birth order ≥2 versus 1 and sibship size ≥2 versus 1 were comparable (RR 0.89, 95% CI 0.79–1.01 and RR 0.92, 95% CI 0.79–1.08, respectively), each statistically non‐significant. In subgroup analysis, a trend with decreasing point estimate with increased cardinality of the exposure could be discerned. Heterogeneity was moderate for both birth order (*I* ^2^ = 51.9%, *τ* ^2^ = 0.01) and sibship size (*I* ^2^ = 67.0%, *τ* ^2^ = 0.02). For atopy as defined through positive SPT, the pooled effect size from the 12 studies on birth order (using combinations of aeroallergens, some of which included common foods; Figure 8A, Supporting Information S1: Figure E5A) indicated that birth order ≥2 versus 1 was associated with 14% lower risk of the outcome (RR 0.86, 95% CI 0.77–0.97). In the studies including food allergens, the association was not statistically significant, however. Similarly, the pooled effect size of the eight studies on sibship size (using combinations of aeroallergens; Figure 8B, Supporting Information S1: Figure E5B) indicated that sibship size ≥2 versus 1 was associated with 12% lower risk of the outcome (RR 0.88, 95% CI 0.83–0.92). Heterogeneity was moderate for birth order (*I* ^2^ = 67.1%, *τ* ^2^ = 0.02) and low for sibship size (*I* ^2^ = 39.1%, *τ* ^2^ = 0.01).

### 3.7 Publication bias and sensitivity analysis

The *p*‐value of 0.04 from the Egger's regression test for atopic dermatitis by birth order indicated asymmetry (Supporting Information S1: Figure E6, Table E6). Seven estimates were filled on the right side with the trim‐and‐fill method (Supporting Information S1: Figure E7), but following visual inspection of the corresponding funnel plots, it is unlikely, given the overall concentrated distribution of published and filled results around the top center, that the pooled estimates are biased, although slight publication bias cannot be ruled out. Two estimates were also filled in for allergic sensitization (SPT) by birth order (Supporting Information S1: Figure E7), but the non‐significant *p‐*values from the statistical tests (Supporting Information S1: Table E6) and the weak asymmetry similarly suggest that the results are most likely not biased.

Sensitivity analyses by excluding studies with overall “weak” rating and those with self‐reported outcome assessment did not produce substantially different pooled estimates. For the sensitivity analysis by physician/clinical outcome assessment on current atopic dermatitis by sibship size, the 95% CI became wider, no longer indicating a statistically significant association. However, the point estimate was lower in this study subsample, which only consisted of four studies, thus likely being the result of low statistical power (Supporting Information S1: Table E7). Similarly, a marginally wider 95% CI, most likely due to the same reason, was seen for all atopic dermatitis by birth order, excluding “weak”‐rated studies (Supporting Information S1: Table E7). Sensitivity analyses by rho did not notably affect the effect sizes (Supporting Information S1: Table E8).

## 4 DISCUSSION

### 4.1 Summary of key findings

The present work constitutes a comprehensive synthesis of the global literature on this topic. The available data indicate that having (older) siblings was only marginally associated with lifetime risk of having atopic dermatitis, with a weaker impact on current atopic dermatitis. In contrast, the presence of older siblings was associated with a substantially decreased risk of current food allergy, albeit with only a few studies. In terms of allergic sensitization (older), siblings appeared to be marginally associated with protection against atopy to common aeroallergens assessed with SPT, but the association was not significant for atopy assessed with sIgE positivity. Because of the small number of studies in each exposure‐outcome pair, and the socioeconomic homogeneity across studies, meaningful comparison on the effect of publication year and World Bank economic classification was not possible.

### 4.2 Strengths and limitations

The exhaustive search of 15 databases—with no restriction by time, language, or outcome definitions—identified a broad and comprehensive scope of relevant literature from across the world. Furthermore, the use of RVE enabled us to include correlated estimates in robust meta‐analyses, and consequently to overcome any issue of multi‐collinearity between the effect estimates. However, the included studies were largely heterogeneous in methodology and subject characteristics, thus limiting the number of comparable studies to synthesize with meta‐analysis. Similarly, the socioeconomic homogeneity, with the vast majority of studies conducted in Western countries after the turn of the millennium, did not allow for the assessment of the effect of changes in lifestyle and living conditions in recent decades on the investigated associations. The underlying data, derived from observational studies, which are prone to confounding (particularly older studies with insufficient confounder adjustment), limits causal inference.^58^, ^59^, ^60^ Finally, the inclusion of self‐reported atopic dermatitis and food allergy may limit the clinical validity and precision of the derived estimates.^61^

### 4.3 Comparison of findings to previous studies

To the best of our knowledge, the present work is the first systematic review and meta‐analysis synthesizing the literature on the association between birth order and sibship size with risk of atopic dermatitis, food allergy, and atopy.

### 4.4 Interpretation of findings

Both the presence of siblings and being second‐born or later was associated with a marginally lower risk of atopic dermatitis. A similar strength in the association was seen between the presence of siblings and current atopic dermatitis, but the association was non‐significant in second‐born or later. As atopic dermatitis is more commonly seen in childhood (particularly early childhood) with the majority no longer experiencing symptoms in adulthood,^3^, ^19^ and that the strongest effect was seen in children in regard to current atopic dermatitis, it may be that the effect of the association is limited to certain phenotypes of the disease^62^, ^63^, ^64^ or a specific time‐window.

While the protective effect of birth order above one on the current food allergy was clearer, it was also based on a relatively small set of studies. Thus, while a 23% reduction in the risk of current food allergy was seen, it is difficult to assess the robustness and generalizability of this association.

The association between sibship composition and atopy was relatively similar in terms of exposure, but the association was weaker for both exposure types in relation to sIgE compared with SPT outcomes. Age may be part of the explanation in this case,^65^ similar to in the studies on atopic dermatitis, as substantially more studies using sIgE were on adults, in contrast to studies using SPT, where a clear majority were children. Furthermore, while both birth order ≥2 versus 1 and sibship size ≥2 versus 1 indicate the same, albeit weak and possibly practically insignificant, effect, the slight difference in strength and precision of the association may be due to the differences in sensitivity and specificity of the (arbitrary) cut‐offs in the different assessment methods.^57^

All in all, the findings indicate that for the investigated outcomes, the association with sibship composition is weak, if practically meaningful at all. The causes for this may be, for example, the heterogeneity of allergic sensitization and these atopic diseases or changes in environmental factors that we were unable to account for the meta‐analyses.

### 4.5 Clinical and research implications

Although a protective effect of the presence of (older) siblings was seen across all outcomes, the strength of the association varied substantially between outcomes and age groups. Thus, while our findings partly support the “hygiene hypothesis”—namely that early life cross‐infection between siblings can modulate the immune systems in such a way that the risk of allergy development is reduced^66^—they particularly highlight the complex underlying pathophysiological mechanisms and heterogeneous clinical presentations and trajectories of allergic sensitization and atopic diseases alike.^62^, ^67^ Our synthesis can potentially be used as a stepping‐stone in furthering our understanding of the underlying mechanisms driving allergic sensitization and allergy development, as well as to direct future epidemiological research in terms of environmental factors in relation to the investigated diseases.

## 5 CONCLUSION

Our findings indicate that having siblings and being second‐born or later is associated with a marginal reduced risk of lifetime risk of atopic dermatitis. Likewise, a higher birth order is associated with roughly 20% lower risk of current food allergy. Allergic sensitization defined by SPT to common aeroallergens is marginally rarer in those with siblings or second‐born or later, while the association for sensitization measured using sIgE did not reach statistical significance. Atopic diseases are heterogeneous and multifactorial, so it is likely that sibship composition only plays a marginal role in the risk of these diseases.

## AUTHOR CONTRIBUTIONS

**Daniil Lisik**: Data curation (equal); Formal analysis (lead); Investigation (lead); Methodology (equal); Project administration (lead); Software (lead); Validation (equal); Visualization (lead); Writing – original draft (lead); Writing – review & editing (lead). **Saliha Selin Özuygur Ermis**: Data curation (supporting); Formal analysis (supporting); Investigation (supporting); Methodology (supporting); Project administration (supporting); Validation (equal); Writing – review & editing (equal). **Athina Ioannidou**: Data curation (supporting); Investigation (supporting); Validation (supporting); Writing – review & editing (supporting). **Gregorio Paolo Milani**: Data curation (supporting); Investigation (supporting); Validation (supporting); Writing – review & editing (supporting). **Sungkutu Nyassi**: Data curation (supporting); Investigation (supporting); Validation (supporting); Writing – review & editing (supporting). **Giulia Carla Immacolata Spolidoro**: Data curation (supporting); Investigation (supporting); Validation (supporting); Writing – review & editing (supporting). **Hannu Kankaanranta**: Formal analysis (supporting); Investigation (supporting); Methodology (supporting); Supervision (supporting); Validation (supporting); Writing – review & editing (supporting). **Emma Goksör**: Formal analysis (supporting); Supervision (supporting); Validation (supporting); Writing – review & editing (supporting). **Göran Wennergren**: Formal analysis (supporting); Methodology (supporting); Supervision (supporting); Validation (supporting); Writing – review & editing (supporting). **Bright Ibeabughichi Nwaru**: Conceptualization (lead); Data curation (supporting); Formal analysis (equal); Investigation (equal); Methodology (equal); Project administration (supporting); Supervision (lead); Validation (equal); Visualization (supporting); Writing – original draft (equal); Writing – review & editing (equal).

## CONFLICT OF INTEREST STATEMENT

Hannu Kankaanranta reports personal fees for lectures and consulting from AstraZeneca, Boehringer‐Ingelheim, Chiesi Pharma, GSK, MSD, Novartis, Orion Pharma and Sanofi Genzyme outside the current work. The other authors declare no conflict of interest in the context of this work.

## Supporting information

Supporting Information S1

## Data Availability

Code and data needed to reproduce our findings are available freely at https://osf.io/fp3rw/.

## References

[1] Sweeney A , Sampath V , Nadeau KC . Early intervention of atopic dermatitis as a preventive strategy for progression of food allergy. Allergy Asthma Clin Immunol. 2021;17(1):30. 10.1186/s13223-021-00531-8 33726824 PMC7962338

[2] Bieber T . Atopic dermatitis. Ann Dermatol. 2010;22(2):125‐137. 10.5021/ad.2010.22.2.125 20548901 PMC2883413

[3] Kowalska‐Olędzka E , Czarnecka M , Baran A . Epidemiology of atopic dermatitis in Europe. J Drug Assess. 2019;8(1):126‐128. 10.1080/21556660.2019.1619570 31232396 PMC6566979

[4] Hadi HA , Tarmizi AI , Khalid KA , Gajdács M , Aslam A , Jamshed S . The epidemiology and global burden of atopic dermatitis: a narrative review. Life (Basel). 2021;11(9):936. 10.3390/life11090936 34575085 PMC8470589

[5] Deckers IA , McLean S , Linssen S , Mommers M , van Schayck CP , Sheikh A . Investigating international time trends in the incidence and prevalence of atopic eczema 1990–2010: a systematic review of epidemiological studies. PLoS One. 2012;7(7):e39803. 10.1371/journal.pone.0039803 22808063 PMC3394782

[6] Waserman S , Bégin P , Watson W . IgE‐mediated food allergy. Allergy Asthma Clin Immunol. 2018;14(suppl 2):55. 10.1186/s13223-018-0284-3 30263035 PMC6156835

[7] Loh W , Tang MLK . The epidemiology of food allergy in the global context. Int J Environ Res Publ Health. 2018;15(9):2043. 10.3390/ijerph15092043 PMC6163515 30231558

[8] Warren CM , Jiang J , Gupta RS . Epidemiology and burden of food allergy. Curr Allergy Asthma Rep. 2020;20(2):6. 10.1007/s11882-020-0898-7 32067114 PMC7883751

[9] Sampath V , Abrams EM , Adlou B , et al. Food allergy across the globe. J Allergy Clin Immunol. 2021;148(6):1347‐1364. 10.1016/j.jaci.2021.10.018 34872649

[10] Savage J , Johns CB . Food allergy: epidemiology and natural history. Immunol Allergy Clin North Am. 2015;35(1):45‐59. 10.1016/j.iac.2014.09.004 25459576 PMC4254585

[11] Tham EH , Leung DYM . How different parts of the world provide new insights into food allergy. Allergy Asthma Immunol Res. 2018;10(4):290‐299. 10.4168/aair.2018.10.4.290 29949829 PMC6021584

[12] Prescott SL , Pawankar R , Allen KJ , et al. A global survey of changing patterns of food allergy burden in children. World Allergy Organ J. 2013;6(1):21. 10.1186/1939-4551-6-21 24304599 PMC3879010

[13] Tsakok T , Marrs T , Mohsin M , et al. Does atopic dermatitis cause food allergy? A systematic review. J Allergy Clin Immunol. 2016;137(4):1071‐1078. 10.1016/j.jaci.2015.10.049 26897122

[14] Bantz SK , Zhu Z , Zheng T . The atopic march: progression from atopic dermatitis to allergic rhinitis and asthma. J Clin Cell Immunol. 2014;5(2). 10.4172/2155-9899.1000202 PMC4240310 25419479

[15] Robison RG , Singh AM . Controversies in allergy: food testing and dietary avoidance in atopic dermatitis. J Allergy Clin Immunol Pract. 2019;7(1):35‐39. 10.1016/j.jaip.2018.11.006 30501976 PMC6312729

[16] Hill DA , Spergel JM . The atopic march: critical evidence and clinical relevance. Ann Allergy Asthma Immunol. 2018;120(2):131‐137. 10.1016/j.anai.2017.10.037 29413336 PMC5806141

[17] Goksör E , Loid P , Alm B , Åberg N , Wennergren G . The allergic march comprises the coexistence of related patterns of allergic disease not just the progressive development of one disease. Acta Paediatr. 2016;105(12):1472‐1479. 10.1111/apa.13515 27381249 PMC5129460

[18] Han H , Roan F , Ziegler SF . The atopic march: current insights into skin barrier dysfunction and epithelial cell‐derived cytokines. Immunol Rev. 2017;278(1):116‐130. 10.1111/imr.12546 28658558 PMC5492959

[19] Thomsen SF . Epidemiology and natural history of atopic diseases. Eur Clin Respir J. 2015;2doi(1):24642. 10.3402/ecrj.v2.24642 PMC4629767 26557262

[20] Graham F , Eigenmann PA . Atopic dermatitis and its relation to food allergy. Curr Opin Allergy Clin Immunol. 2020;20(3):305‐310. 10.1097/aci.0000000000000638 32109909

[21] Dor‐Wojnarowska A , Liebhart J , Miecielica J , et al. The impact of sex and age on the prevalence of clinically relevant sensitization and asymptomatic sensitization in the general population. Arch Immunol Ther Exp. 2017;65(3):253‐261. 10.1007/s00005-016-0425-7 PMC5434121 27652380

[22] David Boothe W , Tarbox JA , Tarbox MB . Atopic dermatitis: pathophysiology. Adv Exp Med Biol. 2017;1027:21‐37. 10.1007/978-3-319-64804-0_3 29063428

[23] Carter CA , Frischmeyer‐Guerrerio PA . The genetics of food allergy. Curr Allergy Asthma Rep. 2018;18(1):2. 10.1007/s11882-018-0756-z 29374367

[24] Sehgal VN , Srivastava G , Dogra S . Atopic dermatitis: current options and treatment plan. Skinmed. 2010;8(6):335‐344. 21413649

[25] Strachan DP . Hay fever, hygiene, and household size. BMJ. 1989;299(6710):1259‐1260. 10.1136/bmj.299.6710.1259 2513902 PMC1838109

[26] Lisik D , Ioannidou A , Milani G , et al. Sibship size, birth order and risk of asthma and allergy: protocol for a systematic review and meta‐analysis. BMJ Open. 2021;11(8):e045795. 10.1136/bmjopen-2020-045795 PMC8383851 34426461

[27] Moher D , Shamseer L , Clarke M , et al. Preferred reporting items for systematic review and meta‐analysis protocols (PRISMA‐P) 2015 statement. Syst Rev. 2015;4(1):1. 10.1186/2046-4053-4-1 25554246 PMC4320440

[28] Page MJ , McKenzie JE , Bossuyt PM , et al. The PRISMA 2020 statement: an updated guideline for reporting systematic reviews. BMJ. 2021;372:n71. 10.1136/bmj.n71 33782057 PMC8005924

[29] Stroup DF , Berlin JA , Morton SC , et al. Meta‐analysis of observational studies in epidemiology a proposal for reporting. JAMA. 2000;283(15):2008‐2012. 10.1001/jama.283.15.2008 10789670

[30] Bignardi D , Comite P , Mori I , et al. Allergen‐specific IgE: comparison between skin prick test and serum assay in real life. Allergol Select. 2019;3(1):9‐14. 10.5414/alx01891e 32176225 PMC7066680

[31] Heinzerling L , Mari A , Bergmann KC , et al. The skin prick test ‐ European standards. Clin Transl Allergy. 2013;3(1):3. 10.1186/2045-7022-3-3 23369181 PMC3565910

[32] Ansotegui IJ , Melioli G , Canonica GW , et al. IgE allergy diagnostics and other relevant tests in allergy, a World Allergy Organization position paper. World Allergy Organ J. 2020;13(2):100080. 10.1016/j.waojou.2019.100080 32128023 PMC7044795

[33] Haddaway NR , Collins AM , Coughlin D , Kirk S . The role of Google Scholar in evidence reviews and its applicability to grey literature searching. PLoS One. 2015;10(9):e0138237. 10.1371/journal.pone.0138237 26379270 PMC4574933

[34] Balk EM , Chung M , Chen ML , Chang LKW , Trikalinos TA . Data extraction from machine‐translated versus original language randomized trial reports: a comparative study. Syst Rev. 2013/11/07;2(1):97. 10.1186/2046-4053-2-97 24199894 PMC4226266

[35] Bramer WM , Giustini D , de Jonge GB , Holland L , Bekhuis T . De‐duplication of database search results for systematic reviews in EndNote. J Med Libr Assoc. 2016;104(3):240‐243. 10.3163/1536-5050.104.3.014 27366130 PMC4915647

[36] Armijo‐Olivo S , Stiles CR , Hagen NA , Biondo PD , Cummings GG . Assessment of study quality for systematic reviews: a comparison of the Cochrane Collaboration Risk of Bias Tool and the Effective Public Health Practice Project Quality Assessment Tool: methodological research. J Eval Clin Pract. 2012;18(1):12‐18. 10.1111/j.1365-2753.2010.01516.x 20698919

[37] Smith M , Hosking J , Woodward A , et al. Systematic literature review of built environment effects on physical activity and active transport – an update and new findings on health equity. Int J Behav Nutr Phys Activ. 2017;14(1):158. 10.1186/s12966-017-0613-9 PMC5693449 29145884

[38] Hedges LV , Tipton E , Johnson MC . Robust variance estimation in meta‐regression with dependent effect size estimates. Res Synth Methods. 2010;1(1):39‐65. 10.1002/jrsm.5 26056092

[39] Fisher Z , Tipton E . Robumeta: An R‐Package for Robust Variance Estimation in Meta‐Analysis. 2015. 10.48550/ARXIV.1503.02220

[40] Ahn E , Kang H . Introduction to systematic review and meta‐analysis. Korean J Anesthesiol. 2018;71(2):103‐112. 10.4097/kjae.2018.71.2.103 29619782 PMC5903119

[41] Dayimu A . Forestploter: create flexible forest plot. 2022. https://cran.r–project.org/web/packages/forestploter/index.html

[42] Fu R , Gartlehner G , Grant M , et al. AHRQ methods for effective health care conducting quantitative synthesis when comparing medical interventions: AHRQ and the Effective Health Care Program. In: Methods Guide for Effectiveness and Comparative Effectiveness Reviews. Agency for Healthcare Research and Quality (US); 2008. 21433407

[43] WDI ‐ the world by income and region. Accessed November 24, 2022. https://datatopics.worldbank.org/world‐development‐indicators/the‐world‐by‐income‐and‐region.html

[44] Dalton JE , Bolen SD , Mascha EJ . Publication bias: the elephant in the review. Anesth Analg. 2016;123(4):812‐813. 10.1213/ane.0000000000001596 27636569 PMC5482177

[45] Viechtbauer W . Conducting meta‐analyses in R with the metafor package. J Stat Software. 2010;36(3):1‐48. 10.18637/jss.v036.i03

[46] Egger M , Smith GD , Schneider M , Minder C . Bias in meta‐analysis detected by a simple, graphical test. BMJ. 1997;315(7109):629‐634. 10.1136/bmj.315.7109.629 9310563 PMC2127453

[47] Martinez BAF , Leotti VB , Silva GSE , Nunes LN , Machado G , Corbellini LG . Odds ratio or prevalence ratio? An overview of reported statistical methods and appropriateness of interpretations in cross‐sectional studies with dichotomous outcomes in veterinary medicine. Front Vet Sci. 2017;4:193. 10.3389/fvets.2017.00193 29177157 PMC5686058

[48] George A , Stead TS , Ganti L . What's the risk: differentiating risk ratios, odds ratios, and hazard ratios? Cureus. 2020;12(8):e10047. 10.7759/cureus.10047 32983737 PMC7515812

[49] Montreuil B , Bendavid Y , Brophy J . What is so odd about odds? Can J Surg. 2005;48(5):400‐408. 16248140 PMC3211889

[50] Borenstein M , Higgins JP , Hedges LV , Rothstein HR . Basics of meta‐analysis: I(2) is not an absolute measure of heterogeneity. Res Synth Methods. 2017;8(1):5‐18. 10.1002/jrsm.1230 28058794

[51] Parr NJ , Schweer‐Collins ML , Darlington TM , Tanner‐Smith EE . Meta‐analytic approaches for examining complexity and heterogeneity in studies of adolescent development. J Adolesc. 2019;77(1):168‐178. 10.1016/j.adolescence.2019.10.009 31739275 PMC6934259

[52] Metsälä J , Lundqvist A , Kaila M , Gissler M , Klaukka T , Virtanen SM . Maternal and perinatal characteristics and the risk of cow's milk allergy in infants up to 2 years of age: a case‐control study nested in the Finnish population. Am J Epidemiol. 2010;171(12):1310‐1316. 10.1093/aje/kwq074 20472571

[53] Venter C , Palumbo MP , Sauder KA , et al. Incidence and timing of offspring asthma, wheeze, allergic rhinitis, atopic dermatitis, and food allergy and association with maternal history of asthma and allergic rhinitis. World Allergy Organ J. 2021;14(3):100526. 10.1016/j.waojou.2021.100526 33767802 PMC7957150

[54] Al‐Hammadi S , Al‐Maskari F , Bernsen R . Prevalence of food allergy among children in Al‐Ain city, United Arab Emirates. Int Arch Allergy Immunol. 2010;151(4):336‐342. 10.1159/000250442 19851075

[55] Rangkakulnuwat P , Lao‐Araya M . The prevalence and temporal trends of food allergy among preschool children in Northern Thailand between 2010 and 2019. World Allergy Organ J. 2021;14(10):100593. 10.1016/j.waojou.2021.100593 34721755 PMC8521453

[56] Linden CC , Misiak RT , Wegienka G , et al. Analysis of allergen specific IgE cut points to cat and dog in the Childhood Allergy Study. Ann Allergy Asthma Immunol. 2011;106(2):153‐158.e2. 10.1016/j.anai.2010.11.004 21277517 PMC3072074

[57] Mohammad HR , Belgrave D , Kopec Harding K , Murray CS , Simpson A , Custovic A . Age, sex and the association between skin test responses and IgE titres with asthma. Pediatr Allergy Immunol. 2016;27(3):313‐319. 10.1111/pai.12534 26766520

[58] Kahlert J , Gribsholt SB , Gammelager H , Dekkers OM , Luta G . Control of confounding in the analysis phase ‐ an overview for clinicians. Clin Epidemiol. 2017;9:195‐204. 10.2147/clep.S129886 28408854 PMC5384727

[59] Yang JY , Webster‐Clark M , Lund JL , Sandler RS , Dellon ES , Stürmer T . Propensity score methods to control for confounding in observational cohort studies: a statistical primer and application to endoscopy research. Gastrointest Endosc. 2019;90(3):360‐369. 10.1016/j.gie.2019.04.236 31051156 PMC6715456

[60] Evaniew N , Nuttall J , Farrokhyar F , Bhandari M , Ghert M . What are the levels of evidence on which we base decisions for surgical management of lower extremity bone tumors? Clin Orthop Relat Res. 2014;472(1):8‐15. 10.1007/s11999-013-3311-1 24081669 PMC3889455

[61] Kim DH , Lim DH , Samra M , Kim EH , Kim JH . How accurate are the ISAAC questions for diagnosis of allergic rhinitis in Korean children? Int J Environ Res Publ Health. 2018;15(7):1527. 10.3390/ijerph15071527 PMC6068583 30029503

[62] Girolomoni G , de Bruin‐Weller M , Aoki V , et al. Nomenclature and clinical phenotypes of atopic dermatitis. Ther Adv Chronic Dis. 2021;12:20406223211002979. 10.1177/20406223211002979 33854747 PMC8010850

[63] Tokura Y , Hayano S . Subtypes of atopic dermatitis: from phenotype to endotype. Allergol Int. 2022;71(1):14‐24. 10.1016/j.alit.2021.07.003 34344611

[64] Roduit C , Frei R , Depner M , et al. Phenotypes of atopic dermatitis depending on the timing of onset and progression in childhood. JAMA Pediatr. 2017;171(7):655‐662. 10.1001/jamapediatrics.2017.0556 28531273 PMC5710337

[65] Foong RX , Dantzer JA , Wood RA , Santos AF . Improving diagnostic accuracy in food allergy. J Allergy Clin Immunol Pract. 2021;9(1):71‐80. 10.1016/j.jaip.2020.09.037 33429723 PMC7794657

[66] Okada H , Kuhn C , Feillet H , Bach JF . The 'hygiene hypothesis' for autoimmune and allergic diseases: an update. Clin Exp Immunol. 2010;160(1):1‐9. 10.1111/j.1365-2249.2010.04139.x PMC2841828 20415844

[67] van Ree R , Poulsen LK , Wong GW , Ballmer‐Weber BK , Gao Z , Jia X . Food allergy: definitions, prevalence, diagnosis and therapy. Zhonghua Yu Fang Yi Xue Za Zhi. 2015;49(1):87‐92. 25876505

