# Supplementary material for: Birth order, sibship size, and risk of atopic dermatitis, food allergy, and atopy: A systematic review and meta‐analysis
Source: Clin Transl Allergy. 2023 Jun 17;13(6):e12270. doi: 10.1002/clt2.12270 (PMC10276328; doi:10.1002/clt2.12270)
Supplement: Supplementary file 1 — Supporting Information S1 [file CLT2-13-e12270-s001.docx]

**Supplementary material**

**Birth order, sibship size, and risk of atopic dermatitis, food allergy, and atopy: a systematic review and meta-analysis**

**Daniil Lisik,^1,*^ Saliha Selin Özuygur Ermis,^1^ Athina Ioannidou,^1^ Gregorio Paolo Milani,^2,3^ Sungkutu Nyassi,^1^ Giulia Carla Immacolata Spolidoro,^3^ Hannu Kankaanranta,^1,4,5^ Emma Goksör,^6^ Göran Wennergren,^1,6^ Bright Ibeabughichi Nwaru^1,7^**

^1^ Krefting Research Centre, Department of Internal Medicine and Clinical Nutrition, Institute of Medicine, University of Gothenburg, Gothenburg, Sweden

^2^ Department of Clinical Science and Community Health, University of Milan, Milan, Italy

^3^ Pediatric Unit, Fondazione IRCCS Ca’ Granda Ospedale Maggiore Policlinico, Milan, Italy

^4^ Tampere University Respiratory Research Group, Faculty of Medicine and Health Technology, Tampere University, Tampere, Finland

^5^ Department of Respiratory Medicine, Seinäjoki Central Hospital, Seinäjoki, Finland

^6^ Department of Pediatrics, Sahlgrenska Academy at University of Gothenburg, Gothenburg, Sweden

^7^ Wallenberg Centre for Molecular and Translational Medicine, University of Gothenburg, Gothenburg, Sweden

* Corresponding author

[daniil.lisik@gmail.com](mailto:daniil.lisik@gmail.com)

**ORCID iD**

| **Daniil Lisik** | 0000-0002-0220-5961 |
| --- | --- |
| **Saliha Selin Özuygur Ermis** | 0000-0003-3507-773X |
| **Athina Ioannidou** | 0000-0003-4352-8553 |
| **Gregorio Paolo Milani** | 0000-0003-3829-4254 |
| **Sungkutu Nyassi** | 0000-0002-0036-810X |
| **Giulia Carla Immacolata Spolidoro** | 0000-0002-3365-1171 |
| **Hannu Kankaanranta** | 0000-0001-5258-0906 |
| **Emma Goksör** | 0000-0001-9595-1877 |
| **Göran Wennergren** | 0000-0002-7010-7191 |
| **Bright Ibeabughichi Nwaru** | 0000-0002-2876-6089 |

**Supplementary material**

[Tables 3](#_Toc136550960)

[Table E1. Preferred Reporting Items for Systematic Reviews and Meta-Analyses (PRISMA) 2020 checklist 3](#_Toc136550961)

[Table E2. Meta-analyses Of Observational Studies in Epidemiology (MOOSE) checklist 5](#_Toc136550962)

[Search strategy 6](#_Toc136550963)

[Table E3A. Search strategy for AMED 6](#_Toc136550964)

[Table E3B. Search strategy for: CABI; OAIster; Open Access Theses and Dissertations; Open Grey; ProQuest Dissertations & Theses Global; SciELO; WHO Global Index Medicus 7](#_Toc136550965)

[Table E3C. Search strategy for CINAHL 8](#_Toc136550966)

[Table E3D. Search strategy for EMBASE 9](#_Toc136550967)

[Table E3E. Search strategy for Google Scholar 10](#_Toc136550968)

[Table E3F. Search strategy for PsycINFO 11](#_Toc136550969)

[Table E3G. Search strategy for PubMed 12](#_Toc136550970)

[Table E3H. Search strategy for Scopus 13](#_Toc136550971)

[Table E3I. Search strategy for Web of Science 14](#_Toc136550972)

[Table E4. Quality appraisal 15](#_Toc136550973)

[Tables of characteristics 19](#_Toc136550974)

[Table E5A. Table of characteristics – atopic dermatitis 19](#_Toc136550975)

[Table E5B. Table of characteristics – food allergy 29](#_Toc136550976)

[Table E5C. Table of characteristics – allergic sensitization 31](#_Toc136550977)

[Table E6. Publication bias 37](#_Toc136550978)

[Table E7. Sensitivity analysis by quality appraisal and outcome assessment method 38](#_Toc136550979)

[Table E8. Sensitivity analysis by rho value used in the meta-analyses 39](#_Toc136550980)

[Figures 42](#_Toc136550981)

[Figure E1A. Forest plot (atopic dermatitis [current] by birth order) 42](#_Toc136550982)

[Figure E1B. Forest plot (atopic dermatitis [current] by sibship size) 43](#_Toc136550983)

[Figure E2A. Forest plot (atopic dermatitis [ever] by birth order) 44](#_Toc136550984)

[Figure E2B. Forest plot (atopic dermatitis [ever] by sibship size) 45](#_Toc136550985)

[Figure E3. Forest plot (food allergy [current] by birth order) 46](#_Toc136550986)

[Figure E4A. Forest plot (allergic sensitization [allergen-specific immunoglobulin E] by birth order) 47](#_Toc136550987)

[Figure E4B. Forest plot (allergic sensitization [allergen-specific immunoglobulin E] by sibship size) 48](#_Toc136550988)

[Figure E5A. Forest plot (allergic sensitization [skin prick test] by birth order) 49](#_Toc136550989)

[Figure E5B. Forest plot (allergic sensitization [skin prick test] by sibship size) 50](#_Toc136550990)

[Figure E6. Funnel plots (prior to trim-and-fill) 51](#_Toc136550991)

[Figure E7. Funnel plots (after trim-and-fill) 52](#_Toc136550992)

[References to all included studies 53](#_Toc136550993)

# Tables

## Table E1. Preferred Reporting Items for Systematic Reviews and Meta-Analyses (PRISMA) 2020 checklist

| **Section and Topic** | **Item #** | **Checklist item** | **Location where item is reported** |
| --- | --- | --- | --- |
| **TITLE** | | |  |
| Title | 1 | Identify the report as a systematic review. | Title page (p. 1), Abstract (p. 2), Introduction (p. 4) |
| **ABSTRACT** | | |  |
| Abstract | 2 | See the PRISMA 2020 for Abstracts checklist. | Abstract (p. 2) |
| **INTRODUCTION** | | |  |
| Rationale | 3 | Describe the rationale for the review in the context of existing knowledge. | Introduction (p. 4-5) |
| Objectives | 4 | Provide an explicit statement of the objective(s) or question(s) the review addresses. | Introduction (p. 4-5) |
| **METHODS** | | |  |
| Eligibility criteria | 5 | Specify the inclusion and exclusion criteria for the review and how studies were grouped for the syntheses. | Methods > Inclusion and exclusion criteria (p. 6) |
| Information sources | 6 | Specify all databases, registers, websites, organisations, reference lists and other sources searched or consulted to identify studies. Specify the date when each source was last searched or consulted. | Methods > Data sources and search strategy (p. 6-7) |
| Search strategy | 7 | Present the full search strategies for all databases, registers and websites, including any filters and limits used. | Methods > Data sources and search strategy (p. 6-7) |
| Selection process | 8 | Specify the methods used to decide whether a study met the inclusion criteria of the review, including how many reviewers screened each record and each report retrieved, whether they worked independently, and if applicable, details of automation tools used in the process. | Methods > Study selection and data extraction (p. 7) |
| Data collection process | 9 | Specify the methods used to collect data from reports, including how many reviewers collected data from each report, whether they worked independently, any processes for obtaining or confirming data from study investigators, and if applicable, details of automation tools used in the process. | Methods > Study selection and data extraction (p. 7) |
| Data items | 10a | List and define all outcomes for which data were sought. Specify whether all results that were compatible with each outcome domain in each study were sought (e.g. for all measures, time points, analyses), and if not, the methods used to decide which results to collect. | Methods > Study selection and data extraction (p. 7) |
|  | 10b | List and define all other variables for which data were sought (e.g. participant and intervention characteristics, funding sources). Describe any assumptions made about any missing or unclear information. | Methods > Study selection and data extraction (p. 7) |
| Study risk of bias assessment | 11 | Specify the methods used to assess risk of bias in the included studies, including details of the tool(s) used, how many reviewers assessed each study and whether they worked independently, and if applicable, details of automation tools used in the process. | Methods > Quality assessment (p. 7-8) |
| Effect measures | 12 | Specify for each outcome the effect measure(s) (e.g. risk ratio, mean difference) used in the synthesis or presentation of results. | Methods > Data synthesis and statistical analysis (p. 10) |
| Synthesis methods | 13a | Describe the processes used to decide which studies were eligible for each synthesis (e.g. tabulating the study intervention characteristics and comparing against the planned groups for each synthesis (item #5)). | Methods > Data synthesis and statistical analysis (p. 8) |
|  | 13b | Describe any methods required to prepare the data for presentation or synthesis, such as handling of missing summary statistics, or data conversions. | Methods > Data synthesis and statistical analysis (p. 10) |
|  | 13c | Describe any methods used to tabulate or visually display results of individual studies and syntheses. | Methods > Data synthesis and statistical analysis (p. 9) |
|  | 13d | Describe any methods used to synthesize results and provide a rationale for the choice(s). If meta-analysis was performed, describe the model(s), method(s) to identify the presence and extent of statistical heterogeneity, and software package(s) used. | Methods > Data synthesis and statistical analysis (p. 8-10) |
|  | 13e | Describe any methods used to explore possible causes of heterogeneity among study results (e.g. subgroup analysis, meta-regression). | Methods > Data synthesis and statistical analysis (p. 9) |
|  | 13f | Describe any sensitivity analyses conducted to assess robustness of the synthesized results. | Methods > Data synthesis and statistical analysis (p. 9-10) |
| Reporting bias assessment | 14 | Describe any methods used to assess risk of bias due to missing results in a synthesis (arising from reporting biases). | Methods > Data synthesis and statistical analysis (p. 9) |
| Certainty assessment | 15 | Describe any methods used to assess certainty (or confidence) in the body of evidence for an outcome. | Methods > Data synthesis and statistical analysis (p. 10) |
| **RESULTS** | | |  |
| Study selection | 16a | Describe the results of the search and selection process, from the number of records identified in the search to the number of studies included in the review, ideally using a flow diagram. | Results (p. 10) |
|  | 16b | Cite studies that might appear to meet the inclusion criteria, but which were excluded, and explain why they were excluded. | Figure 1 |
| Study characteristics | 17 | Cite each included study and present its characteristics. | Results (p. 10-11), Supplementary material (Table E5, References to all included studies) |
| Risk of bias in studies | 18 | Present assessments of risk of bias for each included study. | Results > Study characteristics (p. 10-11), Figure 3, Supplementary material (Table E4) |
| Results of individual studies | 19 | For all outcomes, present, for each study: (a) summary statistics for each group (where appropriate) and (b) an effect estimate and its precision (e.g. confidence/credible interval), ideally using structured tables or plots. | Figure 4-8, Figure E1A-E5B, Supplementary material (Table E5) |
| Results of syntheses | 20a | For each synthesis, briefly summarise the characteristics and risk of bias among contributing studies. | Results > Current atopic dermatitis (p. 11), Results > Ever atopic dermatitis (p. 11-12), Results > Current food allergy (p. 12), Results > Ever food allergy (p. 12-13), Results > Atopy (p. 13) |
|  | 20b | Present results of all statistical syntheses conducted. If meta-analysis was done, present for each the summary estimate and its precision (e.g. confidence/credible interval) and measures of statistical heterogeneity. If comparing groups, describe the direction of the effect. | Figure 4-8, Figure E1A-E5B |
|  | 20c | Present results of all investigations of possible causes of heterogeneity among study results. | Figure 4-8, Figure E1A-E5B |
|  | 20d | Present results of all sensitivity analyses conducted to assess the robustness of the synthesized results. | Results > Publication bias and sensitivity analysis (p. 13-14), Table E6-E8 |
| Reporting biases | 21 | Present assessments of risk of bias due to missing results (arising from reporting biases) for each synthesis assessed. | Results > Publication bias and sensitivity analysis (p. 13-14), Figure E6-E7 |
| Certainty of evidence | 22 | Present assessments of certainty (or confidence) in the body of evidence for each outcome assessed. | Results > Publication bias and sensitivity analysis (p. 13-14) |
| **DISCUSSION** | | |  |
| Discussion | 23a | Provide a general interpretation of the results in the context of other evidence. | Discussion > Summary of key findings (p. 12), Discussion > Interpretation of findings (p. 16-17) |
|  | 23b | Discuss any limitations of the evidence included in the review. | Discussion > Strengths and limitations (p. 15-16) |
|  | 23c | Discuss any limitations of the review processes used. | Discussion > Strengths and limitations (p. 15-16) |
|  | 23d | Discuss implications of the results for practice, policy, and future research. | Discussion > Clinical and research implications (p. 17-18) |
| **OTHER INFORMATION** | | |  |
| Registration and protocol | 24a | Provide registration information for the review, including register name and registration number, or state that the review was not registered. | Methods (p. 5) |
|  | 24b | Indicate where the review protocol can be accessed, or state that a protocol was not prepared. | Methods (p. 5) |
|  | 24c | Describe and explain any amendments to information provided at registration or in the protocol. | Discussion > Strengths and limitations (p. 15) |
| Support | 25 | Describe sources of financial or non-financial support for the review, and the role of the funders or sponsors in the review. | N/A |
| Competing interests | 26 | Declare any competing interests of review authors. | Conflict of interest (p. 18-19) |
| Availability of data, code and other materials | 27 | Report which of the following are publicly available and where they can be found: template data collection forms; data extracted from included studies; data used for all analyses; analytic code; any other materials used in the review. | Methods > Data synthesis and statistical analysis (p. 10) |

*From:*  Page MJ, McKenzie JE, Bossuyt PM, Boutron I, Hoffmann TC, Mulrow CD, et al. The PRISMA 2020 statement: an updated guideline for reporting systematic reviews. BMJ 2021;372:n71. doi: 10.1136/bmj.n71. For more information, visit: <http://www.prisma-statement.org/>.

##

## Table E2. Meta-analyses Of Observational Studies in Epidemiology (MOOSE) checklist

| **Item** | **Page number** |
| --- | --- |
| **Reporting of background** | |
| Problem definition | 4 |
| Hypothesis statement | 4-5 |
| Description of study outcome(s) | 6 |
| Type of exposure or intervention used | 6 |
| Type of study designs used | 6 |
| Study population | 6 |
| **Reporting of search strategy** | |
| Qualifications of searchers (eg, librarians and investigators) | N/A |
| Search strategy, including time period included in the synthesis and key words | 6-7 |
| Effort to include all available studies, including contact with authors | 6-7 |
| Databases and registries searched | 6-7 |
| Search software used, name and version, including special features used (eg, explosion) | 7, Table E3A-I |
| Use of hand searching (eg, reference lists of obtained articles) | 6-7 |
| List of citations located and those excluded, including justification | Supplementary material (References to all included studies), Figure 1 |
| Method of addressing articles published in languages other than English | 6 |
| Method of handling abstracts and unpublished studies | N/A |
| Description of any contact with authors | N/A |
| **Reporting of methods** | |
| Description of relevance or appropriateness of studies assembled for assessing the hypothesis to be tested | 7-8 |
| Rationale for the selection and coding of data (eg, sound clinical principles or convenience) | 7 |
| Documentation of how data were classified and coded (eg, multiple raters, blinding and interrater reliability) | 7-8 |
| Assessment of confounding (eg, comparability of cases and controls in studies where appropriate) | 8-10 |
| Assessment of study quality, including blinding of quality assessors, stratification or regression on possible predictors of study results | 7-8 |
| Assessment of heterogeneity | 9-10 |
| Description of statistical methods (eg, complete description of fixed or random effects models, justification of whether the chosen models account for predictors of study results, dose-response models, or cumulative meta-analysis) in sufficient detail to be replicated | 8-10 |
| Provision of appropriate tables and graphics | 8-9 |
| **Reporting of results** | |
| Graphic summarizing individual study estimates and overall estimate | Figure 4-8, Figure E1A-E5B |
| Table giving descriptive information for each study included | Table E5A-C |
| Results of sensitivity testing (eg, subgroup analysis) | 11-13, Figure 4-8 |
| Indication of statistical uncertainty of findings | 11-13, Figure 4-8, Figure E1A-E5B |
| **Reporting of discussion** | |
| Quantitative assessment of bias (eg, publication bias) | Figure E6-E7 |
| Justification for exclusion (eg, exclusion of non-English language citations) | Figure 1 |
| Assessment of quality of included studies | Table E4 |
| **Reporting of conclusions** | |
| Consideration of alternative explanations for observed results | 16-17 |
| Generalization of the conclusions (ie, appropriate for the data presented and within the domain of the literature review) | 16-17 |
| Guidelines for future research | 17-18 |
| Disclosure of funding source | N/A |

*From*: Stroup DF, Berlin JA, Morton SC, et al, for the Meta-analysis Of Observational Studies in Epidemiology (MOOSE) Group. Meta-analysis of Observational Studies in Epidemiology. A Proposal for Reporting. *JAMA*. 2000;283(15):2008-2012. doi: 10.1001/jama.283.15.2008.

## Search strategy

**Colorization**

Red: controlled vocabulary/thesaurus

Blue: free-text

Green: referral to search query component (table row; #)

### Table E3A. Search strategy for AMED

| **#** | **Search term(s)** |
| --- | --- |
| 1 | (birth order* or birth rank* or multiple birth* or parity).mp. |
| 2 | exp Family Characteristics/ or (family characteristic* or family size* or family structure* or family demograph* or family composition or household size* or household demograph* or household composition).mp. |
| 3 | exp Sibling Relations/ or (sibling* or sister* or brother* or sibship size* or sibship*).mp. |
| 4 | or/1-3 |
| 5 | exp Asthma/ or (bronchial asthma* or exercise-induced asthma* or exercise-induced bronchospasm* or asthma* or respiratory hypersensitivit* or airway hyper responsiveness or airway hyperresponsiveness or respiratory hyper responsiveness or respiratory hyper-responsiveness or wheez*).mp. |
| 6 | exp Hypersensitivity/ or exp Hypersensitivity Immediate/ or exp Hypersensitivity Delayed/ or (immediate hypersensitivit* or delayed hypersensitivit* or IgE-mediated hypersensitivit* or type I hypersensitivit* or type IV hypersensitivit* or atopic sensitization or atop* or allergic sensitization or allerg*).mp. |
| 7 | exp Dermatitis/ or exp Anaphylaxis/ or (atopic dermatitis or dermatitis or neurodermatiti* or besniers prurigo or besnier prurigo or atopic eczema or eczema or urticari* or anaphyla* or quinckes edema or quincke edema or angioneurotic edema or angioedema or hives).mp. |
| 8 | exp Food Hypersensitivity/ or (food hypersensitivit* or food allerg* or egg hypersensitivit* or egg allerg* or milk hypersensitivit* or milk allerg* or shellfish hypersensitivit* or shellfish allerg* or wheat hypersensitivit* or wheat allerg* or nut hypersensitivit* or nut allerg* or peanut hypersensitivit* or peanut allerg* or groundnut hypersensitivit* or groundnut allerg*).mp. |
| 9 | exp Rhinitis/ or exp Conjunctivitis/ or (allergic rhinoconjunctiviti* or rhinoconjunctiviti* or allergic rhiniti* or seasonal allergic rhiniti* or perennial allergic rhiniti* or rhiniti* or allergic conjunctiviti* or vernal keratoconjunctiviti* or vernal conjunctiviti* or giant papillary conjunctiviti* or hay fever or hayfever or pollinosis or nasal catarrh*).mp. |
| 10 | or/5-9 |
| 11 | 4 and 10 |
| **Full query**  ((birth order* or birth rank* or multiple birth* or parity).mp. or exp Family Characteristics/ or (family characteristic* or family size* or family structure* or family demograph* or family composition or household size* or household demograph* or household composition).mp. or exp Sibling Relations/ or (sibling* or sister* or brother* or sibship size* or sibship*).mp.) and (exp Asthma/ or (bronchial asthma* or exercise-induced asthma* or exercise-induced bronchospasm* or asthma* or respiratory hypersensitivit* or airway hyper responsiveness or airway hyperresponsiveness or respiratory hyper responsiveness or respiratory hyper-responsiveness or wheez*).mp. or exp  Hypersensitivity/ or exp Hypersensitivity Immediate/ or exp Hypersensitivity Delayed/ or (immediate hypersensitivit* or delayed hypersensitivit* or IgE-mediated hypersensitivit* or type I hypersensitivit* or type IV hypersensitivit* or atopic sensitization or atop* or allergic sensitization or allerg*).mp. or exp Dermatitis/ or exp Anaphylaxis/ or (atopic dermatitis or dermatitis or neurodermatiti* or besniers prurigo or besnier prurigo or atopic eczema or eczema or urticari* or anaphyla* or quinckes edema or quincke edema or angioneurotic edema or angioedema or hives).mp. or exp Food Hypersensitivity/ or (food hypersensitivit* or food allerg* or egg hypersensitivit* or egg allerg* or milk hypersensitivit* or milk allerg* or shellfish hypersensitivit* or shellfish allerg* or wheat hypersensitivit* or wheat allerg* or nut hypersensitivit* or nut allerg* or peanut hypersensitivit* or peanut allerg* or groundnut hypersensitivit* or groundnut allerg*).mp. or exp Rhinitis/ or exp Conjunctivitis/ or (allergic rhinoconjunctiviti* or rhinoconjunctiviti* or allergic rhiniti* or seasonal allergic rhiniti* or perennial allergic rhiniti* or rhiniti* or allergic conjunctiviti* or vernal keratoconjunctiviti* or vernal conjunctiviti* or giant papillary conjunctiviti* or hay fever or hayfever or pollinosis or nasal catarrh*).mp.) | |

exp = include all narrower subject headings; mp= abstract, heading words, title

### Table E3B. Search strategy for: CABI; OAIster; Open Access Theses and Dissertations; Open Grey; ProQuest Dissertations & Theses Global; SciELO; WHO Global Index Medicus

| **#** | **Search term(s)** |
| --- | --- |
| 1 | "birth order" OR "multiple births" OR "birth rank" OR "parity" |
| 2 | "family characteristics" OR "family size" OR "family structure" OR "family demography" OR "family composition" OR "household size" OR "household demography" OR "household composition" |
| 3 | "siblings" OR "sibling relations" OR "sister" OR "brother" OR "sibship" |
| 4 | "exercise-induced bronchospasm" OR "asthma" OR "airway hyper-responsiveness" OR "respiratory hyper-responsiveness" OR "wheeze" OR "wheezing" |
| 5 | ”hypersensitivity" OR "atopic sensitization" OR "atopy" OR "allergic sensitization" OR "allergic disease" OR "allergic condition" OR "allergy" OR "allergies" |
| 6 | "dermatitis" OR "eczema" OR "neurodermatitis" OR "besnier’s prurigo" OR "urticaria" OR "anaphylaxis" OR "anaphylactic shock" OR "quincke’s edema" OR "angionuerotic edema" OR "angioedema" OR "hives" |
| 7 | "rhinoconjunctivitis" OR "rhinitis" OR "allergic conjunctivitis" OR "vernal keratoconjunctivitis" OR "vernal conjunctivitis" OR "giant papillary conjunctivitis" OR "hay fever" OR "pollinosis" OR "pollenosis" OR "nasal catarrh" |
| 8 | 1 OR 2 OR 3 |
| 9 | 4 OR 5 OR 6 OR 7 |
| 10 | 8 AND 9 |
| **Full query**  ("birth order" OR "multiple births" OR "birth rank" OR "parity" OR "family characteristics" OR "family size" OR "family structure" OR "family demography" OR "family composition" OR "household size" OR "household demography" OR "household composition" OR "siblings" OR "sibling relations" OR "sister" OR "brother" OR "sibship") AND ("exercise-induced bronchospasm" OR "asthma" OR "airway hyper-responsiveness" OR "respiratory hyper-responsiveness" OR "wheeze" OR "wheezing" OR "hypersensitivity" OR "atopic sensitization" OR "atopy" OR "allergic sensitization" OR "allergic disease" OR "allergic condition" OR "allergy" OR "allergies" OR "dermatitis" OR "eczema" OR "neurodermatitis" OR "besnier’s prurigo" OR "urticaria" OR "anaphylaxis" OR "anaphylactic shock" OR "quincke’s edema" OR "angionuerotic edema" OR "angioedema" OR "hives" OR "rhinoconjunctivitis" OR "rhinitis" OR "allergic conjunctivitis" OR "vernal keratoconjunctivitis" OR "vernal conjunctivitis" OR "giant papillary conjunctivitis" OR "hay fever" OR "pollinosis" OR "pollenosis" OR "nasal catarrh") | |

### Table E3C. Search strategy for CINAHL

| **#** | **Search term(s)** |
| --- | --- |
| 1 | (MH ’Birth Order+’) OR (MH ’Parity+’) OR ’birth order*’ OR ’birth rank*’ OR ’multiple birth*’ OR ’parity’ |
| 2 | (MH ’Family Characteristics+’) OR (MH ’Family Health+’) OR ’family charactersitic*’ OR ’family size*’ OR ’family structure*’ OR ’family demograph*’ OR ’family composition’ OR ’household size*’ OR ’household demograph*’ OR ’household composition’ |
| 3 | (MH ’Siblings+’) OR (MH ’Sibling Relations+’) OR ’sibling*’ OR ’sister*’ OR ’brother*’ OR ’sibship size*’ OR ’sibship*’ |
| 4 | (MH ’Asthma+’) OR (MH ’Respiratory Hypersensitivity+’) OR ’bronchial asthma*’ OR ’exerciseinduced asthma’ OR ’asthma*’ OR ’exercise-induced bronchospasm’ OR ’respiratory hypersensitivit*’ OR ‘airway hyper responsiveness’ OR ’airway hyper-responsiveness’ OR ’respiratory hyper responsiveness’ OR ’respiratory hyper-responsiveness’ OR ’wheez*’ |
| 5 | (MH ’Hypersensitivity+’) OR (MH ’Hypersensitivity, Immediate+’) OR (MH ’Hypersensitivity, Delayed+’) OR (MH ’Allergy and Immunology+’) OR ’immedate hypersensitivit*’ OR ’delayed hypersensitivit*’ OR ’IgE-mediated hypersensitivit*’ OR ’type I hypersensitivit*’ OR ’type IV hypersensitivit*’ OR ’hypersensitivit*’ OR ’atopic sensitiziation’ OR ’atop*’ OR ’allergic sensitization’ OR ’allergic disease* OR ’allerg*’ |
| 6 | (MH ’Dermatitis, Atopic+’) OR (MH ’Eczema+’) OR (MH ‘Angioedema+’) OR (MH ‘Anaphylaxis+’) OR (MH ’Urticaria+’) OR ’atopic dermatitis’ OR ’dermatitis’ OR ’atopic eczema’ OR ’eczema’ OR ’nerudoarmatiti*’ OR “besnier’s prurigo” OR ‘besniers prurigo’ OR ‘besnier prurigo’ OR ’urticari*’ OR ’hives’ OR ’anaphyla*’ OR ”quincke’s edema” OR ’quinckes edema’ OR ’quincke edema’ OR ’angioneurotic edema’ OR ’angioedema’ |
| 7 | (MH ’Food Hypersensitivity+’) OR ’food hypersensitivit*’ OR ’food allerg*’ OR ’egg hypersensitivit*’ OR ’egg allerg*’ OR ’milk hypersensitivit*’ OR ’milk allerg*’ OR ’shellfish hypersensitivit*’ OR ’shellfish allerg*’ OR ’wheat hypersensitivit*’ OR ’wheat allerg*’ OR ’nut hypersensitivit*’ OR ’nut allerg*’ OR ’peanut hypersensitivit*’ OR ’peanut allerg*’ OR ’groundnut hypersensitivit*’ OR ’groundnut allerg*’ |
| 8 | (MH ’Rhinitis, Allergic+’) OR (MH ’Rhinitis, Allergic, Seasonal+’) OR (MH ’Rhinitis, Allergic, Perennial+’) OR (MH ’Rhinitis+’) OR (MH ’Conjunctivitis, Allergic+’) OR (MH ’Conjunctivitis+’) OR ’allergic rhinoconjunctiviti*’ OR ’rhinoconjunctiviti*’ OR ’allergic rhiniti*’ OR ’seasonal allergic rhiniti*’ OR ’perennial allergic rhiniti*’ OR ’rhiniti*’ OR ’allergic conjunctiviti*’ OR ’vernal keratoconjunctiviti*’ OR ’vernal conjunctiviti*’ OR ’giant papillary conjunctiviti*’ OR ’hay fever’ OR ’hayfever’ OR ’pollinosis’ OR ’pollenosis’ OR ’nasal catarrh’ |
| 9 | 1 OR 2 OR 3 |
| 10 | 4 OR 5 OR 6 OR 7 OR 8 |
| 11 | 9 AND 10 |
| **Full query**  ((MH "Birth Order+") OR (MH "Parity+") OR "birth order*" OR "birth rank*" OR "multiple birth*" OR "parity" OR (MH "Family Characteristics+") OR (MH "Family Health+") OR "family charactersitic*" OR "family size*" OR "family structure*" OR "family demograph*" OR "family composition" OR "household size*" OR "household demograph*" OR "household composition" OR (MH "Siblings+") OR (MH "Sibling Relations+") OR "sibling*" OR "sister*" OR "brother*" OR "sibship size*" OR "sibship*") AND ((MH "Asthma+") OR (MH "Respiratory Hypersensitivity+") OR "bronchial asthma*" OR "exercise-induced asthma" OR "asthma*" OR "exercise-induced bronchospasm" OR "respiratory hypersensitivit*" OR ‘airway hyper responsiveness" OR "airway hyper-responsiveness" OR "respiratory hyper responsiveness" OR "respiratory hyper-responsiveness" OR "wheez*" OR (MH "Hypersensitivity+") OR (MH "Hypersensitivity, Immediate+") OR (MH "Hypersensitivity, Delayed+") OR (MH "Allergy and Immunology+") OR "immedate hypersensitivit*" OR "delayed hypersensitivit*" OR "IgE-mediated hypersensitivit*" OR "type I hypersensitivit*" OR "type IV hypersensitivit*" OR "hypersensitivit*" OR "atopic sensitiziation" OR "atop*" OR "allergic sensitization" OR "allergic disease* OR "allerg*" OR (MH "Dermatitis, Atopic+") OR (MH "Eczema+") OR (MH ‘Angioedema+") OR (MH ‘Anaphylaxis+") OR (MH "Urticaria+") OR "atopic dermatitis" OR "dermatitis" OR "atopic eczema" OR "eczema" OR "nerudoarmatiti*" OR "besnier's prurigo" OR "besniers prurigo" OR "besnier prurigo" OR "urticari*" OR "hives" OR "anaphyla*" OR "quincke's edema" OR "quinckes edema" OR "quincke edema" OR "angioneurotic edema" OR "angioedema" OR (MH "Food Hypersensitivity+") OR "food hypersensitivit*" OR "food allerg*" OR "egg hypersensitivit*" OR "egg allerg*" OR "milk hypersensitivit*" OR "milk allerg*" OR "shellfish hypersensitivit*" OR "shellfish allerg*" OR "wheat hypersensitivit*" OR "wheat allerg*" OR "nut hypersensitivit*" OR "nut allerg*" OR "peanut hypersensitivit*" OR "peanut allerg*" OR "groundnut hypersensitivit*" OR "groundnut allerg*" OR (MH "Rhinitis, Allergic+") OR (MH "Rhinitis, Allergic, Seasonal+") OR (MH "Rhinitis, Allergic, Perennial+") OR (MH "Rhinitis+") OR (MH "Conjunctivitis, Allergic+") OR (MH "Conjunctivitis+") OR "allergic rhinoconjunctiviti*" OR "rhinoconjunctiviti*" OR "allergic rhiniti*" OR "seasonal allergic rhiniti*" OR "perennial allergic rhiniti*" OR "rhiniti*" OR "allergic conjunctiviti*" OR "vernal keratoconjunctiviti*" OR "vernal conjunctiviti*" OR "giant papillary conjunctiviti*" OR "hay fever" OR "hayfever" OR "pollinosis" OR "pollenosis" OR "nasal catarrh")) | |

MH = subject heading

### Table E3D. Search strategy for EMBASE

| **#** | **Search term(s)** |
| --- | --- |
| 1 | sibship.mp. |
| 2 | birth order.mp. or exp birth order/ |
| 3 | birth rank.mp. |
| 4 | multiple birth.mp. |
| 5 | parity.mp. or exp parity/ |
| 6 | family characteristic.mp. or exp family size/ |
| 7 | family structure.mp. |
| 8 | family demograph.mp. |
| 9 | family demograph*.mp. |
| 10 | family composition.mp. |
| 11 | household size.mp. |
| 12 | household demograph*.mp. |
| 13 | household composition.mp. |
| 14 | exp sibling relation/ or sibling.mp. or sibling/ |
| 15 | exp sister/ or sister.mp. |
| 16 | siblings.mp. |
| 17 | sisters.mp. |
| 18 | brother.mp. or exp brother/ |
| 19 | brothers.mp. |
| 20 | or/1-19 |
| 21 | exp Asthma/ or (bronchial asthma* or exercise-induced asthma* or exercise-induced bronchospasm* or asthma* or respiratory hypersensitivit* or airway hyper responsiveness or airway hyperresponsiveness or respiratory hyper responsiveness or respiratory hyper-responsiveness or wheez*).mp. |
| 22 | exp Hypersensitivity/ or exp Hypersensitivity Immediate/ or exp Hypersensitivity Delayed/ or (immediate hypersensitivit* or delayed hypersensitivit* or IgE-mediated hypersensitivit* or type I hypersensitivit* or type IV hypersensitivit* or atopic sensitization or atop* or allergic sensitization or allerg*).mp. |
| 23 | exp Dermatitis/ or exp Anaphylaxis/ or (atopic dermatitis or dermatitis or neurodermatiti* or besnier prurigo or besniers prurigo or besnier prurigo or atopic eczema or eczema or urticari* or anaphyla* or quincke edema or quinckes edema or quincke edema or angioneurotic edema or angioedema or hives).mp. |
| 24 | exp Food Hypersensitivity/ or (food hypersensitivit* or food allerg* or egg hypersensitivit* or egg allerg* or milk hypersensitivit* or milk allerg* or shellfish hypersensitivit* or shellfish allerg* or wheat hypersensitivit* or wheat allerg* or nut hypersensitivit* or nut allerg* or peanut hypersensitivit* or peanut allerg* or groundnut hypersensitivit* or groundnut allerg*).mp. |
| 25 | exp Rhinitis/ or exp Conjunctivitis/ or (allergic rhinoconjunctiviti* or rhinoconjunctiviti* or allergi rhiniti* or seasonal allergic rhiniti* or perennial allergic rhiniti* or rhiniti* or allergic conjunctiviti* or vernal keratoconjunctiviti* or vernal conjunctiviti* or giant papillary conjunctiviti* or hay fever or hayfever or pollinosis or pollinosis or nasal catarrh*).mp. |
| 26 | or/21-25 |
| 27 | 20 and 26 |

### Table E3E. Search strategy for Google Scholar

| **#** | **Search term(s)** |
| --- | --- |
| 1 | "family size" OR "family structure" OR "household size" |
| 2 | "sibling" OR "sibship" |
| 3 | "asthma" OR "wheezing" |
| 4 | "atopy" OR "allergy" |
| 5 | "eczema" OR "urticaria" OR "angioedema" OR "anaphylaxis" |
| 6 | "rhinitis" OR "allergic conjunctivitis" OR "hay fever" |
| 7 | 1 OR 2 |
| 8 | 3 OR 4 OR 5 OR 6 |
| 9 | 7 AND 8 |
| **Full query**  ("family size" OR "family structure" OR "household size" OR "sibling" OR "sibship") AND ("asthma" OR "wheezing" OR "atopy" OR "allergy" OR "anaphylaxis" OR "eczema" OR "urticaria" OR "angioedema" OR "rhinitis" OR "allergic conjunctivitis" OR "hay fever") | |

### Table E3F. Search strategy for PsycINFO

| **#** | **Search term(s)** |
| --- | --- |
| 1 | SU.EXACT.EXPLODE("Birth Order") OR TI,AB(”birth order*”) OR TI,AB(”multiple birth*”) OR TI,AB(”birth rank*”) OR TI,AB(”parity”) |
| 2 | SU.EXACT.EXPLODE("Family Structure") OR SU.EXACT.EXPLODE("Family Size") OR TI,AB(”family characteristic*”) OR TI,AB(”family size*”) OR TI,AB(”family structure*”) OR TI,AB(”family demograph*”) OR TI,AB(”family composition”) OR TI,AB(”household size*”) OR TI,AB(”household demograph*”) OR TI,AB(”household composition”) |
| 3 | SU.EXACT.EXPLODE("Siblings") OR SU.EXACT.EXPLODE("Sibling Relations") OR TI,AB(”sibling*’”) OR TI,AB(”sister*”) OR TI,AB(”brother*”) OR TI,AB(”sibship size*”) OR TI,AB(”sibship size*”) OR TI,AB(”sibship*”) |
| 4 | SU.EXACT.EXPLODE("Asthma") OR TI,AB(”bronchial asthma*”) OR TI,AB(”exercise-induced asthma*”) OR TI,AB(”asthma*”) OR TI,AB(”exercise-induced bronchospasm*”) OR TI,AB(”respiratory hypersensitivit*”) OR TI,AB(”airway hyper responsiveness”) OR TI,AB(”airway hyper-responsiveness”) OR TI,AB(”respiratory hyperresponsiveness”) OR TI,AB(”respiratory hyper-responsiveness”) OR TI,AB(”wheez*”) |
| 5 | SU.EXACT.EXPLODE("Allergic Disorders") OR TI,AB(”immediate hypersensitivit*”) OR TI,AB(”delayed hypersensitivit*”) OR TI,AB(”hypersensitivit*”) OR TI,AB(”IgE-mediated hypersensitivit*”) OR TI,AB(”type I hypersensitivit*”) OR TI,AB(”type IV hypersensitivit*”) OR TI,AB(”atopic sensitization”) OR TI,AB(”atop*”) OR TI,AB(”allergic sensitization”) OR TI,AB(”allergic disease*”) OR TI,AB(”allerg*”) |
| 6 | SU.EXACT.EXPLODE("Allergic Skin Disorders") OR SU.EXACT.EXPLODE("Neurodermatitis") OR SU.EXACT.EXPLODE("Dermatitis") OR SU.EXACT.EXPLODE("Eczema") OR SU.EXACT.EXPLODE("Anaphylactic Shock") OR TI,AB(”atopic dermatitis”) OR TI,AB(”dermatitis”) OR TI,AB(”atopic eczema”) OR TI,AB(”eczema”) OR TI,AB(”neurodermatiti*”) OR TI,AB(”besnier’s prurigo”) OR TI,AB(”besniers prurigo”) OR TI,AB(”besnier prurigo”) OR TI,AB(”urticari*”) OR TI,AB(”hives”) OR TI,AB(”anaphyla*”) OR TI,AB(”quincke’s edema”) OR TI,AB(”quinckes edema”) OR TI,AB(”quincke edema”) OR TI,AB(”angioneurotic edema”) OR TI,AB(”angioedema”) |
| 7 | SU.EXACT.EXPLODE("Food Allergies") OR TI,AB(”food hypersensitivit*”) OR TI,AB(”food allerg*”) OR TI,AB(”egg hypersensitivit*”) OR TI,AB(”egg allerg*”) OR TI,AB(”milk hypersensitivit*”) OR TI,AB(”milk allerg*”) OR TI,AB(”shellfish hypersensitivit*”) OR TI,AB(”shellfish allerg*”) OR TI,AB(”wheat hypersensitivit*”) OR TI,AB(”wheat allerg*”) OR  TI,AB(”nut hypersensitivit*”) OR TI,AB(”nut allerg*”) OR TI,AB(”peanut hypersensitivit*”) OR TI,AB(”peanut allerg*”) OR TI,AB(”groundnut hypersensitivit*”) OR TI,AB(”groundnut allerg*”) |
| 8 | TI,AB(”allergic rhinoconjunctiviti*”) OR TI,AB(”rhinoconjunctiviti*”) OR TI,AB(”allergic rhiniti*”) OR TI,AB(”rhiniti*”) OR TI,AB(”seasonal allergic rhiniti*”) OR TI,AB(”perennial allergic rhiniti*”) OR TI,AB(”allergic conjunctiviti*”) OR TI,AB(”vernal keratoconjunctiviti*”) OR TI,AB(”vernal conjunctiviti*”) OR TI,AB(”giant papillary conjunctiviti*”) OR TI,AB(”hay fever”) OR TI,AB(”hayfever”) OR TI,AB(“pollinosis”) OR TI,AB(”pollenosis”) OR TI,AB(”nasal catarrh*”) |
| 9 | 1 OR 2 OR 3 |
| 10 | 4 OR 5 OR 6 OR 7 OR 8 |
| 11 | 9 AND 10 |
| **Full query**  (SU.EXACT.EXPLODE("Birth Order") OR TI,AB("birth order*") OR TI,AB("multiple birth*") OR TI,AB("birth rank*") OR TI,AB("parity") OR SU.EXACT.EXPLODE("Family Structure") OR SU.EXACT.EXPLODE("Family  Size") OR TI,AB("family characteristic*") OR TI,AB("family size*") OR TI,AB("family structure*") OR TI,AB("family demograph*") OR TI,AB("family composition") OR TI,AB("household size*") OR TI,AB("household demograph*") OR TI,AB("household composition") OR SU.EXACT.EXPLODE("Siblings") OR SU.EXACT.EXPLODE("Sibling Relations") OR TI,AB("sibling*") OR TI,AB("sister*") OR TI,AB("brother*") OR TI,AB("sibship size*") OR TI,AB("sibship size*") OR TI,AB("sibship*")) AND (SU.EXACT.EXPLODE("Asthma") OR TI,AB("bronchial asthma*") OR TI,AB("exercise-induced asthma*") OR TI,AB("asthma*") OR TI,AB("exerciseinduced bronchospasm*") OR TI,AB("respiratory hypersensitivit*") OR TI,AB("airway hyper responsiveness") OR TI,AB("airway hyper-responsiveness") OR TI,AB("respiratory hyper responsiveness") OR TI,AB("respiratory hyperresponsiveness") OR TI,AB("wheez*") OR SU.EXACT.EXPLODE("Allergic Disorders") OR TI,AB("immediate hypersensitivit*") OR TI,AB("delayed hypersensitivit*") OR TI,AB("hypersensitivit*") OR TI,AB("IgE-mediated hypersensitivit*") OR TI,AB("type I hypersensitivit*") OR TI,AB("type IV hypersensitivit") OR TI,AB("atopic sensitization") OR TI,AB("atop*") OR TI,AB("allergic sensitization") OR TI,AB("allergic disease*") OR TI,AB("allerg*") OR SU.EXACT.EXPLODE("Allergic Skin Disorders") OR SU.EXACT.EXPLODE("Neurodermatitis") OR SU.EXACT.EXPLODE("Dermatitis") OR SU.EXACT.EXPLODE("Eczema") OR SU.EXACT.EXPLODE("Anaphylactic Shock") OR TI,AB("atopic dermatitis") OR TI,AB("dermatitis") OR TI,AB("atopic eczema") OR TI,AB("eczema") OR TI,AB("neurodermatiti*") OR TI,AB("besnier’s prurigo") OR TI,AB("besniers prurigo") OR TI,AB("besnier prurigo") OR TI,AB("urticari*") OR TI,AB("hives") OR TI,AB("anaphyla*") OR TI,AB("quincke’s edema") OR TI,AB("quinckes edema") OR TI,AB("quincke edema") OR TI,AB("angioneurotic edema") OR TI,AB("angioedema") OR SU.EXACT.EXPLODE("Food Allergies") OR TI,AB("food hypersensitivit*") OR TI,AB("food allerg*") OR TI,AB("egg hypersensitivit*") OR TI,AB("egg allerg*") OR TI,AB("milk hypersensitivit*") OR TI,AB("milk allerg*") OR TI,AB("shellfish hypersensitivit*") OR TI,AB("shellfish allerg*") OR TI,AB("wheat hypersensitivit*") OR TI,AB("wheat allerg*") OR TI,AB("nut hypersensitivit*") OR TI,AB("nut allerg*") OR TI,AB("peanut hypersensitivit*") OR TI,AB("peanut allerg*") OR TI,AB("groundnut hypersensitivit*") OR TI,AB("groundnut allerg*") OR TI,AB("allergic rhinoconjunctiviti*") OR TI,AB("rhinoconjunctiviti*") OR TI,AB("allergic rhiniti*") OR TI,AB("rhiniti*") OR TI,AB("seasonal allergic rhiniti*") OR TI,AB("perennial allergic rhiniti*") OR TI,AB("allergic conjunctiviti*") OR TI,AB("vernal keratoconjunctiviti*") OR TI,AB("vernal conjunctiviti*") OR TI,AB("giant papillary conjunctiviti*") OR TI,AB("hay fever") OR TI,AB("hayfever") OR TI,AB(“pollinosis") OR TI,AB("pollenosis") OR TI,AB("nasal catarrh*")) | |

SU = all subjects and indexing; TI,AB = title, abstract

### Table E3G. Search strategy for PubMed

| **#** | **Search term(s)** |
| --- | --- |
| 1 | Birth Order[mh] OR Parity[mh] OR birth order*[tiab] OR multiple birth*[tiab] OR birth rank*[tiab] OR parity[tiab] |
| 2 | Family Characteristics[mh] OR Family Health[mh] OR family characteristic*[tiab] OR family size*[tiab] OR family structure*[tiab] OR family demograph*[tiab] OR family composition[tiab] OR household size*[tiab] OR household demograph*[tiab] OR household composition[tiab] |
| 3 | Siblings[mh] OR Sibling Relations[mh] OR sibling*[tiab] OR sister*[tiab] OR brother*[tiab] OR sibship size*[tiab] OR sibship*[tiab] |
| 4 | Asthma[mh] OR Asthma, Exercise-Induced[mh] OR Respiratory Hypersensitivity[mh] OR bronchial asthma*[tiab] OR exercise-induced asthma*[tiab] OR exercise-induced bronchospasm*[tiab] OR asthma*[tiab] OR respiratory hypersensitivit*[tiab] OR airway hyper responsiveness[tiab] OR airway hyper-responsiveness[tiab] OR respiratory hyper responsiveness[tiab] OR respiratory hyperresponsiveness[tiab] OR wheez*[tiab] |
| 5 | Hypersensitivity[mh] OR Hypersensitivity, Immediate[mh] OR Hypersensitivity, Delayed[mh] OR Allergy and Immunology[mh] OR Allergens / Immunology[mh] OR immediate hypersensitivit*[tiab] OR delayed hypersensitivit*[tiab] OR hypersensitivit*[tiab] OR IgE-mediated hypersensitivit*[tiab] OR type I hypersensitivit*[tiab] OR type IV hypersensitivit*[tiab] OR atopic sensitization[tiab] OR atop*[tiab] OR allergic sensitization[tiab] OR allergic disease*[tiab] OR allerg*[tiab] |
| 6 | Dermatitis, Atopic[mh] OR Eczema[mh] OR Angioedema[mh] OR Anaphylaxis[mh] OR Urticaria[mh] OR atopic dermatitis[tiab] OR dermatitis[tiab] OR atopic eczema[tiab] OR eczema[tiab] OR neurodermatiti*[tiab] OR besnier’s prurigo[tiab] OR besniers prurigo[tiab] OR besnier prurigo[tiab] OR urticari*[tiab] OR anaphyla*[tiab] OR quincke edema[tiab] OR quinckes edema[tiab] OR quincke’s edema[tiab] OR angioneurotic edema[tiab] OR angioedema[tiab] OR hives[tiab] |
| 7 | Food Hypersensitivity[mh] OR food hypersensitivit*[tiab] OR food allerg*[tiab] OR egg allerg*[tiab] OR egg hypersensitivit*[tiab] OR milk allerg*[tiab] OR milk hypersensitivit*[tiab] OR shellfish allerg*[tiab] OR shellfish hypersensitivit*[tiab] OR wheat allerg*[tiab] OR wheat hypersensitivit*[tiab] OR nut allerg*[tiab] OR nut hypersensitivit*[tiab] OR peanut allerg*[tiab] OR peanut hypersensitivit*[tiab] OR groundnut hypersensitivit*[tiab] |
| 8 | Pollen / Immunology[mh] OR Rhinitis, Allergic[mh] OR Rhinitis, Allergic, Seasonal[mh] OR Rhinitis, Allergic, Perennial[mh] OR Rhinitis[mh] OR Conjunctivitis, Allergic[mh] OR Conjunctivitis / Immunology[mh] OR Conjunctivitis / Epidemiology[mh] OR Conjunctivitis / Etiology[mh] OR allergic rhinoconjunctiviti*[tiab] OR rhinoconjunctiviti*[tiab] OR allergic rhiniti*[tiab] OR rhiniti*[tiab] OR seasonal allergic rhiniti*[tiab] OR perennial allergic rhiniti*[tiab] OR allergic conjunctiviti*[tiab] OR vernal keratoconjunctiviti*[tiab] OR vernal conjunctiviti*[tiab] OR giant papillary conjunctiviti*[tiab] OR hay fever[tiab] OR hayfever[tiab] OR pollinosis[tiab] OR pollenosis[tiab] OR nasal catarrh*[tiab] |
| 9 | 1 OR 2 OR 3 |
| 10 | 4 OR 5 OR 6 OR 7 OR 8 |
| 11 | 9 AND 10 |
| **Full query**  (Birth Order[mh] OR Parity[mh] OR birth order*[tiab] OR multiple birth*[tiab] OR birth rank*[tiab] OR parity[tiab] OR Family Characteristics[mh] OR Family Health[mh] OR family characteristic*[tiab] OR family size*[tiab] OR family structure*[tiab] OR family demograph*[tiab] OR family composition[tiab] OR household size*[tiab] OR household demograph*[tiab] OR household composition[tiab] OR Siblings[mh] OR Sibling Relations[mh] OR sibling*[tiab] OR sister*[tiab] OR brother*[tiab] OR sibship size*[tiab] OR sibship*[tiab]) AND (Asthma[mh] OR Asthma, Exercise-Induced[mh] OR Respiratory Hypersensitivity[mh] OR bronchial asthma*[tiab] OR exercise-induced asthma*[tiab] OR exercise-induced bronchospasm*[tiab] OR asthma*[tiab] OR respiratory hypersensitivit*[tiab] OR airway hyper responsiveness[tiab] OR airway hyper-responsiveness[tiab] OR respiratory hyper responsiveness[tiab] OR respiratory hyper-responsiveness[tiab] OR wheez*[tiab] OR Hypersensitivity[mh] OR Hypersensitivity, Immediate[mh] OR Hypersensitivity, Delayed[mh] OR Allergy and Immunology[mh] OR Allergens / Immunology[mh] OR immediate hypersensitivit*[tiab] OR delayed hypersensitivit*[tiab] OR hypersensitivit*[tiab] OR IgE-mediated hypersensitivit*[tiab] OR type I hypersensitivit*[tiab] OR type IV hypersensitivit*[tiab] OR atopic sensitization[tiab] OR atop*[tiab] OR allergic sensitization[tiab] OR allergic disease*[tiab] OR allerg*[tiab] OR Dermatitis, Atopic[mh] OR Eczema[mh] OR Angioedema[mh] OR Anaphylaxis[mh] OR Urticaria[mh] OR atopic dermatitis[tiab] OR dermatitis[tiab] OR atopic eczema[tiab] OR eczema[tiab] OR neurodermatiti*[tiab]OR besnier’s prurigo[tiab] OR besniers prurigo[tiab] OR besnier prurigo[tiab] OR urticari*[tiab] OR anaphyla*[tiab] OR quincke edema[tiab] OR quinckes edema[tiab] OR quincke’s edema[tiab] OR angioneurotic edema[tiab] OR angioedema[tiab] OR hives[tiab] OR Food Hypersensitivity[mh] OR food hypersensitivit*[tiab] OR food allerg*[tiab] OR egg allerg*[tiab] OR egg hypersensitivit*[tiab] OR milk allerg*[tiab] OR milk hypersensitivit*[tiab] OR shellfish allerg*[tiab] OR shellfish hypersensitivit*[tiab] OR wheat allerg*[tiab] OR wheat hypersensitivit*[tiab] OR nut allerg*[tiab] OR nut hypersensitivit*[tiab] OR peanut allerg*[tiab] OR peanut hypersensitivit*[tiab] OR groundnut hypersensitivit*[tiab] OR Pollen / Immunology[mh] OR Rhinitis, Allergic[mh] OR Rhinitis, Allergic, Seasonal[mh] OR Rhinitis, Allergic, Perennial[mh] OR Rhinitis[mh] OR Conjunctivitis, Allergic[mh] OR Conjunctivitis / Immunology[mh] OR Conjunctivitis / Epidemiology[mh] OR Conjunctivitis / Etiology[mh] OR allergic rhinoconjunctiviti*[tiab] OR rhinoconjunctiviti*[tiab] OR allergic rhiniti*[tiab] OR rhiniti*[tiab] OR seasonal allergic rhiniti*[tiab] OR perennial allergic rhiniti*[tiab] OR allergic conjunctiviti*[tiab] OR vernal keratoconjunctivitis*[tiab] OR vernal conjunctiviti*[tiab] OR giant papillary conjunctiviti*[tiab] OR hay fever[tiab] OR hayfever[tiab] OR pollinosis[tiab] OR pollenosis[tiab] OR nasal catarrh*[tiab]) | |

mh = MeSH; tiab = title, abstract

### Table E3H. Search strategy for Scopus

| **#** | **Search term(s)** |
| --- | --- |
| 1 | TITLE-ABS-KEY(”birth order*”) OR TITLE-ABS-KEY(”multiple birth*”) OR TITLE-ABS-KEY(”birth rank*”) OR TITLE-ABS-KEY(”parity”) |
| 2 | TITLE-ABS-KEY(”family characteristic*”) OR TITLE-ABS-KEY(”family size*”) OR TITLEABS-KEY(”family structure*”) OR TITLE-ABS-KEY(”family demograph*”) OR TITLE-ABS-KEY(”family composition”) OR TITLE-ABS-KEY(”household size*”) OR TITLE-ABS-KEY(”household demograph*”) OR TITLE-ABS-KEY(”household composition*”) |
| 3 | TITLE-ABS-KEY(”sibling relation*”) OR TITLE-ABS-KEY(”sibling*”) OR TITLE-ABSKEY(”brother*”) OR TITLE-ABS-KEY(”sister*”) OR TITLE-ABS-KEY(”sibship size*”) OR TITLE-ABS-KEY(”sibship*”) |
| 4 | TITLE-ABS-KEY(”bronchial asthma*”) OR TITLE-ABS-KEY(”exercise-induced asthma*”) OR TITLE-ABS-KEY(”asthma*”) OR TITLE-ABS-KEY(”exercise-induced bronchospasm*”) OR TITLE-ABS-KEY(”respiratory hypersensitivit*”) OR TITLE-ABS-KEY(”airway hyper responsiveness”) OR TITLE-ABS-KEY(”airway hyper-responsiveness”) OR TITLE-ABSKEY(”respiratory hyper responsiveness”) OR TITLE-ABS-KEY(”respiratory hyperresponsiveness”) OR TITLE-ABS-KEY(”wheez*”) |
| 5 | TITLE-ABS-KEY(”immediate hypersensitivit*”) OR TITLE-ABS-KEY(”delayed hypersensitivit*”) OR TITLE-ABS-KEY(”IgE-mediated hypersensitivit*”) OR TITLE-ABS-KEY(”type I hypersensitivit*”) OR TITLE-ABS-KEY(”type IV hypersensitivit*”) OR TITLE-ABS-KEY(”atopic sensitization”) OR TITLE-ABS-KEY(”atop*”) OR TITLE-ABS-KEY(”allergic sensitization”) OR TITLE-ABS-KEY(”allergic disease”) OR TITLE-ABS-KEY(”allerg*”) |
| 6 | TITLE-ABS-KEY(”atopic dermatitis”) OR TITLE-ABS-KEY(”dermatitis”) OR TITLE-ABS-KEY(”atopic eczema”) OR TITLE-ABS-KEY(”eczema”) OR TITLE-ABS-KEY(”neurodermatitis*”) OR TITLE-ABS-KEY(”besnier’s prurigo”) OR TITLE-ABS-KEY(”besniers prurigo”) OR TITLE-ABS-KEY(”besnier prurigo”) OR TITLE-ABSKEY(”quincke’s edema”) OR TITLE-ABS-KEY(”quinckes edema”) OR TITLE-ABSKEY(”quincke edema”) OR TITLE-ABS-KEY(”angioneurotic edema”) OR TITLE-ABS-KEY(”hives”) OR TITLE-ABS-KEY(”anaphyla*”) OR TITLE-ABS-KEY(”urticari*”) |
| 7 | TITLE-ABS-KEY(”food hypersensitivit*”) OR TITLE-ABS-KEY(”food allerg*”) OR TITLE-ABSKEY(”egg allerg*”) OR TITLE-ABS-KEY(”egg hypersensitivit*”) OR TITLE-ABS-KEY(”milk allerg*”) OR TITLE-ABS-KEY(”milk hypersensitivit*”) OR TITLE-ABS-KEY(”shellfish allerg*”) OR TITLE-ABS-KEY(”shellfish hypersensitivit*”) OR TITLE-ABS-KEY(”wheat allerg*”) OR TITLE-ABS-KEY(”wheat hypersensitivit*”) OR TITLE-ABS-KEY(”nut allerg*”) OR TITLE-ABS-KEY(”nut hypersensitivit*”) OR TITLE-ABS-KEY(”peanut allerg*”) OR TITLE-ABS-KEY(”peanut hypersensitivit*”) OR TITLE-ABS-KEY(”groundnut allerg*”) OR TITLE-ABS-KEY(”groundnut hypersensitivit*”) |
| 8 | TITLE-ABS-KEY(”allergic rhinoconjunctiviti*”) OR TITLE-ABS-KEY(”rhinoconjunctiviti*”) OR TITLE-ABS-KEY(”seasonal allergic rhiniti*”) OR TITLE-ABS-KEY(”perennial allergic rhiniti*”) OR TITLE-ABS-KEY(”allergic rhiniti*”) OR TITLE-ABS-KEY(”rhiniti*”) OR TITLE-ABSKEY(”allergic conjunctiviti*”) OR TITLE-ABS-KEY(”vernal keratoconjunctiviti*”) OR TITLE-ABS-KEY(”vernal conjunctiviti*”) OR TITLE-ABS-KEY(”giant papillary conjunctiviti*”) OR TITLE-ABS-KEY(”hay fever”) OR TITLE-ABS-KEY(”hayfever”) OR TITLE-ABS-KEY(”pollinosis”) OR TITLE-ABS-KEY(”pollenosis”) OR TITLE-ABS-KEY(”nasal catarrh*”) |
| 9 | 1 OR 2 OR 3 |
| 10 | 4 OR 5 OR 6 OR 7 OR 8 |
| 11 | 9 AND 10 |
| **Full query**  (TITLE-ABS-KEY(”birth order*”) OR TITLE-ABS-KEY(”multiple birth*”) OR TITLE-ABS-KEY(”birth rank*”) OR TITLE-ABS-KEY(”parity”) OR TITLE-ABS-KEY(”family characteristic*”) OR TITLE-ABS-KEY(”family size*”) OR TITLE-ABS-KEY(”family structure*”) OR TITLE-ABS-KEY(”family demograph*”) OR TITLE-ABS-KEY(”family composition”) OR TITLE-ABS-KEY(”household size*”) OR TITLE-ABS-KEY(”household demograph*”) OR TITLE-  ABS-KEY(”household composition*”) OR TITLE-ABS-KEY(”sibling relation*”) OR TITLE-ABS-KEY(”sibling*”) OR TITLE-ABS-KEY(”brother*”) OR TITLE-ABS-KEY(”sister*”) OR TITLE-ABS-KEY(”sibship size*”) OR TITLE-ABS-KEY(”sibship*”)) AND (TITLE-ABS-KEY(”bronchial asthma*”) OR TITLE-ABS-KEY(”exerciseinduced asthma*”) OR TITLE-ABS-KEY(”asthma*”) OR TITLE-ABS-KEY(”exercise-induced bronchospasm*”) OR TITLE-ABS-KEY(”respiratory hypersensitivit*”) OR TITLE-ABS-KEY(”airway hyper responsiveness”) OR TITLE-ABS-KEY(”airway hyper-responsiveness”) OR TITLE-ABS-KEY(”respiratory hyper responsiveness”) OR TITLEABS-KEY(”respiratory hyper-responsiveness”) OR TITLE-ABS-KEY(”wheez*”) OR TITLE-ABS-KEY(”immediate hypersensitivit*”) OR TITLE-ABS-KEY(”delayed hypersensitivit*”) OR TITLE-ABS-KEY(”IgE-mediated hypersensitivit*”) OR TITLE-ABS-KEY(”type I hypersensitivit*”) OR TITLE-ABS-KEY(”type IV hypersensitivit*”) OR TITLE-ABS-KEY(”atopic sensitization”) OR TITLE-ABS-KEY(”atop*”) OR TITLE-ABS-KEY(”allergic sensitization”) OR TITLE-ABS-KEY(”allergic disease”) OR TITLE-ABS-KEY(”allerg*”) OR TITLE-ABSKEY(”atopic dermatitis”) OR TITLE-ABS-KEY(”dermatitis”) OR TITLE-ABS-KEY(”atopic eczema”) OR TITLEABS-KEY(”eczema”) OR TITLE-ABS-KEY(”neurodermatitis*”) OR TITLE-ABS-KEY(”besnier’s prurigo”) OR TITLE-ABS-KEY(”besniers prurigo”) OR TITLE-ABS-KEY(”besnier prurigo”) OR TITLE-ABS-KEY(”quincke’s edema”) OR TITLE-ABS-KEY(”quinckes edema”) OR TITLE-ABS-KEY(”quincke edema”) OR TITLE-ABSKEY(”angioneurotic edema”) OR TITLE-ABS-KEY(”hives”) OR TITLE-ABS-KEY(”anaphyla*”) OR TITLE-ABS-KEY(”urticari*”) OR TITLE-ABS-KEY(”food hypersensitivit*”) OR TITLE-ABS-KEY(”food allerg*”) OR TITLE-ABS-KEY(”egg allerg*”) OR TITLE-ABS-KEY(”egg hypersensitivit*”) OR TITLE-ABS-KEY(”milk allerg*”) OR TITLE-ABS-KEY(”milk hypersensitivit*”) OR TITLE-ABS-KEY(”shellfish allerg*”) OR TITLE-ABS-KEY(”shellfish hypersensitivit*”) OR TITLE-ABS-KEY(”wheat allerg*”) OR TITLE-ABS-KEY(”wheat hypersensitivit*”) OR TITLE-ABS-KEY(”nut allerg*”) OR TITLE-ABS-KEY(”nut hypersensitivit*”) OR TITLE-ABS-KEY(”peanut allerg*”) OR TITLE-ABS-KEY(”peanut hypersensitivit*”) OR TITLE-ABS-KEY(”groundnut allerg*”) OR TITLE-ABS-KEY(”groundnut hypersensitivit*”) OR TITLE-ABS-KEY(”allergic rhinoconjunctiviti*”) OR TITLE-ABSKEY(”rhinoconjunctiviti*”) OR TITLE-ABS-KEY(”seasonal allergic rhiniti*”) OR TITLE-ABS-KEY(”perennial allergic rhiniti*”) OR TITLE-ABS-KEY(”allergic rhiniti*”) OR TITLE-ABS-KEY(”rhiniti*”) OR TITLE-ABSKEY(”allergic conjunctiviti*”) OR TITLE-ABS-KEY(”vernal keratoconjunctiviti*”) OR TITLE-ABS-KEY(”vernal conjunctiviti*”) OR TITLE-ABS-KEY(”giant papillary conjunctiviti*”) OR TITLE-ABS-KEY(”hay fever”) OR TITLE-ABS-KEY(”hayfever”) OR TITLE-ABS-KEY(”pollinosis”) OR TITLE-ABS-KEY(”pollenosis”) OR TITLE-ABS-KEY(”nasal catarrh*”)) | |

TITLE-ABS-KEY = title, abstract, keywords

### Table E3I. Search strategy for Web of Science

| **#** | **Search term(s)** |
| --- | --- |
| 1 | TS=”birth order*” OR TS=”multiple birth*” OR TS=”birth rank*” OR TS=”parity” |
| 2 | TS=”family characteristic*” OR TS=”family size*” OR TS=”family structure*” OR TS=”family demograph*” OR TS=”family composition” OR TS=”household size*” OR TS=”household demograph*” OR TS=”household composition” |
| 3 | TS=”sibling*” OR TS=”sister*” OR TS=”brother*” OR TS=”sibship size*” OR TS=”sibship*” |
| 4 | TS=”bronchial asthma*” OR TS=”exercise-induced asthma*” OR TS=”exercise-induced bronchospasm*” OR TS=”asthma*” OR TS=“respiratory hypersensitivit*” OR TS=“respiratory hyper-responsiveness*” OR TS=“airway hyper responsiveness*” OR TS=“airway hyperresponsiveness*” OR TS=wheez* |
| 5 | TS=”immediate hypersensitivit*” OR TS=”delayed hypersensitivit*” OR TS=”IgE-mediated hypersensitivit*” OR TS=”type I hypersensitivit*” OR TS=”type IV hypersensitivit*” OR TS=”hypersensitivit*” OR TS=”atopic sensitization” OR TS=”atop*” OR TS=”allergic sensitization” OR TS=”allergic disease*” OR TS=”allerg*” |
| 6 | TS=”atopic dermatitis” OR TS=”dermatitis” OR TS=”atopic eczema” OR TS=”eczema” OR TS=”neurodermatiti*” OR TS=”besnier’s prurigo” OR TS=”besniers prurigo” OR TS=”besnier prurigo” OR TS=”urticari*” OR TS=”anaphyla*” OR TS=”quincke’s edema” OR TS=”quinckes edema” OR TS=”quincke edema” OR TS=”angioneurotic edema” OR TS=”angioedema” OR TS=”hives” |
| 7 | TS=”food hypersensitivit*” OR TS=”food allerg*” OR TS=”egg allerg*” OR TS=”egg hypersensitivit*” OR TS=”milk allerg*” OR TS=”milk hypersensitivit*” OR TS=”shellfish allerg*” OR TS=”shellfish hypersensitivit*” OR TS=”wheat allerg*” OR TS=”wheat hypersensitivit*” OR TS=”nut allerg*” OR TS=”nut hypersensitivit*” OR TS=”peanut allerg*” OR TS=”peanut hypersensitivit*” OR TS=”groundnut allerg*” OR TS=”groundnut hypersensitivit*” |
| 8 | TS=”pollen allerg*” OR TS=”allergic rhinoconjunctiviti*” OR TS=”rhinoconjunctiviti*” OR TS=”seasonal allergic rhiniti*” OR TS=”perennial allergic rhiniti*” OR TS=”allergic rhiniti*” OR TS=”rhiniti*” OR TS=”allergic conjunctiviti*” OR TS=”vernal keratoconjunctiviti*” OR TS=”vernal conjunctiviti*” OR TS=”giant papillary conjunctiviti*” OR TS=”hay fever” OR TS=”hayfever” OR TS=”pollinosis” OR TS=”pollenosis” OR TS=”nasal catarrh*” |
| 9 | 1 OR 2 OR 3 |
| 10 | 4 OR 5 OR 6 OR 7 OR 8 |
| 11 | 9 AND 10 |
| **Full query**  (TS="birth order*" OR TS="multiple birth*" OR TS="birth rank*" OR TS="parity" OR TS="family characteristic*" OR TS="family size*" OR TS="family structure*" OR TS="family demograph*" OR TS="family composition" OR TS="household size*" OR TS="household demograph*" OR TS="household composition" OR TS="sibling*" OR TS="sister*" OR TS="brother*" OR TS="sibship size*" OR TS="sibship*") AND (TS="bronchial asthma*" OR TS="exercise-induced asthma*" OR TS="exercise-induced bronchospasm*" OR TS="asthma*" OR TS=“respiratory hypersensitivit*" OR TS=“respiratory hyper-responsiveness*" OR TS=“airway hyper responsiveness*" OR TS=“airway hyper-responsiveness*" OR TS=wheez* OR TS="immediate hypersensitivit*" OR TS="delayed hypersensitivit*" OR TS="IgE-mediated hypersensitivit*" OR TS="type I hypersensitivit*" OR TS="type IV hypersensitivit*" OR TS="hypersensitivit*" OR TS="atopic sensitization" OR TS="atop*" OR TS="allergic sensitization" OR TS="allergic disease*" OR TS="allerg*" OR TS="atopic dermatitis" OR TS="dermatitis" OR TS="atopic eczema" OR TS="eczema" OR TS="neurodermatiti*" OR TS="besnier’s prurigo" OR TS="besniers prurigo" OR TS="besnier prurigo" OR TS="urticari*" OR TS="anaphyla*" OR TS="quincke’s edema" OR TS="quinckes edema" OR TS="quincke edema" OR TS="angioneurotic edema" OR TS="angioedema" OR TS="hives" OR TS="food hypersensitivit*" OR TS="food allerg*" OR TS="egg allerg*" OR TS="egg hypersensitivit*" OR TS="milk allerg*" OR TS="milk hypersensitivit*" OR TS="shellfish allerg*" OR TS="shellfish hypersensitivit*" OR TS="wheat allerg*" OR TS="wheat hypersensitivit*" OR TS="nut allerg*" OR TS="nut hypersensitivit*" OR TS="peanut allerg*" OR TS="peanut hypersensitivit*" OR TS="groundnut allerg*" OR TS="groundnut hypersensitivit*" OR TS="pollen allerg*" OR TS="allergic rhinoconjunctiviti*" OR TS="rhinoconjunctiviti*" OR TS="seasonal allergic rhiniti*" OR TS="perennial allergic rhiniti*" OR TS="allergic rhiniti*" OR TS="rhiniti*" OR TS="allergic conjunctiviti*" OR TS="vernal keratoconjunctiviti*" OR TS="vernal conjunctiviti*" OR TS="giant papillary conjunctiviti*" OR TS="hay fever" OR TS="hayfever" OR TS="pollinosis" OR TS="pollenosis" OR TS="nasal catarrh*") | |

TS = title, abstract, author keywords, Keywords Plus

## Table E4. Quality appraisal

| **Author year^reference^** | **Selection bias** | **Study design** | **Confounders** | **Blinding** | **Data collection methods** | **Withdrawals and drop-outs** | **Overall rating** |
| --- | --- | --- | --- | --- | --- | --- | --- |
| **Al-Hammadi 2009**^1^ | Moderate | Moderate | Strong | Moderate | Strong | Moderate | **Strong** |
| **Ayatollahi 2004**^2^ | Weak | Moderate | Weak | Moderate | Strong | Moderate | **Weak** |
| **Batllés-Garrido 2010**^3^ | Weak | Moderate | Strong | Moderate | Strong | Moderate | **Moderate** |
| **Bedolla-Barajas 2018**^4^ | Moderate | Moderate | Moderate | Moderate | Moderate | Moderate | **Strong** |
| **Bingol 2021**^5^ | Weak | Moderate | Weak | Weak | Strong | Moderate | **Weak** |
| **Bodner 1998**^6^ | Moderate | Moderate | Weak | Moderate | Strong | Moderate | **Moderate** |
| **Bodner 2000**^7^ | Moderate | Strong | Weak | Moderate | Strong | Moderate | **Moderate** |
| **Brooks 2004**^8^ | Moderate | Strong | Strong | Moderate | Moderate | Moderate | **Strong** |
| **Burr 1997**^9^ | Moderate | Strong | Moderate | Moderate | Strong | Strong | **Strong** |
| **Butland 1997**^10^ | Moderate | Strong | Weak | Moderate | Moderate | Weak | **Weak** |
| **Çelik 2002**^11^ | Moderate | Moderate | Moderate | Moderate | Strong | Moderate | **Strong** |
| **Chatenoud 2020**^12^ | Moderate | Strong | Strong | Moderate | Strong | Moderate | **Strong** |
| **Cooper 2014**^13^ | Moderate | Moderate | Moderate | Moderate | Strong | Moderate | **Strong** |
| **Cullinan 2003**^14^ | Moderate | Strong | Moderate | Moderate | Moderate | Weak | **Moderate** |
| **Davis 1981**^15^ | Moderate | Moderate | Weak | Moderate | Strong | Moderate | **Moderate** |
| **De Meer 2005**^16^ | Weak | Moderate | Moderate | Moderate | Strong | Moderate | **Moderate** |
| **Deng 2021**^17^ | Moderate | Strong | Weak | Moderate | Strong | Strong | **Moderate** |
| **Draaisma 2015**^18^ | Moderate | Moderate | Moderate | Moderate | Moderate | Moderate | **Strong** |
| **Farooqi 1998**^19^ | Moderate | Strong | Weak | Strong | Strong | Moderate | **Moderate** |
| **Foliaki 2008**^20^ | Weak | Moderate | Moderate | Moderate | Strong | Weak | **Weak** |
| **Forastiere 1997**^21^ | Strong | Moderate | Strong | Moderate | Strong | Weak | **Moderate** |
| **Gao 2019**^22^ | Moderate | Strong | Strong | Moderate | Moderate | Strong | **Strong** |
| **Genuneit 2013**^23^ | Moderate | Moderate | Moderate | Moderate | Strong | Weak | **Moderate** |
| **Gibbs 2004**^24^ | Moderate | Moderate | Weak | Moderate | Strong | Moderate | **Moderate** |
| **Goldstein 2005**^25^ | Weak | Strong | Weak | Moderate | Strong | Weak | **Weak** |
| **Gupta 2016**^26^ | Weak | Moderate | Moderate | Moderate | Strong | Moderate | **Moderate** |
| **Haileamlak 2005**^27^ | Moderate | Strong | Weak | Moderate | Strong | Moderate | **Moderate** |
| **Harris 2001**^28^ | Moderate | Strong | Weak | Moderate | Strong | Strong | **Moderate** |
| **Harrop 2007**^29^ | Moderate | Moderate | Moderate | Moderate | Moderate | Moderate | **Strong** |
| **Ho 2019**^30^ | Strong | Moderate | Strong | Strong | Moderate | Moderate | **Strong** |
| **Ho 2021**^31^ | Strong | Moderate | Strong | Moderate | Moderate | Moderate | **Strong** |
| **Jarvis 1997**^32^ | Moderate | Moderate | Moderate | Moderate | Moderate | Weak | **Moderate** |
| **Kansen 2020**^33^ | Weak | Moderate | Strong | Moderate | Moderate | Moderate | **Moderate** |
| **Karmaus 2001**^34^ | Weak | Strong | Moderate | Moderate | Strong | Strong | **Moderate** |
| **Kerkhof 2003**^35^ | Strong | Strong | Moderate | Moderate | Strong | Weak | **Moderate** |
| **Kerkhof 2005**^36^ | Weak | Strong | Strong | Moderate | Strong | Weak | **Weak** |
| **Kikkawa 2018**^37^ | Strong | Strong | Strong | Moderate | Moderate | Moderate | **Strong** |
| **Kim 2009**^38^ | Weak | Strong | Moderate | Moderate | Strong | Weak | **Weak** |
| **Kinra 2006**^39^ | Moderate | Moderate | Moderate | Weak | Moderate | Moderate | **Moderate** |
| **Koplin 2012**^40^ | Moderate | Strong | Moderate | Moderate | Strong | Weak | **Moderate** |
| **Kramer 2009**^41^ | Moderate | Strong | Strong | Moderate | Strong | Weak | **Moderate** |
| **Kusunoki 2012**^42^ | Moderate | Moderate | Strong | Moderate | Moderate | Moderate | **Strong** |
| **Kwon 2018**^43^ | Moderate | Moderate | Moderate | Weak | Strong | Moderate | **Moderate** |
| **Larsson 2008**^44^ | Moderate | Strong | Strong | Moderate | Moderate | Moderate | **Strong** |
| **Lee 2004**^45^ | Moderate | Strong | Moderate | Moderate | Strong | Moderate | **Strong** |
| **Lee 2012**^46^ | Moderate | Moderate | Moderate | Moderate | Strong | Moderate | **Strong** |
| **Lewis 1998**^47^ | Weak | Strong | Strong | Moderate | Moderate | Weak | **Weak** |
| **Lin 2019**^48^ | Strong | Moderate | Strong | Strong | Strong | Moderate | **Strong** |
| **Linneberg 2001**^49^ | Moderate | Moderate | Moderate | Moderate | Strong | Moderate | **Strong** |
| **Linneberg 2006**^50^ | Moderate | Strong | Moderate | Weak | Weak | Strong | **Weak** |
| **Loo 2015**^51^ | Moderate | Strong | Strong | Moderate | Moderate | Weak | **Moderate** |
| **Lukkarinen 2021**^52^ | Moderate | Strong | Weak | Moderate | Strong | Weak | **Weak** |
| **Lyons 2020**^53^ | Strong | Moderate | Moderate | Moderate | Strong | Moderate | **Strong** |
| **Matricardi 1998**^54^ | Moderate | Moderate | Moderate | Moderate | Strong | Moderate | **Strong** |
| **McKeever 2001**^55^ | Strong | Strong | Weak | Moderate | Strong | Strong | **Moderate** |
| **Metsälä 2010**^56^ | Strong | Strong | Moderate | Moderate | Strong | Moderate | **Strong** |
| **Miyake 2004**^57^ | Moderate | Moderate | Strong | Moderate | Moderate | Moderate | **Strong** |
| **Miyake 2011**^58^ | Moderate | Moderate | Strong | Moderate | Moderate | Moderate | **Strong** |
| **Mommers 2004**^59^ | Moderate | Strong | Strong | Moderate | Moderate | Moderate | **Strong** |
| **Moncayo 2010**^60^ | Strong | Moderate | Moderate | Moderate | Moderate | Moderate | **Strong** |
| **Musgrove 1976**^61^ | Moderate | Strong | Weak | Moderate | Strong | Moderate | **Moderate** |
| **Nakamura 2000**^62^ | Strong | Moderate | Strong | Moderate | Moderate | Moderate | **Strong** |
| **Newson 2014**^63^ | Strong | Moderate | Moderate | Moderate | Moderate | Moderate | **Strong** |
| **Nicolaou 2008**^64^ | Moderate | Strong | Weak | Moderate | Strong | Strong | **Moderate** |
| **Nowak 1996**^65^ | Moderate | Moderate | Weak | Moderate | Strong | Moderate | **Moderate** |
| **Nystad 1998**^66^ | Weak | Moderate | Moderate | Moderate | Strong | Moderate | **Moderate** |
| **Ogbuanu 2010**^67^ | Moderate | Strong | Weak | Moderate | Strong | Weak | **Weak** |
| **Ohfuji 2009**^68^ | Moderate | Moderate | Moderate | Moderate | Moderate | Moderate | **Strong** |
| **Olesen 1997**^69^ | Strong | Strong | Weak | Strong | Strong | Weak | **Weak** |
| **Özmert 2009**^70^ | Weak | Moderate | Moderate | Moderate | Strong | Moderate | **Moderate** |
| **Palacios-Lopez 2001**^71^ | Weak | Moderate | Weak | Moderate | Strong | Moderate | **Weak** |
| **Parthasarathi 2021**^72^ | Moderate | Moderate | Strong | Moderate | Moderate | Moderate | **Strong** |
| **Pekkanen 1999**^73^ | Moderate | Moderate | Weak | Moderate | Strong | Moderate | **Moderate** |
| **Pekkanen 2001**^74^ | Moderate | Strong | Strong | Moderate | Strong | Weak | **Moderate** |
| **Perzanowski 2008**^75^ | Weak | Moderate | Weak | Moderate | Strong | Moderate | **Weak** |
| **Peters 2015**^76^ | Moderate | Moderate | Moderate | Moderate | Strong | Moderate | **Strong** |
| **Ponsonby 1998**^77^ | Strong | Moderate | Weak | Moderate | Moderate | Moderate | **Moderate** |
| **Ponsonby 2003**^78^ | Moderate | Strong | Moderate | Moderate | Strong | Strong | **Strong** |
| **Purvis 2005**^79^ | Moderate | Strong | Weak | Moderate | Strong | Moderate | **Moderate** |
| **Rangkakulnuwat 2021**^80^ | Moderate | Moderate | Weak | Moderate | Moderate | Moderate | **Moderate** |
| **Raukas-Kivioja 2006**^81^ | Strong | Moderate | Moderate | Moderate | Strong | Moderate | **Strong** |
| **Rönmark 2009**^82^ | Moderate | Strong | Moderate | Moderate | Strong | Strong | **Strong** |
| **Rönmark 2016**^83^ | Strong | Strong | Moderate | Moderate | Moderate | Moderate | **Strong** |
| **Rönmark 2017**^84^ | Moderate | Strong | Moderate | Moderate | Strong | Moderate | **Strong** |
| **Rutter 2020**^85^ | Strong | Moderate | Strong | Moderate | Moderate | Moderate | **Strong** |
| **Sardecka 2018**^86^ | Moderate | Moderate | Weak | Moderate | Strong | Moderate | **Moderate** |
| **Sasaki 2016**^87^ | Strong | Moderate | Moderate | Moderate | Moderate | Moderate | **Strong** |
| **Schäfer 2020**^88^ | Moderate | Strong | Moderate | Strong | Strong | Moderate | **Strong** |
| **Sears 1996**^89^ | Moderate | Strong | Weak | Moderate | Strong | Strong | **Moderate** |
| **Sheldrake 1976a**^90^ | Moderate | Moderate | Weak | Moderate | Moderate | Moderate | **Moderate** |
| **Sheldrake 1976b**^91^ | Moderate | Moderate | Weak | Moderate | Moderate | Moderate | **Moderate** |
| **Slob 2020**^92^ | Weak | Strong | Weak | Moderate | Strong | Weak | **Weak** |
| **Sozańska 2015**^93^ | Strong | Moderate | Moderate | Moderate | Strong | Moderate | **Strong** |
| **Stemeseder 2017**^94^ | Moderate | Moderate | Weak | Moderate | Strong | Moderate | **Moderate** |
| **Strachan 1989**^95^ | Strong | Strong | Weak | Moderate | Moderate | Weak | **Weak** |
| **Strachan 1996**^96^ | Moderate | Moderate | Weak | Moderate | Moderate | Moderate | **Moderate** |
| **Strachan 1997**^97^ | Moderate | Moderate | Moderate | Moderate | Strong | Moderate | **Strong** |
| **Strachan 2015**^98^ | Strong | Moderate | Weak | Moderate | Moderate | Moderate | **Moderate** |
| **Stråvik 2020**^99^ | Moderate | Strong | Weak | Moderate | Strong | Strong | **Moderate** |
| **Suaini 2021**^100^ | Moderate | Strong | Weak | Moderate | Strong | Moderate | **Moderate** |
| **Svanes 1999**^101^ | Strong | Moderate | Strong | Moderate | Strong | Weak | **Moderate** |
| **Svanes 2002**^102^ | Strong | Moderate | Weak | Moderate | Moderate | Moderate | **Moderate** |
| **Taylor-Robinson 2016**^103^ | Strong | Strong | Strong | Moderate | Moderate | Moderate | **Strong** |
| **Torfi 2015**^104^ | Weak | Moderate | Moderate | Moderate | Moderate | Moderate | **Moderate** |
| **Turner 2005**^105^ | Weak | Strong | Moderate | Moderate | Strong | Moderate | **Moderate** |
| **Venter 2021**^106^ | Moderate | Strong | Strong | Moderate | Strong | Moderate | **Strong** |
| **Victorino 2009**^107^ | Strong | Moderate | Moderate | Moderate | Strong | Moderate | **Strong** |
| **Von Linstow 2002**^108^ | Moderate | Moderate | Weak | Moderate | Strong | Moderate | **Moderate** |
| **Von Mutius 1994**^109^ | Moderate | Moderate | Weak | Moderate | Strong | Moderate | **Moderate** |
| **Xu 1999**^110^ | Strong | Strong | Moderate | Moderate | Moderate | Strong | **Strong** |
| **Yamazaki 2015**^111^ | Moderate | Moderate | Strong | Moderate | Strong | Moderate | **Strong** |
| **Zekveld 2006**^112^ | Moderate | Moderate | Weak | Moderate | Strong | Moderate | **Moderate** |
| **Ziyab 2019**^113^ | Moderate | Moderate | Moderate | Moderate | Moderate | Moderate | **Strong** |
| **Zutavern 2005**^114^ | Moderate | Moderate | Weak | Moderate | Strong | Moderate | **Moderate** |

## Tables of characteristics

### Table E5A. Table of characteristics – atopic dermatitis

|  | **Author year,^reference^ country** | **Study design** | **Years of data collection** | **Source of study population** | **Subjects analyzed^a^** | **Age (in years)^b^** | **Exposure and assessment method** | **Outcome assessment method** | **Main findings^c^** |
| --- | --- | --- | --- | --- | --- | --- | --- | --- | --- |
| 1 | **Ayatollahi 2004**,^2^ IR | Case-control | N/A | **Cases**  Physician-diagnosed atopic dermatitis  **Controls**  General population | 2,228 | 6-12 | *Birth order*  Questionnaire | Clinical assessment | **Physician-diagnosed atopic dermatitis (current)**  NS (N/A) |
| 2 | **Batllés-Garrido 2010**,^3^ ES | Cross-sectional | N/A | General population | 1,143 | 10-11 | *Birth order*  Questionnaire | Questionnaire | **Self-reported atopic dermatitis (ever)**  NS (N/A) |
| 3 | **Bedolla-Barajas 2018**,^4^ MX | Cross-sectional | 2012-2013 | General population | 1,003 | 6-7 | *Sibship size*  Questionnaire | Questionnaire | **Self-reported atopic dermatitis (current)**  NS (N/A) |
| 4 | **Bodner 1998**,^6^ GB | Cross-sectional | 1964 | General population | 2,111 | 10-14 | *Birth order, sibship size*  Questionnaire | Questionnaire | **Self-reported atopic dermatitis (ever)**  *Birth order*  NS (2-3 vs 1)  ↓ (≥4 vs 1)  *Sibship size*  NS (2-4 vs 1)  ↓ (≥5 vs 1) |
| 5 | **Brooks 2004**,^8^ JM | Cohort | 1986-1998 | General population | 1,040 | 11-12 | *Birth order*  Medical records | Questionnaire | **Self-reported atopic dermatitis (ever)**  NS (no. of older siblings*)* |
| 6 | **Butland 1997**,^10^ GB | Cohort | 1958-1986 | General population | 20,582 | ~16 | *Birth order*  Questionnaire | Questionnaire | **Self-reported atopic dermatitis (last year)**  ↓ (2-3 vs 1)  NS (4 vs 1)  ↓ (5 vs 1) |
| 7 | **Chatenoud 2020**,^12^ IT | Case-control | 2011-2014 | **Cases**  Physician-diagnosed atopic dermatitis  **Controls**  Routine pediatric follow-up/visits with no history of atopic dermatitis | 852 | 0.25-2 | *Birth order, sibship size*  Questionnaire | Clinical assessment | **Physician-diagnosed atopic dermatitis (current)**  *Birth order*  NS (youngest sibling's age ≥3 years)  ↓ (youngest sibling's age <3 years*)*  *Sibship size*  NS (2 vs 1)  ↓ (≥3 vs 1) |
| 8 | **Cooper 2014**,^13^ EC | Cross-sectional | 2005-2010 | General population | 6,821 | 5-16 | *Birth order*  Questionnaire | Questionnaire | **Self-reported atopic dermatitis (last year)**  NS (≥5 vs <5) |
| 9 | **De Meer 2005**,^16^ NL | Cross-sectional | 1997-1998 | General population | 1,555 | 8-13 | *Birth order*  Questionnaire | Questionnaire | **Self-reported atopic dermatitis (last year)**  NS (≥2 vs 1) |
| 10 | **Deng 2021**,^17^ CN | Cohort | 2018-2020 | General population | 10,085 | ~0.12 | *Birth order*  Questionnaire | Questionnaire | **Physician-diagnosed atopic dermatitis (ever)**  ↓ (≥2 vs 1) |
| 11 | **Draaisma 2015**,^18^ ES, HN, NL, SV | Cross-sectional | 2005-2007 | General population | 9,803 | ~1 | *Sibship size*  Questionnaire | Questionnaire | **Self-reported atopic dermatitis (first year of life)**  **Central America**  NS (≥2 vs 1)  **Europe**  ↓ (≥2 vs 1)  **All**  ↓ (≥2 vs 1) |
| 12 | **Farooqi 1998**,^19^ GB | Cohort | 1975-1996 | General population | 1,934 | 12-16 | *Birth order*  Medical records | Medical records | **Physician-diagnosed atopic dermatitis (ever)**  NS (≥2 vs 1) |
| 13 | **Foliaki 2008**,^20^ NC, PF, TK, WS | Cross-sectional | 2002-2003 | General population | 17,683 | 6-14 | *Birth order*  Questionnaire | Questionnaire | **Self-reported atopic dermatitis (last year)**  NS (≥2 vs 1) |
| 14 | **Gao 2019**,^22^ CN | Cohort | 2015-2016 | General population | 903 | ~1 | *Birth order*  Questionnaire | Questionnaire | **Physician-diagnosed or topical steroid-treated topic dermatitis (ever)**  NS (N/A) |
| 15 | **Gibbs 2004**,^24^ GB | Case-control | N/A | **Cases**  Medical records from general practitioners of topical steroid prescription and fulfilling the UK diagnostic criteria for atopic dermatitis  **Controls**  Medical records from general practitioners without topical steroid prescription and not fulfilling the UK diagnostic criteria for atopic dermatitis | 602 | 1-5 | *Birth order*  Questionnaire | Medical records and interview/clinical assessment | **Topical steroid prescription and clinical confirmation of atopic dermatitis (current)**  ↓ (≥2 vs 1) |
| 16 | **Goldstein 2005**,^25^ US | Cohort | N/A | Low-income population | 253 | ~3 | *Birth order*  Questionnaire | Questionnaire | **Physician-diagnosed atopic dermatitis (ever)**  *Birth order, at 12 months*  NS (≥2 vs 1)  *Birth order, at 24 months*  NS (≥2 vs 1)  *Birth order, at 36 months*  NS (≥2 vs 1) |
| 17 | **Haileamlak 2005**,^27^ ET | Nested case-control | 2003 | **Cases**  Self-reportd atopic dermatitis  **Controls**  General population | 7,915 | 1-5 | *Sibship size*  Questionnaire | Questionnaire | **Self-reported atopic dermatitis (last year)**  NS (≥3 vs 1-2) |
| 18 | **Harris 2001**,^28^ GB | Cohort | 1993-1995 | General population | 624 | ~2 | *Birth order*  Interview | Interview and clinical assessment | **Maternal-reported atopic dermatitis (last year)**  NS (≥2 vs 1)  **Clinically assessed atopic dermatitis (current)**  NS (≥2 vs 1)  **Maternal-report of physician-diagnosed atopic dermatitis (ever)**  NS (≥2 vs 1) |
| 19 | **Harrop 2007**,^29^ AU, BE, CH, DE, EE, ES, FR, GB, IS, IT, SE, NL, NO | Cross-sectional | 1998-2002 | General population | 8,206 | 27-56 | *Sibship size*  Questionnaire | Questionnaire | **Self-reported atopic dermatitis (last year)**  ↑ (2 vs 1)  NS (≥3 vs 1) |
| 20 | **Ho 2019**,^30^ TW | Cross-sectional | 2007-2008 | General population | 24,999 | 6-8 | *Birth order*  Questionnaire | Questionnaire | **Self-reported atopic dermatitis (last year)**  ↓ (≥2 vs 1)  NS (≥1 younger sibling vs none) |
| 21 | **Ho 2021**,^31^ TW | Cross-sectional | 2007 | General population | 23,630 | 6-8 | *Birth order*  Questionnaire | Questionnaire | **Self-reported atopic dermatitis (ever)**  ↓ (≥2 vs 1) |
| 22 | **Jarvis 1997**,^32^ GB | Cross-sectional | N/A | General population | 1,159 | 20-44 | *Birth order, sibship size*  Questionnaire | Questionnaire | **Self-reported atopic dermatitis (unknown timeframe)**  *Birth order*  NS (N/A)  *Sibship size*  NS (N/A) |
| 23 | **Kansen 2020**,^33^ NL | Cross-sectional | 2011-2019 | Outpatients registered for respiratory or allergic symptoms | 5,517 | 6 (3-10) | *Sibship size*  Questionnaire | Questionnaire | **Self-reported atopic dermatitis (last year)**  ↓ (≥2 vs 1) |
| 24 | **Kerkhof 2003**,^35^ NL | Cohort | 1996-1998 | **Cases**  Children of mothers with respiratory allergy or asthma and fulfilling criteria of atopic dermatitis  **Controls**  Children of mothers with respiratory allergy or asthma without history or clinical signs of atopic dermatitis | 304 | ~1 | *Sibship size*  Questionnaire | Clinician assessment and interview | **Fulfilled criteria of atopic dermatitis (in first year of life)**  NS (≥2 vs 1)  **Fulfilled criteria of atopic dermatitis with visible dermatitis on typical locations (current)**  NS (≥2 vs 1) |
| 25 | **Kikkawa 2018**,^37^ JP | Cohort | 2001-2015 | General population | 32,065 | ~12 | *Birth order*  Questionnaire | Questionnaire | **Physician-visit due to atopic dermatitis (last year)**  **6-18 months**  ↓ (≥2 vs 1)  **18-30 months**  ↑ (2 vs 1)  NS (≥3 vs 1)  **30-66 months**  ↑ (≥2 vs 1)  **66 months-8 years**  ↑ (2 vs 1)  NS (≥3 vs 1)  **8-9 years**  ↑ (2 vs 1)  **9-10 years**  ↑ (2 vs 1)  NS (≥3 vs 1)  **10-12 years**  NS (2 vs 1)  ↑ (≥3 vs 1) |
| 26 | **Kim 2009**,^38^ KR | Cohort | 2006-2007 | General population | 542 | ~1 | *Birth order*  Questionnaire | Clinical assessment | **Physician-diagnosed atopic dermatitis (current)**  NS (≥2 vs 1) |
| 27 | **Kinra 2006**,^39^ GB | Cross-sectional | 1948–1968 | General population | 14,140 | 16-30 | *Birth order, sibship size*  Questionnaire | Questionnaire | **Self-reported atopic dermatitis and/or urticaria (ever)**  *Birth order*  NS (≥2 vs 1)  *Sibship size*  NS (2 vs 1)  ↓ (≥3 vs 1) |
| 28 | **Kramer 2009**,^41^ BY | Cohort | 1996-2005 | General population | 13,889 | ~6.5 | *Birth order*  Questionnaire | Questionnaire | **Self-reported recurrent itchy rash (unknown timeframe)**  NS (≥2 vs 1) |
| 29 | **Kusunoki 2012**,^42^ JP | Cross-sectional | 2002 | General population | 11,454 | 7-15 | *Birth order*  Questionnaire | Questionnaire | **Self-reported atopic dermatitis (current)**  NS (≥2 vs 1)  **Self-reported atopic dermatitis (at age <1 year)**  ↓ (2 vs 1)  NS (≥3 vs 1) |
| 30 | **Kwon 2018**,^43^ KR | Case-control | 2008 | **Cases**  Males at conscription examination with physician-diagnosed atopic dermatitis  **Controls**  Males at conscription examination without atopic dermatitis | 19,048 | ~19 | *Sibship size*  Questionnaire | Clinical assessment | **Physician-diagnosed infancy-onset atopic dermatitis (current)**  NS (≥2 vs 1)  **Physician-diagnosed childhood-onset atopic dermatitis (current)**  NS (≥2 vs 1)  **Physician-diagnosed adolescent-onset atopic dermatitis (current)**  NS (≥2 vs 1) |
| 31 | **Larsson 2008**,^44^ SE | Cohort | 2000-2005 | General population | 4,779 | 6-9 | *Sibship size*  Questionnaire | Questionnaire | **Self-reported atopic dermatitis (during 5-year follow-up)**  NS (2 vs 1)  ↓ (≥3 vs 1) |
| 32 | **Lee 2004**,^45^ HK | Cohort | 1994-1995, 2000-2001 | General population | 4,448 | 6-7 | *Sibship size*  Questionnaire | Questionnaire | **Self-reported atopic dermatitis (ever)**  ↓ (≥3 vs 3) |
| 33 | **Lee 2012**,^46^ KR | Cross-sectional | 2008 | General population | 1,749 | 9-12 | *Birth order*  Questionnaire | Questionnaire | **Physician-diagnosed atopic dermatitis (current)**  ↑ (≥2 vs 1)  **Physician-diagnosed atopic dermatitis (ever)**  NS (≥2 vs 1) |
| 34 | **Lewis 1998**,^47^ GB | Cohort | 1970-1985 | General population | 17,427 | ~16 | *Birth order*  Interview | Interview | **Self-reported atopic dermatitis (last year)**  ↓ (2-3 vs 1)  NS (≥4 vs 1)  **Self-reported atopic dermatitis (ever)**  NS (≥2 vs 1) |
| 35 | **Lin 2019**,^48^ TW | Cohort | 2006-2015 | General population | 628,878 | ~6 | *Sibship size*  Official records | Medical records | **Medical records of ≥3 ambulatory or ≥1 hospital visit with ICD diagnosis code of atopic dermatitis (during 6-year follow-up)**  ↓ (≥2 vs 1) |
| 36 | **Linneberg 2006**,^50^ DK | Cohort | 1998-2004 | General population | 34,793 | ~1.5 | *Birth order*  Interview | Interview | **Self-reported atopic dermatitis (ever)**  ↑ (2 vs 1)  ↓ (≥3 vs 1) |
| 37 | **Loo 2015**,^51^ SG | Cohort | 2009-2012 | General population | 792 | ~1.5 | *Sibship size*  Interview | Interview | **Physician-diagnosed atopic dermatitis (diagnosed at age 0-18 months)**  ↓ (≥2 vs 1)  **Physician-diagnosed atopic dermatitis (diagnosed at age 12-18 months)**  NS (≥2 vs 1) |
| 38 | **McKeever 2001**,^55^ GB | Cohort | N/A | General population | 29,238 | 0-11 | *Birth order*  Medical records | Medical records | **Medical records of topic dermatitis diagnosis (during follow-up)**  Birth order  ↓ (≥2 vs 1)  NS (≥1 younger sibling vs 0)  NS (≥1 older sibling vs 0) |
| 39 | **Miyake 2004**,^57^ JP | Cross-sectional | 2001 | General population | 5,539 | 12-15 | *Birth order*  Questionnaire | Questionnaire | **Self-reported atopic dermatitis (last year)**  NS (≥2 vs 1) |
| 40 | **Miyake 2011**,^58^ JP | Cross-sectional | 2007-2008 | Pregnant women | 1,745 | 31.2 ± N/A | *Birth order, sibship size*  Questionnaire | Questionnaire | **Self-reported atopic dermatitis (last year)**  *Birth order*  NS (≥2 vs 1)  *Sibship size*  NS (≥2 vs 1) |
| 41 | **Musgrove 1976**,^61^ US | Cohort | N/A | Individuals diagnosed with infantile eczema | 99 | 16.17 ± N/A | *Birth order, sibship size*  Questionnaire | Clinical assessment and survey | **Persistent infantile eczema (current)**  *Birth order*  NS (≥2 vs 1)  *Sibship size*  NS (≥2 vs 1) |
| 42 | **Nakamura 2000**,^62^ JP | Cross-sectional | 1997 | General population | 3,856 | ~3 | *Birth order*  Questionnaire | Questionnaire | **Self-reported atopic dermatitis (current)**  NS (≥2 vs 1)  **Physician-diagnosed atopic dermatitis (ever)**  NS (≥2 vs 1) |
| 43 | **Nicolaou 2008**,^64^ GB | Cohort | 1995-2003 | General population | 922 | ~5 | *Birth order*  Questionnaire | Questionnaire | **Self-reported atopic dermatitis (ever)**  NS (≥2 vs 1) |
| 44 | **Ohfuji 2009**,^68^ JP | Cross-sectional | 2004-2005 | General population | 22,750 | 6-15 | *Birth order, sibship size*  Questionnaire | Questionnaire | **Self-reported atopic dermatitis (last year)**  *Birth order*  ↓ (≥3 vs 1)  *Sibship size*  ↓ (≥2 vs 1) |
| 45 | **Olesen 1997**,^69^ DK | Cohort | 1984-1992 | General population | 7,862 | 5.5-8.5 | *Birth order*  Medical records | Medical records and interview | **Physician-diagnosed atopic dermatitis (during follow-up)**  **General population**  ↓ (≥2 vs 1)  NS (3 vs 1)  ↓ (≥4 vs 1)  **Overrepresentation of preterm children**  NS (≥2 vs 1) |
| 46 | **Palacios-Lopez 2001**,^71^ MX | Case-control | 1971-1998 | **Cases**  Physician-diagnosed atopic dermatitis  **Controls**  Healthy children without personal or family history of atopy | 400 | 0-17 | *Birth order, sibship size*  Interview | Medical records | **Physician-diagnosed atopic dermatitis (current)**  *Birth order*  NS (N/A)  *Sibship size*  NS (N/A) |
| 47 | **Parthasarathi 2021**,^72^ IN | Cross-sectional | 2018 | General population | 7,804 | 9.6 ± 2.6 | *Sibship size*  Questionnaire | Questionnaire | **Atopic dermatitis (unknown timeframe)**  ↑ (≥2 vs 1) |
| 48 | **Pekkanen 1999**,^73^ FI | Cross-sectional | N/A | General population | 8,387 | 13-14 | *Birth order, sibship size*  Questionnaire | Questionnaire | **Self-reported atopic dermatitis (ever)**  *Birth order*  NS (≥2 vs 1)  *Sibship size*  NS (2-3 vs 1)  ↓ (≥4 vs 1)  **Self-reported atopic dermatitis (last year)**  *Birth order*  NS (≥2 vs 1)  *Sibship size*  NS (≥2 vs 1) |
| 49 | **Perzanowski 2008**,^75^ US | Cross-sectional | N/A | Low household income in areas with high prevalence of pediatric asthma | 1,005 | 1-6 | *Birth order*  Questionnaire | Questionnaire | **Self-reported atopic dermatitis (last year)**  NS (≥2 vs 1) |
| 50 | **Peters 2015**,^76^ AU | Cohort | 2007-2011 | General population | 5,276 | ~1 | *Sibship size*  Questionnaire | Questionnaire | **Physician-diagnosed atopic dermatitis (first year of life)**  NS (2 vs 1)  ↓ (3 vs 1)  NS (≥4 vs 1) |
| 51 | **Ponsonby 1998**,^77^ AU | Cross-sectional | 1995 | General population | 6,158 | ~7 | *Sibship size*  Questionnaire | Questionnaire | **Self-reported atopic dermatitis (ever)**  NS (≥2 vs 1) |
| 52 | **Purvis 2005**,^79^ NZ | Cohort | 1996-2001 | General population | 550 | ~3.5 | *Birth order*  Interview | Questionnaire | **Self-reported atopic dermatitis (last year)**  NS (≥2 vs 1) |
| 53 | **Rönmark 2016**,^83^ SE | Cross-sectional | 2008 | General population | 18,087 | 50.4 ± 15.4 | *Sibship size*  Questionnaire | Questionnaire | **Self-reported atopic dermatitis (last year)**  NS (2 vs 1)  ↓ (≥ 3 vs 1) |
| 54 | **Rutter 2020**,^85^ AR, BB, BE, BO, BR, CA, CI, CL, CN, CO, EC, EE, ES, GA, GB, HK, HU, ID, IM, IN, IR, KG, LT, MA, MK, MX, MY, NG, NZ, OM, PA, PE, PH, PL, PT, SD, SY, TH, TW, UY, VE, ZA | Cross-sectional | 2000-2003 | General population | 341,299 | 6-14 | *Sibship size*  Questionnaire | Questionnaire | **Self-reported atopic dermatitis (last year)**  **6-7 years old**  ↓ (≥3 vs 1-2)  **13-14 years old**  ↑ (≥3 vs 1-2) |
| 55 | **Sasaki 2016**,^87^ JP | Cross-sectional | 2012 | General population | 28,343 | 6-12 | *Sibship size*  Questionnaire | Questionnaire | **Self-reported atopic dermatitis (last year)**  NS (2 vs 1)  ↓ (≥3 vs 1) |
| 56 | **Sheldrake 1976a**,^90^ GB | Cross-sectional | 1974 | Male university students | 4,740 | N/A (adults) | *Birth order*  Questionnaire | Questionnaire | **Self-reported atopic dermatitis (last 5 years)**  NS (≥2 vs 1) |
| 57 | **Sheldrake 1976b**,^91^ GB | Cross-sectional | 1974 | Female university students | 3,282 | N/A (adults) | *Birth order*  Questionnaire | Questionnaire | **Self-reported atopic dermatitis (last 5 years)**  NS (≥2 vs 1) |
| 58 | **Slob 2020**,^92^ SE | Cohort | 1989-2016, 2005-2019 | General twin population | 43,281 | 3-10 | *Birth order*  Questionnaire | Questionnaire | **Physician-diagnosed atopic dermatitis (ever)**  NS (≥2 vs 1) |
| 59 | **Strachan 1989**,^95^ GB | Cohort | 1958-1982 | General population | 12,537 | ~23 | *Birth order*  N/A | Questionnaire | **Self-reported atopic dermatitis (first year of life)**  ↓ (≥3 vs 1) |
| 60 | **Strachan 2015**,^98^ AR, BB, BE, BO, BR, CA, CI, CL, CM, CN, CO, EC, EE, ES, ET, FI, FJ, GA, GB, HK, HU, ID, IM, IN, IR, JP, KG, KR, KW, LT, MA, MK, MT, MX, MY, NG, NZ, OM, PA, PE, PH, PL, PT, SD, SG, SY, TH, TK, TW, US, UY, VE, WS, ZA | Cross-sectional | 2000-2003 | General population | 547,426 | 6-14 | *Birth order, sibship size*  Questionnaire | Questionnaire | **Self-reported atopic dermatitis (last year)**  **6-7 years old, all**  *Birth order*  NS (per older sibling)  ↑ (per younger sibling)  *Sibship size*  NS (≥2 vs 1)  NS (per sibling)  **6-7 years old, high income**  *Sibship size*  NS (per sibling)  **6-7 years old, low-middle income**  *Sibship size*  NS (per sibling)  **13-14 years old, all**  *Birth order*  ↑ (per older sibling)  ↑ (per younger sibling)  *Sibship size*  ↓ (2 vs 1)  NS (≥3 vs 1)  NS (per sibling)  **13-14 years old, high income**  *Sibship size*  NS (per sibling)  **13-14 years old, low-middle income**  *Sibship size*  NS (per sibling)  **Self-reported atopic dermatitis (ever)**  **6-7 years old, all**  *Birth order*  ↓ (per older sibling)  ↑ (per younger sibling)  *Sibship size*  NS (2 vs 1)  ↓ (≥3 vs 1)  ↓ (per sibling)  **6-7 years old, high income**  *Sibship size*  ↓ (per sibling)  **6-7 years old, low-middle income**  *Sibship size*  ↓ (per sibling)  **13-14 years old, all**  *Birth order*  ↓ (per older sibling)  NS (per younger sibling)  *Sibship size*  NS (2 vs 1)  ↓ (≥3 vs 1)  **13-14 years old, high income**  *Sibship size*  ↓ (per sibling)  **13-14 years old, low-middle income**  *Sibship size*  NS (per sibling) |
| 61 | **Stråvik 2020**,^99^ SE | Cohort | 2015-2019 | General population | 508 | ~1 | *Birth order*  Questionnaire and medical records | Clinical assessment | **Physician-diagnosed atopic dermatitis (current)**  NS (≥2 vs 1) |
| 62 | **Svanes 2002**,^102^ AU, BE, CH, DE, EE, ES, FR, GB, IS, IT, SE, NL, NO | Cross-sectional | 1990-1995 | General population | 18,530 | 20-44 | *Sibship size*  Questionnaire | Questionnaire | **Self-reported atopic dermatitis (ever)**  NS (2-4 vs 1)  ↓ (≥5 vs 1) |
| 63 | **Taylor-Robinson 2016**,^103^ GB | Cohort | 2000-2007 | General population | 14,449 | ~5 | *Sibship size*  Questionnaire | Questionnaire | **Self-reported atopic dermatitis (ever)**  ↓ (≥2 vs 1) |
| 64 | **Torfi 2015**,^104^ IR | Cross-sectional | 2013-2014 | General population | 1,904 | 6-14 | *Birth order, sibship size*  Questionnaire | Questionnaire | **Self-reported atopic dermatitis (last year)**  NS (≥2 vs 1) |
| 65 | **Venter 2021**,^106^ US | Cohort | 2009-2020 | General population | 1,261 | 4-8 | *Birth order*  Questionnaire | Medical records | **Medical records of atopic dermatitis diagnosis (during follow-up)**  ↓ (≥2 vs 1) |
| 66 | **Victorino 2009**,^107^ US | Cross-sectional | 2003-2004 | General population | 102,353 | 0-17 | *Sibship size*  Questionnaire | Questionnaire | **Healthcare professional-diagnosed atopic dermatitis (last year)**  ↓ (no. of siblings) |
| 67 | **Xu 1999**,^110^ FI | Cohort | 1985-1993 | General population | 8,088 | ~7 | *Birth order*  Medical records | Questionnaire | **Self-reported atopic dermatitis (ever)**  ↓ (≥5 vs 1) |
| 68 | **Zutavern 2005**,^114^ DE | Cross-sectional | 1995-1996 | General population | 11,094 | 5-11 | *Birth order*  Questionnaire | Questionnaire and clinical assessment | **Self-reported atopic dermatitis (last year)**  ↑ (2 vs 1)  NS (≥3 vs 1)  **Physician-diagnosed atopic dermatitis (ever)**  ↑ (2 vs 1)  NS (≥3 vs 1)  **Visible flexural dermatitis in typical locations (current)**  NS (≥2 vs 1) |

**Column explanations. ^a^** The highest number of subjects in relevant analyses, or – when such numbers were not defined – the number of subjects included in the study. ^b^ Reported in years as either: (a) mean ± standard deviation; (b) minimum age-maximum age; (c) median age (interquartile range of age); or (d) ~approximate age. ^c^ Black bold titles indicate specific outcomes, blue bold titles indicate specific subgroups of subjects, and italic titles indicate analyses of specific exposure. For each analysis, the results are described as (a) NS (non-significant 95% confidence interval); (b) ↑ (significantly increased risk of outcome); or (c) ↓ (significantly decreased risk of outcome). In parenthesis, the sibship size or birth order from which the results are significant is indicated. **Country codes.** AU: Australia. BE: Belgium. BY: Belarus. CH: Switzerland. CN: China. DE: Germany. DK: Denmark. EC: Ecuador. EE: Estonia. ES: Spain. ET: Ethiopia. FI: Finland. FR: France. GB: United Kingdom of Great Britain and Northern Ireland. HK: Hong Kong. HN: Honduras. IN: India. IR: Iran (Islamic Republic of). IS: Iceland. IT: Italy. JM: Jamaica. JP: Japan. KR: Korea, Republic of. MX: Mexico. NC: New Caledonia. NL: Netherlands. NO: Norway. NZ: New Zealand. PF: French Polynesia. SE: Sweden. SG: Singapore. SV: El Salvador. TK: Tokelau. TW: Taiwan, Province of China. US: United States of America. WS: Samoa. **Other abbreviations.** ICD: International Classification of Diseases. N/A: not available. NS: non-significant.

### Table E5B. Table of characteristics – food allergy

|  | **Author year,^reference^ country** | **Study design** | **Years of data collection** | **Source of study population** | **Subjects analyzed^a^** | **Age (in years)^b^** | **Exposure and assessment method** | **Outcome assessment method** | **Main findings^c^** |
| --- | --- | --- | --- | --- | --- | --- | --- | --- | --- |
| 1 | **Al-Hammadi 2009**,^1^ AE | Cross-sectional | 2006 | General population | 397 | 6-9 | *Sibship size*  Questionnaire | Questionnaire | **Physician-diagnosed food allergy (ever)**  ↓ (≥6 vs 6) |
| 2 | **Bingol 2021**,^5^ TR | Cross-sectional | 2017-2019 | Children with physician-diagnosed food allergy | 1,248 | 0-18 | *Sibship size*  Questionnaire | Clinical assessment | **IgE-mediated food allergy vs other phenotypes, determined through sIgE/SPT and convincing history or positive OFC (current)**  ↑ (≥2 vs 1) |
| 3 | **Gao 2019**,^22^ CN | Cohort | 2015-2016 | General population | 903 | ~1 | *Birth order*  Questionnaire | Questionnaire | **Physician-diagnosed or self-reported food allergy (ever)**  ↓ (≥2 vs 1) |
| 4 | **Gupta 2016**,^26^ US | Cross-sectional | 2005-2011 | Children of families with ≥1 parent and ≥1 biologic child (age 0–21 years) with food allergy | 1,359 | 0-20 | *Sibship size*  Questionnaire | Clinical assessment | **Positive sIgE (≥0.35 kUA/l) or SPT with typical symptoms to that food (current)**  ↓ (no. of siblings) |
| 5 | **Kansen 2020**,^33^ NL | Cross-sectional | 2011-2019 | Children with outpatient records for respiratory or allergic symptoms | 5,517 | 6 (3-10) | *Sibship size*  Questionnaire | Questionnaire | **Self-reported food allergy (ever)**  NS (≥2 vs 1) |
| 6 | **Kikkawa 2018**,^37^ JP | Cohort | 2001-2015 | General population | 32,065 | ~12 | *Birth order*  Questionnaire | Questionnaire | **Physician-visit due to food allergy (last year)**  **6-30 months**  ↓ (≥2 vs 1)  **30-42 months**  NS (2 vs 1)  ↓ (≥3 vs 1)  **42-66 months**  ↓ (≥2 vs 1)  **66 months-10 years**  NS (≥2 vs 1)  **10-11 years**  ↓ (2 vs 1)  NS (≥3 vs 1)  **11-12 years**  NS (≥2 vs 1) |
| 7 | **Koplin 2012**,^40^ AU | Cohort | 2007-2011 | General population | 699 | ~1 | *Sibship size*  Questionnaire | Clinical assessment | **Egg allergy confirmed with positive SPT and OFC or recent clear reaction (current)**  ↓ (≥2 vs 1)  ↓ (per sibling) |
| 8 | **Kusunoki 2012**,^42^ JP | Cross-sectional | 2002 | General population | 11,454 | 7-15 | *Birth order*  Questionnaire | Questionnaire | **Self-reported food allergy (current)**  NS (≥2 vs 1)  **Self-reported food allergy (at age < 1 years)**  ↓ (2 vs 1)  NS (≥3 vs 1)  **Self-reported food allergy (at age** ≥**1 years)**  NS (2 vs 1)  ↓ (≥3 vs 1)  **Self-reported food allergy (at age < 1 years with tolerance before age 3 years)**  ↓ (≥2 vs 1)  **Self-reported food allergy (at age < 1 years with persistence at 3 years)**  NS (≥2 vs 1) |
| 9 | **Lukkarinen 2021**,^52^ FI | Cohort | 2011-2016 | General population | 1,976 | ~0.5 | *Birth order*  Questionnaire | Questionnaire | **Physician-diagnosed food allergy (ever)**  NS (≥2 vs 1) |
| 10 | **Metsälä 2010**,^56^ FI | Nested case-control | 1996-2006 | **Cases**  Physician-diagnosed cow's milk allergy  **Controls**  General population | 16,237 | ~2 | *Birth order*  Official records | Medical records | **Physician-diagnosed cow's milk allergy (ever)**  NS (2-4 vs 1)  ↓ (≥5 vs 1) |
| 11 | **Peters 2015**,^76^ AU | Cohort | 2007-2011 | General population | 5,276 | ~1 | *Sibship size*  Questionnaire | Clinical assessment | **Food allergy, confirmed with positive OFC and sIgE or SPT (current)**  **Single egg allergy**  ↓ (≥2 vs 1)  **Multiple food allergies, peanut-predominant**  NS (≥2 vs 1)  **Multiple food allergies, egg-predominant**  NS (≥2 vs 1) |
| 12 | **Rangkakulnuwat 2021**,^80^ TH | Cross-sectional | 2010-2019 | General population | 561 | 3-7 | *Sibship size*  Questionnaire | Questionnaire | **Self-reported food allergy (ever)**  NS (≥2 vs 1) |
| 13 | **Sardecka 2018**,^86^ PL | Case-control | 2014-2016 | **Cases**  Physician-diagnosed cow's milk allergy  **Controls**  General population | 239 | 0-1 | *Birth order, sibship size*  Questionnaire | Clinical assessment | **Cow's milk allergy, confirmed with improvement after elimination and positive OFC (current)**  *Birth order*  NS (≥2 vs 1)  *Sibship size*  NS (≥2 vs 1)  **Cow's milk allergy, IgE mediated vs non-IgE mediated, as per above (current)**  *Birth order*  NS (1 vs others)  *Sibship size*  ↓ (≥2 vs 1) |
| 14 | **Stravik 2020**,^99^ SE | Cohort | 2015-2019 | General population | 508 | ~1 | *Birth order*  Questionnaire or medical records | Clinical assessment | **Physician-diagnosed food allergy (current)**  NS (≥2 vs 1) |
| 15 | **Suaini 2021**,^100^ AU, SG | Cohort | 2007-2012 | General population | 1,192 | ~1.5 | *Sibship size*  Questionnaire | Clinical assessment | **Food allergy to egg or peanut, confirmed with positive SPT and history of allergic reaction (GUSTO) or positive OFC (HealthNuts) (current)**  **GUSTO**  NS (≥2 vs 1)  **HealthNuts**  NS (≥2 vs 1) |
| 16 | **Venter 2021**,^106^ US | Cohort | 2009-2020 | General population | 1,261 | 4-8 | *Birth order*  Questionnaire | Medical records | **Medical records of food allergy diagnosis (during follow-up)**  NS (≥2 vs 1) |
| 17 | **Victorino 2009**,^107^ US | Cross-sectional | 2003-2004 | General population | 102,353 | 0-17 | *Sibship size*  Questionnaire | Questionnaire | **Healthcare professional-diagnosed food allergy (last year)**  ↓ (no.of siblings) |
| 18 | **Ziyab 2019**,^113^ KW | Cross-sectional | 2016-2017 | General population | 3,738 | 11-14 | *Birth order*  Questionnaire | Questionnaire | **Self-reported food allergy (last year)**  ↓ (≥2 vs 1) |

**Column explanations. ^a^** The highest number of subjects in relevant analyses, or – when such numbers were not defined – the number of subjects included in the study. ^b^ Reported in years as either: (a) mean ± standard deviation; (b) minimum age-maximum age; (c) median age (interquartile range of age); or (d) ~approximate age. ^c^ Black bold titles indicate specific outcomes, blue bold titles indicate specific subgroups of subjects, and italic titles indicate analyses of specific exposure. For each analysis, the results are described as (a) NS (non-significant 95% confidence interval); (b) ↑ (significantly increased risk of outcome); or (c) ↓ (significantly decreased risk of outcome). In parenthesis, the sibship size or birth order from which the results are significant is indicated. **Country codes.** AE: United Arab Emirates. AU: Australia. CN: China. FI: Finland. JP: Japan. KW: Kuwait. NL: Netherlands. PL: Poland. SE: Sweden. SG: Singapore. TR: Turkey. TW: Taiwan, Province of China. US: United States of America. **Other abbreviations.** IgE: Immunoglobulin E. N/A: not available. NS: non-significant. OFC: Oral food challenge. sIgE: (allergen-)specific immunoglobulin E. SPT: Skin prick test.

### Table E5C. Table of characteristics – allergic sensitization

| **#** | **Author year,^reference^ country** | **Study design** | **Years of data collection** | **Source of study population** | **Subjects analyzed^a^** | **Age (in years)^b^** | **Exposure and assessment method** | **Outcome assessment method** | **Main findings^c^** |
| --- | --- | --- | --- | --- | --- | --- | --- | --- | --- |
| 1 | **Batllés-Garrido 2010**,^3^ ES | Cross-sectional | N/A | General population | 1,143 | 10-11 | *Birth order* Questionnaire | Clinical assessment | **Positive SPT to ≥1 of HDM, mold, cat, and grass and tree mixture (current)**  ↓ (≥2 vs 1) |
| 2 | **Bodner 2000**,^7^ GB | Nested case-control | 1995 | **Cases**  Self-reported adult-onset wheezing  **Controls**  General population | 319 | 39-45 | *Birth order, sibship size*  Questionnaire | Clinical assessment | **Atopic status based on cumulative measure of tIgE and positive SPT/RAST (≥0.35 IU/ml) test on HDM, cat, and grass mixture (current)**  NS (≥2 vs 1) |
| 3 | **Burr 1997**,^9^ GB | Cohort | N/A | Children with high risk of allergy | 440 | ~7 | *Birth order* Questionnaire | Clinical assessment | **Positive SPT to ≥1 of egg, milk, cat, HDM, and grass and mold mixture (current)**  NS (≥2 vs 1)  **Positive sIgE to ≥1 of HDM, cat, dog, mold, and grass and tree mixture (current)**  NS (≥2 vs 1) |
| 4 | **Çelik 2002**,^11^ TR | Case-control | 1999 | **Cases**  Positive SPT and asthma and/or allergic rhinitis  **Controls**  Negative SPT and no clinical signs of atopic disease | 319 | 32.5 ± 0.89 | *Sibship size*  Questionnaire | Clinical assessment | **Positive SPT to ≥1 of HDM, grass, tree, weed pollen, cat, dog, cockroach, and mold mixture (current)**  ↓ ≥4 (vs 1-2) |
| 5 | **Cooper 2014**,^13^ EC | Cross-sectional | 2005-2010 | General population | 6,821 | 5-16 | *Birth order* Questionnaire | Clinical assessment | **Positive SPT to ≥1 of HDM, cockroach, mold, cat, dog, and grass and mold mixture (current)**  NS (≥5 vs <5) |
| 6 | **Cullinan 2003**,^14^ GB | Cohort | 1993-2000 | General population | 1,050 | 28 ± 5 | *Birth order, sibship size*  Questionnaire | Clinical assessment | **Positive SPT to ≥1 of HDM, cat, and grass (current)**  *Birth order*  NS (per older sibling)  *Sibship size*  ↓ (no. of brothers)  NS (no. of sisters) |
| 7 | **Davis 1981**,^15^ GB | Case-control | N/A | **Cases**  Wheezing in last 10 years  **Controls**  General population | ~776 | 0-15 | *Sibship size*  Interview/questionnaire | Clinical assessment | **Positive SPT to ≥1 of pollen, mold, foods, HDM and domestic animal mixture (current)**  NS (≥3 vs 2) |
| 8 | **De Meer 2005**,^16^ NL | Cross-sectional | 1997-1998 | General population | 1,555 | 8-13 | *Birth order* Questionnaire | Clinical assessment | **Positive sIgE (≥0.35 kU/l) or SPT to ≥1 of cat, dog, HDM, and grass and tree and mold mixture (current)**  NS (≥2 vs 1) |
| 9 | **Forastiere 1997**,^21^ IT | Cross-sectional | 1987 | General population | 2,226 | 7-11 | *Sibship size*  Questionnaire | Clinical assessment | **Positive SPT to ≥1 of HDM, mugwort, grass, mold, cat, and tree mixture (current)**  ↓ (≥5 vs 1) |
| 10 | **Genuneit 2013**,^23^ AT, CH, DE | Cross-sectional | 2006-2007 | Rural population | 79,888 | 6-12 | *Sibship size*  Questionnaire | Clinical assessment | **Positive sIgE (≥0.7 kU/l) to ≥1 of HDM, cat, rye, timothy, mugwort, and common silver birch (current)**  **Farm children**  ↓ (≥3 vs 1)  **Non-farm children exposed to stables/barns, or consuming unprocessed milk**  ↑ (≥2 vs 1)  NS (≥4 vs 1)  **Non-farm children not exposed to stables/barns, or consuming unprocessed milk**  ↓ (≥2 vs 1) |
| 11 | **Jarvis 1997**,^32^ GB | Cross-sectional | N/A | General population | 1,159 | 20-44 | *Birth order, sibship size*  Questionnaire | Clinical assessment | **Positive sIgE (≥0.35 kUA/l) to ≥1 of HDM, cat, grass, birch, and mold (current)**  *Birth order*  NS (≥2 vs 1)  *Sibship size*  NS (≥2 vs 1)  **Positive sIgE (≥0.35 kUA/l) to grass (current)**  *Birth order*  NS (≥2 vs 1)  *Sibship size*  ↓ (per sibling) |
| 12 | **Karmaus 2001**,^34^ GB | Cohort | 1989-1994 | General population | 1,218 | ~4 | *Birth order* Questionnaire | Clinical assessment | **Positive SPT to ≥1 of HDM, grass mixture, cat, dog, mold, milk, egg, soybean, cod, wheat, and peanut (current)**  NS (≥2 vs 1) |
| 13 | **Kerkhof 2005**,^36^ NL | Cohort | 1996-2002 | General population | 1,027 | ~4 | *Birth order* Questionnaire | Clinical assessment | **Positive sIgE (≥0.35 IU/ml) to ≥1 of HDM, cat, dog, grass, milk, and egg (current)**  NS (≥2 vs 1) |
| 14 | **Kramer 2009**,^41^ BY | Cohort | 1996-2005 | General population | 13,889 | ~6.5 | *Birth order* Questionnaire | Clinical assessment | **Positive SPT to ≥1 of HDM, cat, birch, grass mixture, and mold (current)**  NS (≥2 vs 1) |
| 15 | **Lee 2012**,^46^ KR | Cross-sectional | 2008 | General population | 1,749 | 9-12 | *Birth order* Questionnaire | Clinical assessment | **Positive SPT to ≥ 1 of HDM, dog, cat, cockroach, grass, mold, ragweed, mugwort, milk, egg white, peanut, soybean, and tree mixture (current)**  ↓ (≥2 vs 1) |
| 16 | **Linneberg 2001**,^49^ DK | Cross-sectional | 1989-1991 | General population | 1,112 | 15-69 | *Sibship size* Questionnaire | Clinical assessment | **Positive sIgE (≥0.35 kU/l) to ≥1 of birch, timothy, mugwort, dog, cat, and HDM (current)**  ↓ (≥5 vs 1)  **Positive SPT to ≥1 of birch, timothy, mugwort, horse, cat, dog, HDM, and mold (current)**  ↓ (≥5 vs 1) |
| 17 | **Lyons 2020**,^53^ CH, ES, GR, IS, LT, NL, PL | Case-control | 2006-2009 | General population | 4,381 | 7-10, 20-54 | *Birth order, sibship size*  Questionnaire | Clinical assessment | **Positive sIgE (≥0.35 kUA/l) to ≥1 of 24 selected food allergens (hen's egg, cow's milk, fish, shrimp, peanut, hazelnut, walnut, peach, apple, kiwi, melon, banana, tomato, celery, carrot, corn, lentils, soy, wheat, buckwheat, sesame seed, mustard seed, sunflower seed, and poppy seed; current)**  **Children**  NS (≥2 vs 1)  **Adults**  NS (≥2 vs 1)  **Positive sIgE (≥0.35 kUA/l) to ≥1 of 6 selected inhalant allergens (birch, mugwort, grass, and parietaria pollen, HDM, and cat; current)**  NS (≥2 vs 1)  **Children**  NS (≥2 vs 1)  **Adults**  NS (≥2 vs 1) |
| 18 | **Matricardi 1998**,^54^ IT | Case-control | 1991-1996 | Male candidates for enrollment in the air force | 11,371 | 19.7 ± 1.49 | *Birth order, sibship size*  Questionnaire | Clinical assessment | **Positive sIgE (≥0.35 kU/l) to ≥1 of HDM, cat, grass, and parietaria pollen (current)**  *Birth order*  ↓ (≥2 vs 1)  *Sibship size*  ↓ (≥3 vs 1) |
| 19 | **Mommers 2004**,^59^ DE, NL | Nested case-control | 1995, 1997 | **Cases**  Self-reported respiratory symptoms  **Controls**  General population | 638 | 7-8 | *Birth order*  Questionnaire | Clinical assessment | **Positive sIgE (≥0.35 kU/l) to ≥1 of HDM, mold, egg, milk, cat, dog, and grass mixture (current)**  ↓ (2-3 vs 1)  NS (≥4 vs 1) |
| 20 | **Moncayo 2010**,^60^ EC | Cross-sectional | 2005-2007 | General population | 3,858 | 6-16 | *Birth order*  Questionnaire | Clinical assessment | **Positive SPT to ≥1 of HDM, cockroach, cat, dog, and grass and mold mixture (current)**  NS (≥4 vs 1-3) |
| 21 | **Newson 2014**,^63^ BE, DE, DK, FI, GB, IT, MK, NL, PL, PT, SE | Case-control | 2008-2010 | General population | 3,451 | 15-77 | *Birth order*  Questionnaire | Clinical assessment | **Positive SPT to ≥1 of HDM, dog, cat, timothy, birch, olive, mold, and grass mixture (current)**  NS (≥2 vs 1) |
| 22 | **Nicolaou 2008**,^64^ GB | Cohort | 1995-2003 | General population | 922 | ~5 | *Birth order*  Questionnaire | Clinical assessment | **Positive SPT to ≥1 of HDM, cat, dog, milk, egg, and grass and mold mixture (current)**  NS (≥2 vs 1) |
| 23 | **Nowak 1996**,^65^ DE | Cross-sectional | 1990-1992 | General population | 6,428 | 20-44 | *Sibship size*  Questionnaire | Clinical assessment | **Positive SPT to ≥1 of grass, birch, cat, HDM, and mold (current)**  ↓ (≥4 vs 1) |
| 24 | **Nystad 1998**,^66^ NO | Cross-sectional | 1994 | General population | 502 | 7-16 | *Sibship size*  Questionnaire | Clinical assessment | **Positive SPT to ≥1 of egg white, milk, HDM, dog, cat, birch, timothy, and mugwort (current)**  NS (N/A) |
| 25 | **Ogbuanu 2010**,^67^ GB | Cohort | 1989-2008 | General population | 1,456 | 4-18 | *Birth order*  Questionnaire | Clinical assessment | **Detectable sIgE to ≥1 of HDM, cat, dog, horse, timothy, *cladosporium*, silver birch, olive, mugwort, and nettle (current)**  NS (≥2 vs 1)  **Positive SPT to ≥1 of 14 common food and aeroallergens (current)**  NS (≥2 vs 1) |
| 26 | **Özmert 2009**,^70^ TR | Cross-sectional | 2004 | General population | 109 | 2-4 | *Birth order*  Questionnaire | Clinical assessment | **Positive SPT to ≥1 of HDM, mold, cat, dog, milk, egg, peanut, wheat, soybean, and weeds, grass, and tree mixture (current)**  NS (≥2 vs 1) |
| 27 | **Pekkanen 2001**,^74^ FI | Cohort | 1966-1997 | General population | 5,192 | ~31 | *Birth order*  Questionnaire | Clinical assessment | **Positive SPT to ≥1 of HDM, cat, birch, and timothy (current)**  ↓ (≥2 vs 1) |
| 28 | **Perzanowski 2008**,^75^ US | Cross-sectional | N/A | Low-income population with high risk of asthma | 1,005 | ~4 | *Birth order*  Questionnaire | Clinical assessment | **Positive sIgE (≥0.35 IU/ml) to ≥1 of HDM, cockroach, mouse urinary proteins, and cat (current)**  NS (N/A) |
| 29 | **Ponsonby 2003**,^78^ AU | Cohort | 1988-1989, 1997 | General population | 498 | ~8 | *Sibship size*  Questionnaire | Clinical assessment | **Positive SPT to ≥1 of rye, HDM, mold, cat, and dog (current)**  ↓ (≥4 vs 1) |
| 30 | **Raukas-Kivioja 2006**,^81^ EE | Cross-sectional | 1997-2000 | General population | 1,346 | 17-69 | *Sibship size*  Questionnaire | Clinical assessment | **Positive SPT to ≥1 of HDM, cat, dog, cow, horse, birch, timothy, mugwort, mold miexture, latex, and cockroach (current)**  NS (≥2 vs 1) |
| 31 | **Rönmark 2009**,^82^ SE | Cohort | 1996, 2006 | General population | 3,848 | 7-8 | *Sibship size*  Questionnaire | Clinical assessment | **Positive SPT to ≥1 of birch, timothy, mugwort, dog, cat, horse, HDM, and mold mixture (current)**  **1996 cohort**  ↓ (≥2 vs 1)  **2006 cohort**  NS (≥2 vs 1) |
| 32 | **Ronmark 2017**,^84^ SE | Cohort | 1996-2007 | General population | 1,516 | 7-19 | *Birth order*  Questionnaire | Clinical assessment | **Positive SPT to ≥1 of birch, timothy, mugwort, dog, cat, horse, HDM, and mold mixture (current)**  NS (no. of older siblings) |
| 33 | **Schäfer 2020**,^88^ AU | Case-cohort | 2000-2017 | General population | 66,869 | 3.5 (1.7-7.3) | *Birth order*  Medical records | Clinical assessment | **Positive SPT to ≥1 of a palette of "standard" commercial food and aeroallergen extracts (current)**  ↓ (no. of older siblings) |
| 34 | **Sears 1996**,^89^ NZ | Cohort | 1972-1990 | General population | 1,037 | ~13 | *Birth order*  Questionnaire | Clinical assessment | **Positive SPT to ≥1 of HDM, grass, cat, dog, horse, kapok, wool, and mold mixture (current)**  NS (≥2 vs 1) |
| 35 | **Sozańska 2015**,^93^ PL | Cross-sectional | 2003, 2012 | General population | 3,340 | 5-99 | *Birth order, sibship size*  Questionnaire | Clinical assessment | **Positive SPT to ≥1 of HDM, cat, fur, and tree and grass mixture (current)**  **Village population, 2003**  ↓ (≥3 vs 1)  **Village population, 2012**  NS (≥2 vs 1)  **City population, 2003**  NS (≥2 vs 1)  **City population, 2012**  NS (≥2 vs 1) |
| 36 | **Stemeseder 2017**,^94^ AT | Cross-sectional | 2013-2014 | General population | 501 | 12-21 | *Sibship size*  Questionnaire | Clinical assessment | **sIgE sensitization measured with ImmunoCAP ISAC® and 112 single components (current)**  NS (≥2 vs 1) |
| 37 | **Strachan 1996**,^96^ GB | Cross-sectional | 1975-1991 | General population | 11,765 | 11-16 | *Birth order, sibship size*  Questionnaire | Clinical assessment | **Positive SPT to grass mixture (current)**  *Birth order*  ↓ (≥2 vs 1)  *Sibship size*  NS (≥2 vs 1) |
| 38 | **Strachan 1997**,^97^ GB | Cross-sectional | 1992-1993 | General population | 1,369 | 34-35 | *Birth order*  Interview | Clinical assessment | **Positive SPT to ≥1 of HDM, cat, and grass mixture (current)**  ↓ (≥2 vs 1) |
| 39 | **Svanes 1999**,^101^ AU, BE, CH, DE, EE, ES, FR, GB, IE, IS, IT, NL, NO, NZ, SE, US | Cross-sectional | 1990-1994 | General population | 13,932 | 20-44 | *Birth order, sibship size*  Questionnaire | Clinical assessment | **Positive sIgE (>0.35 kU/l) to ≥1 of timothy, HDM, cat, and mold (current)**  *Birth order*  ↓ (no. of older siblings)  *Sibship size*  ↓ (≥5 vs 1) |
| 40 | **Turner 2005**,^105^ AU | Cohort | 1987-2001 | General population | 182 | ~11 | *Birth order*  N/A | Clinical assessment | **Positive SPT to ≥1 of milk, egg white, rye, and HDM (current)**  **1-month-olds**  NS (≥2 vs 1)  **6-month-olds**  NS (≥2 vs 1)  **1-year-olds**  ↑ (≥2 vs 1)  **Positive SPT to ≥1 of milk, egg white, rye, HDM, cat, dog, and mold mixture (current)**  **6-year-olds**  NS (≥2 vs 1)  **11-year-olds**  NS (≥2 vs 1) |
| 41 | **Von Linstow 2002**,^108^ DK | Cross-sectional | 1998 | General population | 525 | 18-29 | *Birth order, sibship size*  Questionnaire | Clinical assessment | **Positive SPT to grass (current)**  *Birth order*  ↓ (≥2 vs 1)  *Sibship size*  NS (≥2 vs 1) |
| 42 | **Von Mutius 1994**,^109^ DE | Cross-sectional | 1989-1992 | General population | 7,653 | 9-11 | *Sibship size*  Questionnaire | Clinical assessment | **Positive SPT to ≥1 of HDM, grass, birch, hazel, cat, and dog (current)**  ↓ (≥6 vs 1) |
| 43 | **Yamazaki 2015**,^111^ JP | Cross-sectional | 2005 | General population | 8,815 | 6-9 | *Birth order, sibship size*  Questionnaire | Clinical assessment | **Positive sIgE (≥0.35 kU/l) to HDM (current)**  NS (≥2 vs 1)  **Positive sIgE (≥0.35 kU/l) to Japanese cedar pollen (current)**  ↓ (≥2 vs 1) |
| 44 | **Zekveld 2006**,^112^ GR | Cross-sectional | 2001 | Rural population | 797 | 9-14 | *Birth order*  Questionnaire | Clinical assessment | **Positive SPT to ≥1 of olive, cat, HDM, goat epithelium, mold, cockroach, poultry, and grass mixture (current)**  ↓ (2-3 vs 1)  NS (≥4 vs 1) |

**Column explanations. ^a^** The highest number of subjects in relevant analyses, or – when such numbers were not defined – the number of subjects included in the study. If no explicit number is provided, it is approximated by the largest identified number in tables or the article text and denoted as ~number. ^b^ Reported in years as either: (a) mean ± standard deviation; (b) minimum age-maximum age; (c) median age (interquartile range of age); or (d) ~approximate age. ^c^ Black bold titles indicate specific outcomes, blue bold titles indicate specific subgroups of subjects, and italic titles indicate analyses of specific exposure. For each analysis, the results are described as (a) NS (non-significant 95% confidence interval); (b) ↑ (significantly increased risk of outcome); or (c) ↓ (significantly decreased risk of outcome). In parenthesis, the sibship size or birth order from which the results are significant is indicated. **Country codes.** AT: Austria. AU: Australia. BE: Belgium. BY: Belarus. CH: Switzerland. DE: Germany. DK: Denmark. EC: Ecuador. EE: Estonia. ES: Spain. FI: Finland. FR: France. GB: United Kingdom of Great Britain and Northern Ireland. GR: Greece. IE: Ireland. IS: Iceland. IT: Italy. JP: Japan. KR: Korea, Republic of. LT: Lithuania. MK: North Macedonia. NL: Netherlands. NO: Norway. NZ: New Zealand. PL: Poland. PT: Portugal. SE: Sweden. TR: Turkey. US: United States of America. **Other abbreviations.** HDM: house dust mite. N/A: not applicable/not available. NS: non-significant. RAST: radioallergosorbent test. sIgE: allergen-specific immunoglobulin E. SPT: skin prick test. tIgE: total immunoglobulin E.

## Table E6. Publication bias

| **A. Atopic dermatitis (current) by birth order** | | | | | |
| --- | --- | --- | --- | --- | --- |
| **Kendall's tau (Begg and Mazumdar’s test)** | ***p* (Begg and Mazumdar’s test)** | **z-value (Egger's test)** | ***p* (Egger's test)** | **Missing (trim-and-fill)** | **Side (trim-and-fill)** |
| -0.16789 | 0.058182 | -2.04038 | 0.04 | 0 | right |
| **B. Atopic dermatitis (current) by sibship size** | | | | | |
| **Kendall's tau (Begg and Mazumdar’s test)** | ***p* (Begg and Mazumdar’s test)** | **z-value (Egger's test)** | ***p* (Egger's test)** | **Missing (trim-and-fill)** | **Side (trim-and-fill)** |
| 0.172932 | 0.132497 | 0.157423 | 0.87 | 0 | left |
| **C. Atopic dermatitis (ever) by birth order** | | | | | |
| **Kendall's tau (Begg and Mazumdar’s test)** | ***p* (Begg and Mazumdar’s test)** | **z-value (Egger's test)** | ***p* (Egger's test)** | **Missing (trim-and-fill)** | **Side (trim-and-fill)** |
| -0.2127 | 0.069833 | -2.02575 | 0.04 | 7 | Right |
| **D. Allergic sensitization (SPT) by birth order** | | | | | |
| **Kendall's tau (Begg and Mazumdar’s test)** | ***p* (Begg and Mazumdar’s test)** | **z-value (Egger's test)** | ***p* (Egger's test)** | **Missing (trim-and-fill)** | **Side (trim-and-fill)** |
| 0.022222 | 0.860484 | 1.026706 | 0.3 | 2 | left |

For each exposure-outcome pair with ≥ 10 studies (A-E), results are presented from Begg and Mazumdar’s (rank correlation) test (column 1-2), Egger’s (regression) test (column 3-4), and from the trim-and-fill function (column 5-6). Missing (trim-and-fill): the estimated number of missing studies in plots that are deemed asymmetric by the function (k0 from the *trimfill()* function). Side (trim-and-fill): indicates which side on which the missing values are located (side from the *trimfill()* function).

## Table E7. Sensitivity analysis by quality appraisal and outcome assessment method

| **A. Atopic dermatitis (current) by birth order** | | | | |
| --- | --- | --- | --- | --- |
| **Selection** | **No. of studies** | **Risk ratio (95% CI)** | ***I²*** | **τ²** |
| All studies | 22 | 0.98 (0.92-1.05) | 64.69 | 0.01 |
| Moderate and strong quality | 17 | 1 (0.92-1.08) | 69.75 | 0.01 |
| **B. Atopic dermatitis (current) by sibship size** | | | | |
| **Selection** | **No. of studies** | **Risk ratio (95% CI)** | ***I²*** | **τ²** |
| All studies | 14 | 0.9 (0.83-0.98) | 69.75 | 0.01 |
| Moderate and strong quality | 14 | 0.9 (0.83-0.98) | 69.75 | 0.01 |
| Physician/clinical outcome assessment | 4 | 0.84 (0.68-1.05) | 10.06 | 0 |
| **C. Atopic dermatitis (ever) by birth order** | | | | |
| **Selection** | **No. of studies** | **Risk ratio (95% CI)** | ***I²*** | **τ²** |
| All studies | 16 | 0.91 (0.84-0.98) | 76.87 | 0.01 |
| Moderate and strong quality | 12 | 0.91 (0.83-1.01) | 81.14 | 0.01 |
| **D. Atopic dermatitis (ever) by sibship size** | | | | |
| **Selection** | **No. of studies** | **Risk ratio (95% CI)** | ***I²*** | **τ²** |
| All studies | 7 | 0.92 (0.86-0.97) | 67.22 | 0 |
| Moderate and strong quality | 7 | 0.92 (0.86-0.97) | 67.22 | 0 |
| **E. Food allergy (current) by birth order** | | | | |
| **Selection** | **No. of studies** | **Risk ratio (95% CI)** | ***I²*** | **τ²** |
| All studies | 6 | 0.77 (0.66-0.9) | 7.37 | 0 |
| Moderate and strong quality | 5 | 0.77 (0.64-0.92) | 24.98 | 0.01 |
| **F. Allergic sensitization (sIgE) by birth order** | | | | |
| **Selection** | **No. of studies** | **Risk ratio (95% CI)** | ***I²*** | **τ²** |
| All studies | 7 | 0.89 (0.79-1.01) | 51.95 | 0.01 |
| Moderate and strong quality | 6 | 0.9 (0.79-1.03) | 59.18 | 0.01 |
| **G. Allergic sensitization (sIgE) by sibship size** | | | | |
| **Selection** | **No. of studies** | **Risk ratio (95% CI)** | ***I²*** | **τ²** |
| All studies | 5 | 0.92 (0.79-1.08) | 67.03 | 0.02 |
| Moderate and strong quality | 5 | 0.92 (0.79-1.08) | 67.03 | 0.02 |
| Physician/clinical outcome assessment | 5 | 0.92 (0.79-1.08) | 67.03 | 0.02 |
| **H. Allergic sensitization (SPT) by birth order** | | | | |
| **Selection** | **No. of studies** | **Risk ratio (95% CI)** | ***I²*** | **τ²** |
| All studies | 12 | 0.86 (0.77-0.97) | 67.05 | 0.02 |
| Moderate and strong quality | 11 | 0.85 (0.75-0.96) | 67.59 | 0.02 |
| **I. Allergic sensitization (SPT) by sibship size** | | | | |
| **Selection** | **No. of studies** | **Risk ratio (95% CI)** | ***I²*** | **τ²** |
| All studies | 8 | 0.88 (0.83-0.92) | 39.11 | 0.01 |
| Moderate and strong quality | 8 | 0.88 (0.83-0.92) | 39.11 | 0.01 |
| Physician/clinical outcome assessment | 8 | 0.88 (0.83-0.92) | 39.11 | 0.01 |

For each exposure-outcome pair where there were ≥ 2 studies to perform at least one sensitivity analysis, results are presented on the number of studies, risk ratio with 95% confidence interval, as well as *I^2^* and τ². Moderate and strong quality: overall rating of study with the Effective Public Health Practice Project (EPHPP) tool. Clinically confirmed outcome: outcome assessed by medical records or clinical examination.

## Table E8. Sensitivity analysis by rho value used in the meta-analyses

| **A. Atopic dermatitis (current) by birth order** | | | | | | |
| --- | --- | --- | --- | --- | --- | --- |
|  | **Rho = 0** | **Rho = 0.2** | **Rho = 0.4** | **Rho = 0.6** | **Rho = 0.8** | **Rho = 1** |
| Risk ratio (95% CI) | 0.98 (0.92-1.05) | 0.98 (0.92-1.05) | 0.98 (0.92-1.05) | 0.98 (0.92-1.05) | 0.98 (0.92-1.05) | 0.98 (0.92-1.05) |
| τ^2^ | 0.0109 | 0.0109 | 0.0109 | 0.0109 | 0.0109 | 0.0110 |
| **B. Atopic dermatitis (current) by sibship size** | | | | | | |
|  | **Rho = 0** | **Rho = 0.2** | **Rho = 0.4** | **Rho = 0.6** | **Rho = 0.8** | **Rho = 1** |
| Risk ratio (95% CI) | 0.90 (0.83-0.98) | 0.90 (0.83-0.98) | 0.90 (0.83-0.98) | 0.90 (0.83-0.98) | 0.90 (0.83-0.98) | 0.90 (0.83-0.98) |
| τ^2^ | 0.0134 | 0.0135 | 0.0136 | 0.0136 | 0.0137 | 0.0138 |
| **C. Atopic dermatitis (ever) by birth order** | | | | | | |
|  | **Rho = 0** | **Rho = 0.2** | **Rho = 0.4** | **Rho = 0.6** | **Rho = 0.8** | **Rho = 1** |
| Risk ratio (95% CI) | 0.91 (0.84-0.98) | 0.91 (0.84-0.98) | 0.91 (0.84-0.98) | 0.91 (0.84-0.98) | 0.91 (0.84-0.98) | 0.91 (0.84-0.98) |
| τ^2^ | 0.0100 | 0.0101 | 0.0101 | 0.0101 | 0.0101 | 0.0101 |
| **D. Atopic dermatitis (ever) by sibship size** | | | | | | |
|  | **Rho = 0** | **Rho = 0.2** | **Rho = 0.4** | **Rho = 0.6** | **Rho = 0.8** | **Rho = 1** |
| Risk ratio (95% CI) | 0.92 (0.87-0.97) | 0.92 (0.87-0.97) | 0.92 (0.87-0.97) | 0.92 (0.86-0.97) | 0.92 (0.86-0.97) | 0.92 (0.86-0.97) |
| τ^2^ | 0.00376 | 0.0038 | 0.00385 | 0.0039 | 0.00395 | 0.0040 |
| **E. Food allergy (current) by birth order** | | | | | | |
|  | **Rho = 0** | **Rho = 0.2** | **Rho = 0.4** | **Rho = 0.6** | **Rho = 0.8** | **Rho = 1** |
| Risk ratio (95% CI) | 0.77 (0.66-0.90) | 0.77 (0.66-0.90) | 0.77 (0.66-0.90) | 0.77 (0.66-0.90) | 0.77 (0.66-0.90) | 0.77 (0.66-0.90) |
| τ^2^ | 0.000514 | 0.00111 | 0.0017 | 0.00229 | 0.00288 | 0.00347 |
| **F. Allergic sensitization (sIgE) by birth order** | | | | | | |
|  | **Rho = 0** | **Rho = 0.2** | **Rho = 0.4** | **Rho = 0.6** | **Rho = 0.8** | **Rho = 1** |
| Risk ratio (95% CI) | 0.89 (0.79-1.01) | 0.89 (0.79-1.01) | 0.89 (0.79-1.01) | 0.89 (0.79-1.01) | 0.89 (0.79-1.01) | 0.89 (0.79-1.01) |
| τ^2^ | 0.00949 | 0.00963 | 0.00977 | 0.00991 | 0.0100 | 0.0102 |
| **G. Allergic sensitization (sIgE) by sibship size** | | | | | | |
|  | **Rho = 0** | **Rho = 0.2** | **Rho = 0.4** | **Rho = 0.6** | **Rho = 0.8** | **Rho = 1** |
| Risk ratio (95% CI) | 0.92 (0.79-1.08) | 0.92 (0.79-1.08) | 0.92 (0.79-1.08) | 0.92 (0.79-1.08) | 0.92 (0.79-1.08) | 0.92 (0.79-1.08) |
| τ^2^ | 0.0143 | 0.0146 | 0.0149 | 0.0152 | 0.0155 | 0.0157 |
| **H. Allergic sensitization (SPT) by birth order** | | | | | | |
|  | **Rho = 0** | **Rho = 0.2** | **Rho = 0.4** | **Rho = 0.6** | **Rho = 0.8** | **Rho = 1** |
| Risk ratio (95% CI) | 0.86 (0.77-0.97) | 0.86 (0.77-0.97) | 0.86 (0.77-0.97) | 0.86 (0.77-0.97) | 0.86 (0.77-0.97) | 0.86 (0.77-0.97) |
| τ^2^ | 0.0195 | 0.0195 | 0.0196 | 0.0197 | 0.0197 | 0.0198 |
| **I. Allergic sensitization (SPT) by sibship size** | | | | | | |
|  | **Rho = 0** | **Rho = 0.2** | **Rho = 0.4** | **Rho = 0.6** | **Rho = 0.8** | **Rho = 1** |
| Risk ratio (95% CI) | 0.88 (0.84-0.92) | 0.88 (0.84-0.92) | 0.88 (0.84-0.92) | 0.88 (0.84-0.92) | 0.88 (0.83-0.92) | 0.88 (0.83-0.92) |
| τ^2^ | 0.00855 | 0.00878 | 0.00901 | 0.00924 | 0.00947 | 0.0097 |

For every exposure-outcome pair, presented are results from sensitivity analysis of the meta-analysis, based on the rho parameter value. With increments of 0.2 from 0 to 1, the meta-analysis was re-run and the risk ratio with 95% confidence interval (95% CI) as well as tau-squared (τ^2^) were calculated.

# Figures

## Figure E1A. Forest plot (atopic dermatitis [current] by birth order)

**
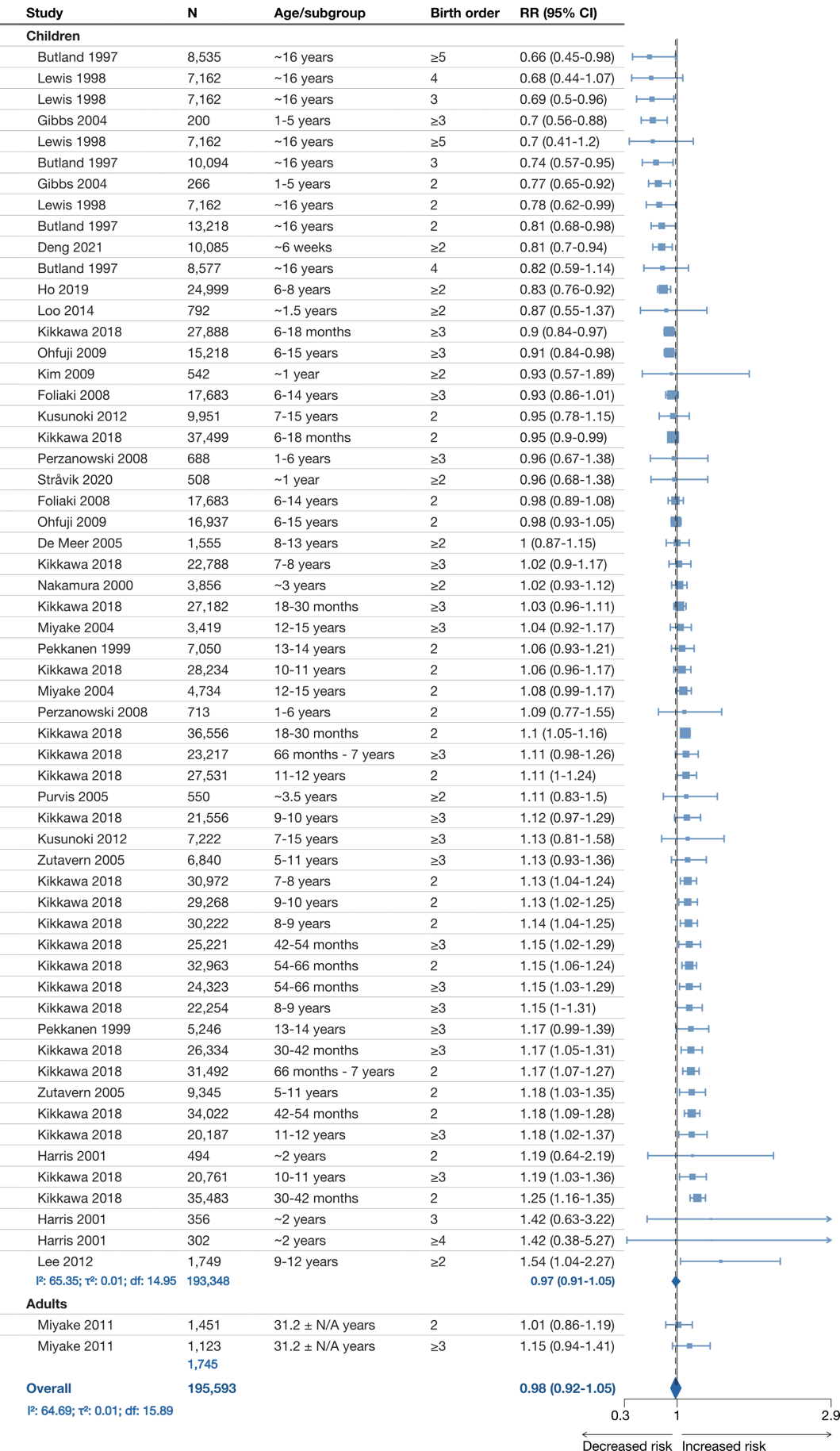
**

**Figure E1A**. Forest plot for birth order ≥ 2 vs 1 in relation to current (in last year) atopic dermatitis, divided by children (<18 years) and adults (≥18 years). **Abbrevations.** N: number of subjects (if not available, the number of subjects for the most similar exposure-outcome pair or for the whole study is stated). RR (95% CI): risk ratio (95% confidence interval).

## Figure E1B. Forest plot (atopic dermatitis [current] by sibship size)

**
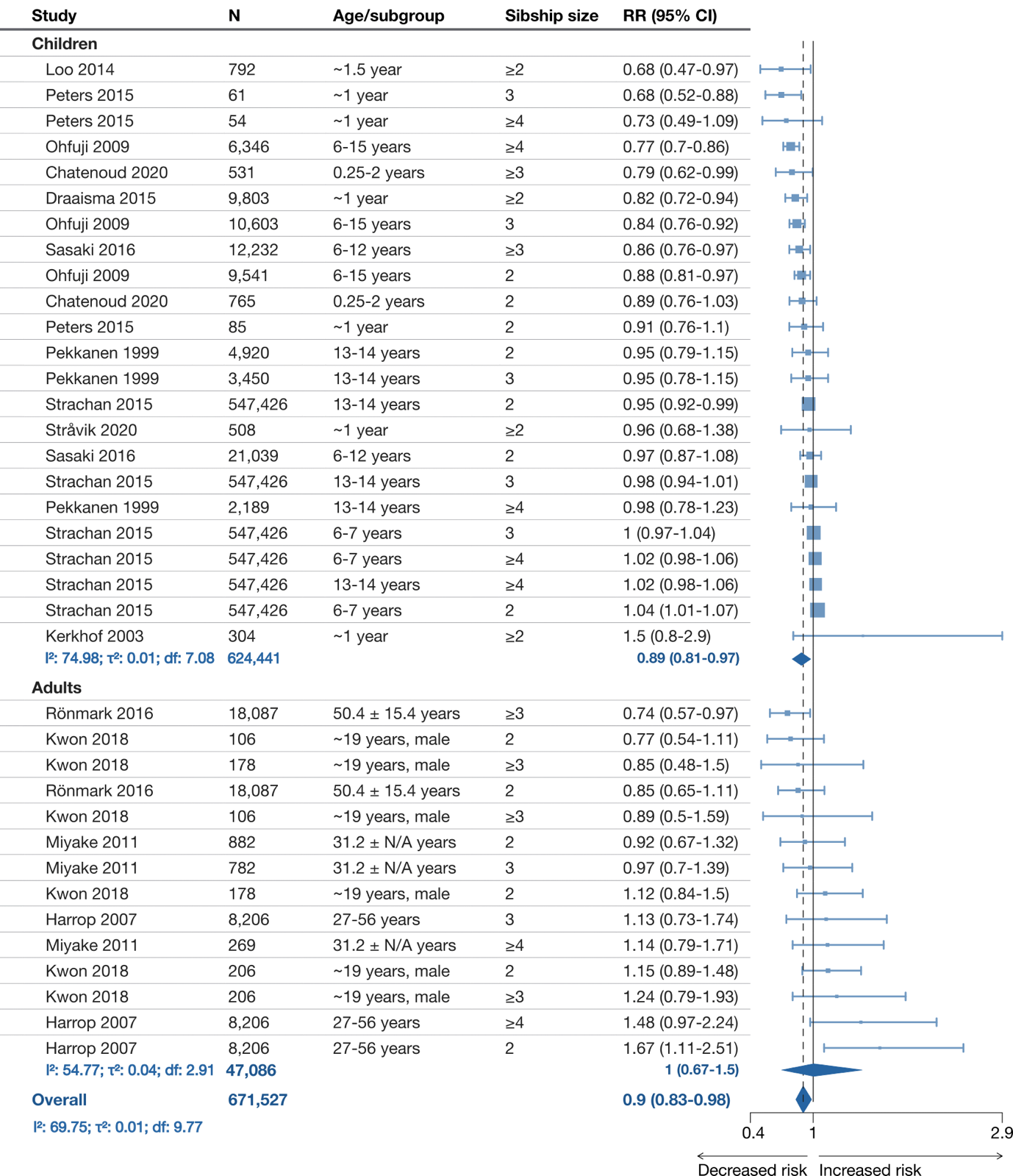
**

**Figure E1B**. Forest plot for sibship size ≥ 2 vs 1 in relation to current (in last year) atopic dermatitis, divided by children (<18 years) and adults (≥18 years). **Abbrevations.** N: number of subjects (if not available, the number of subjects for the most similar exposure-outcome pair or for the whole study is stated). RR (95% CI): risk ratio (95% confidence interval).

## Figure E2A. Forest plot (atopic dermatitis [ever] by birth order)

**
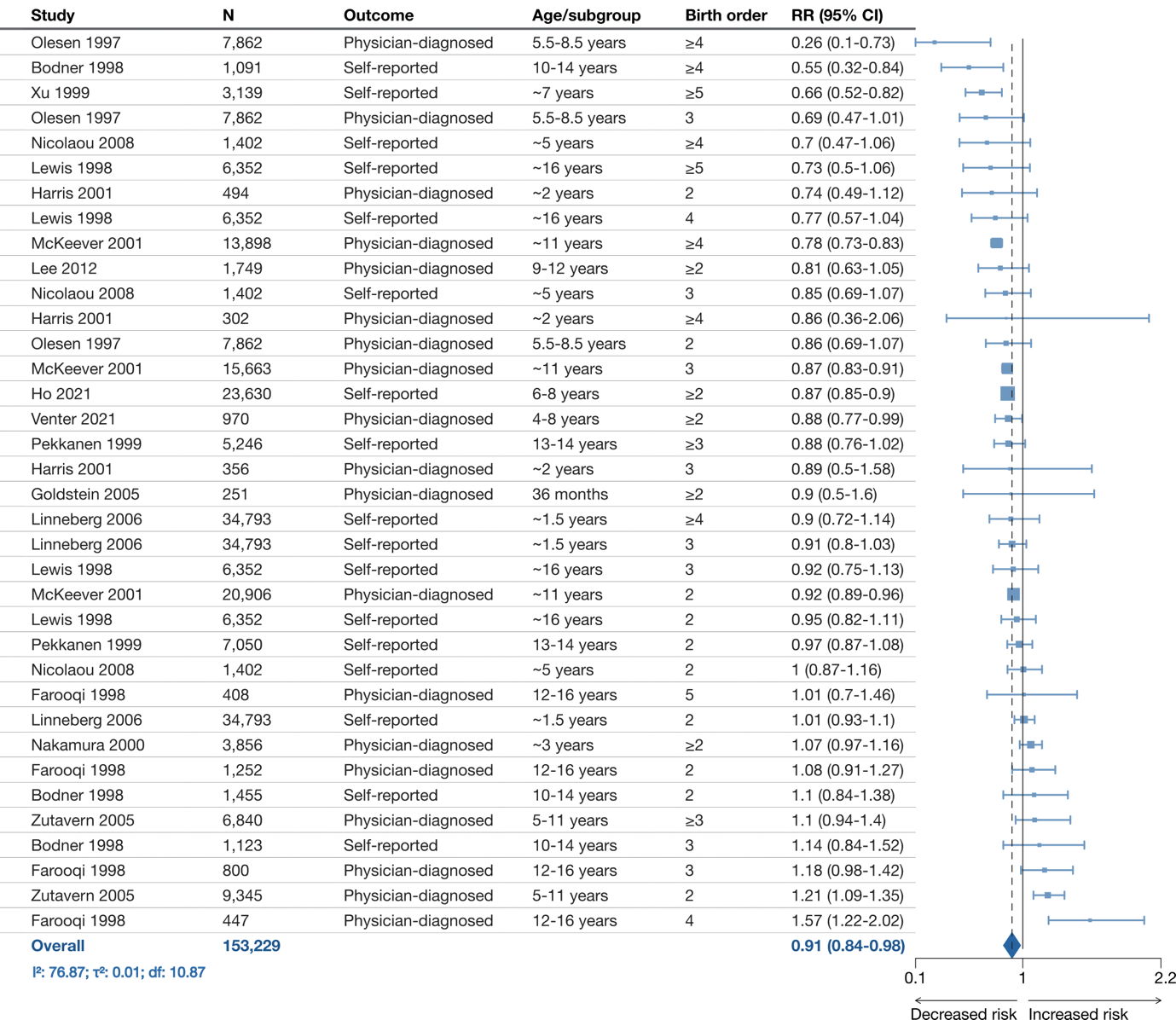
**

**Figure E2A**. Forest plot for birth order ≥ 2 vs 1 in relation to ever atopic dermatitis. **Abbrevations.** N: number of subjects (if not available, the number of subjects for the most similar exposure-outcome pair or for the whole study is stated). RR (95% CI): risk ratio (95% confidence interval).

## Figure E2B. Forest plot (atopic dermatitis [ever] by sibship size)

**
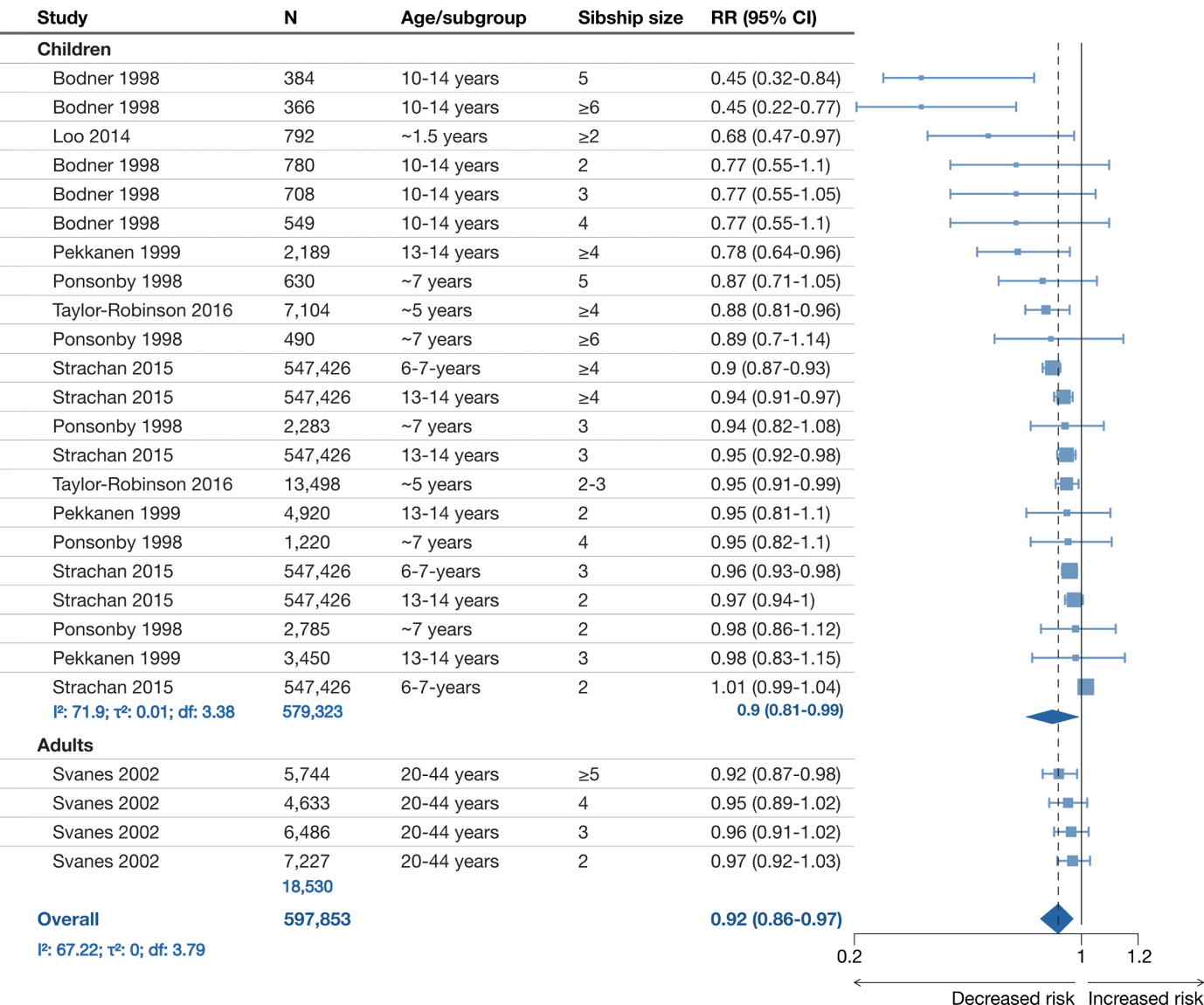
**

**Figure E2B**. Forest plot for sibship size ≥ 2 vs 1 in relation to ever atopic dermatitis, divided by children (<18 years) and adults (≥18 years). **Abbrevations.** N: number of subjects (if not available, the number of subjects for the most similar exposure-outcome pair or for the whole study is stated). RR (95% CI): risk ratio (95% confidence interval).

## Figure E3. Forest plot (food allergy [current] by birth order)

**
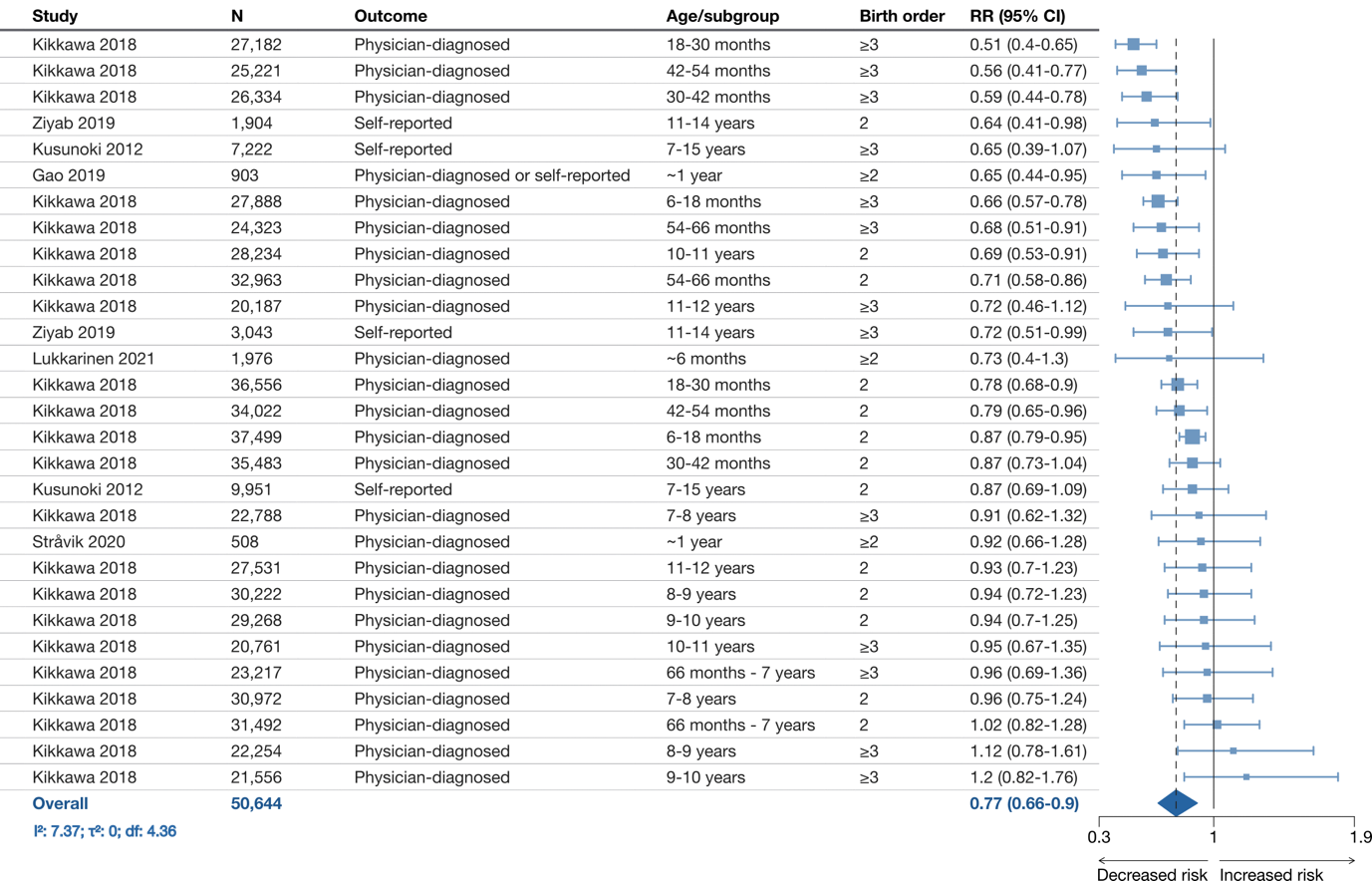
**

**Figure E3**. Forest plot for birth order ≥ 2 vs 1 in relation to any current (in last year) food allergy. **Abbrevations.** N: number of subjects (if not available, the number of subjects for the most similar exposure-outcome pair or for the whole study is stated). RR (95% CI): risk ratio (95% confidence interval).

## Figure E4A. Forest plot (allergic sensitization [allergen-specific immunoglobulin E] by birth order)

**
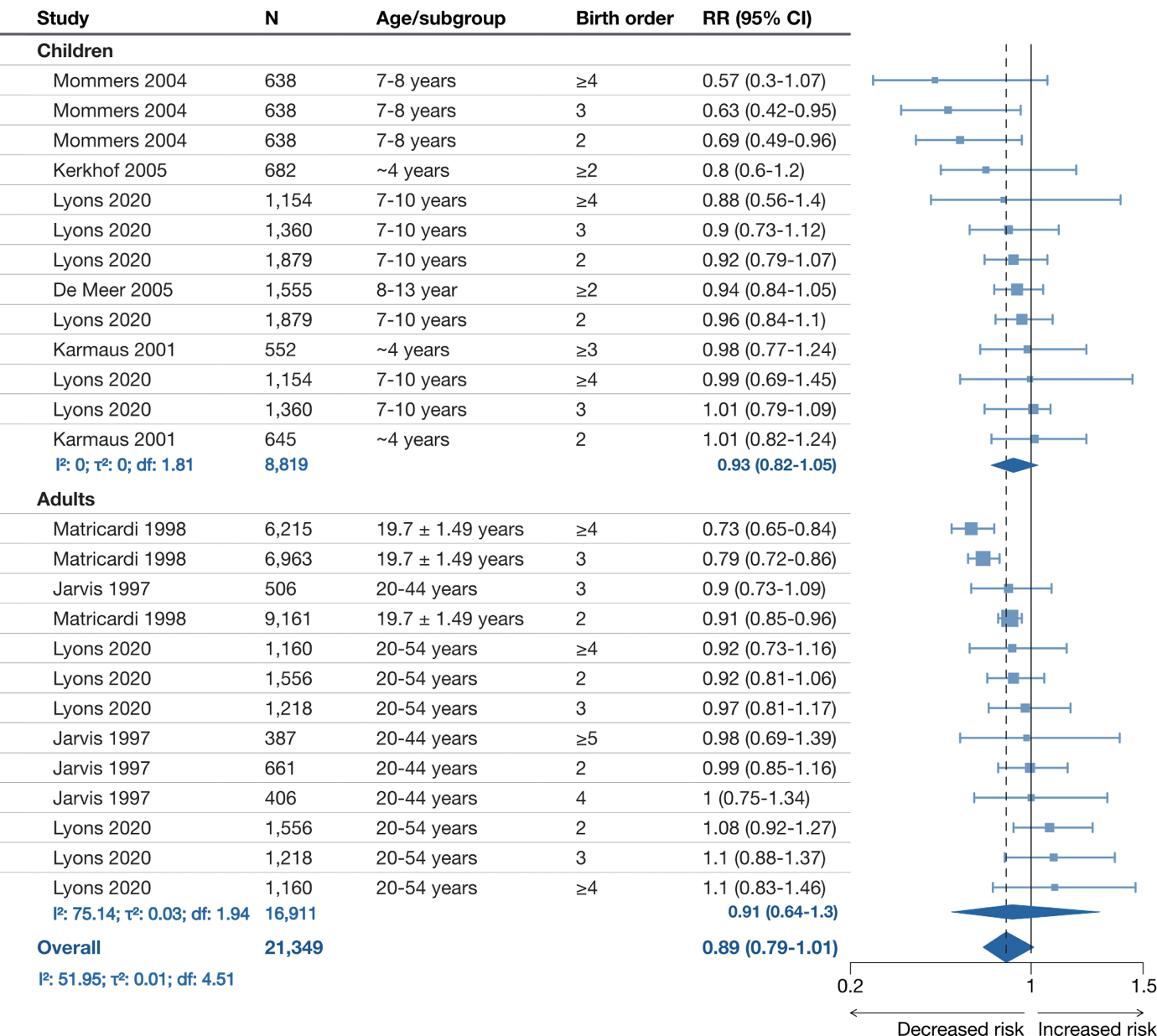
**

**Figure E4A**. Forest plot for birth order ≥ 2 vs 1 in relation to allergic sensitization assessed by measurement of allergen-specific immunoglobulin E (sIgE) levels to palettes of common food allergens and aeroallergens, divided by children (<18 years) and adults (≥18 years). **Abbrevations.** N: number of subjects (if not available, the number of subjects for the most similar exposure-outcome pair or for the whole study is stated). RR (95% CI): risk ratio (95% confidence interval).

## Figure E4B. Forest plot (allergic sensitization [allergen-specific immunoglobulin E] by sibship size)

**
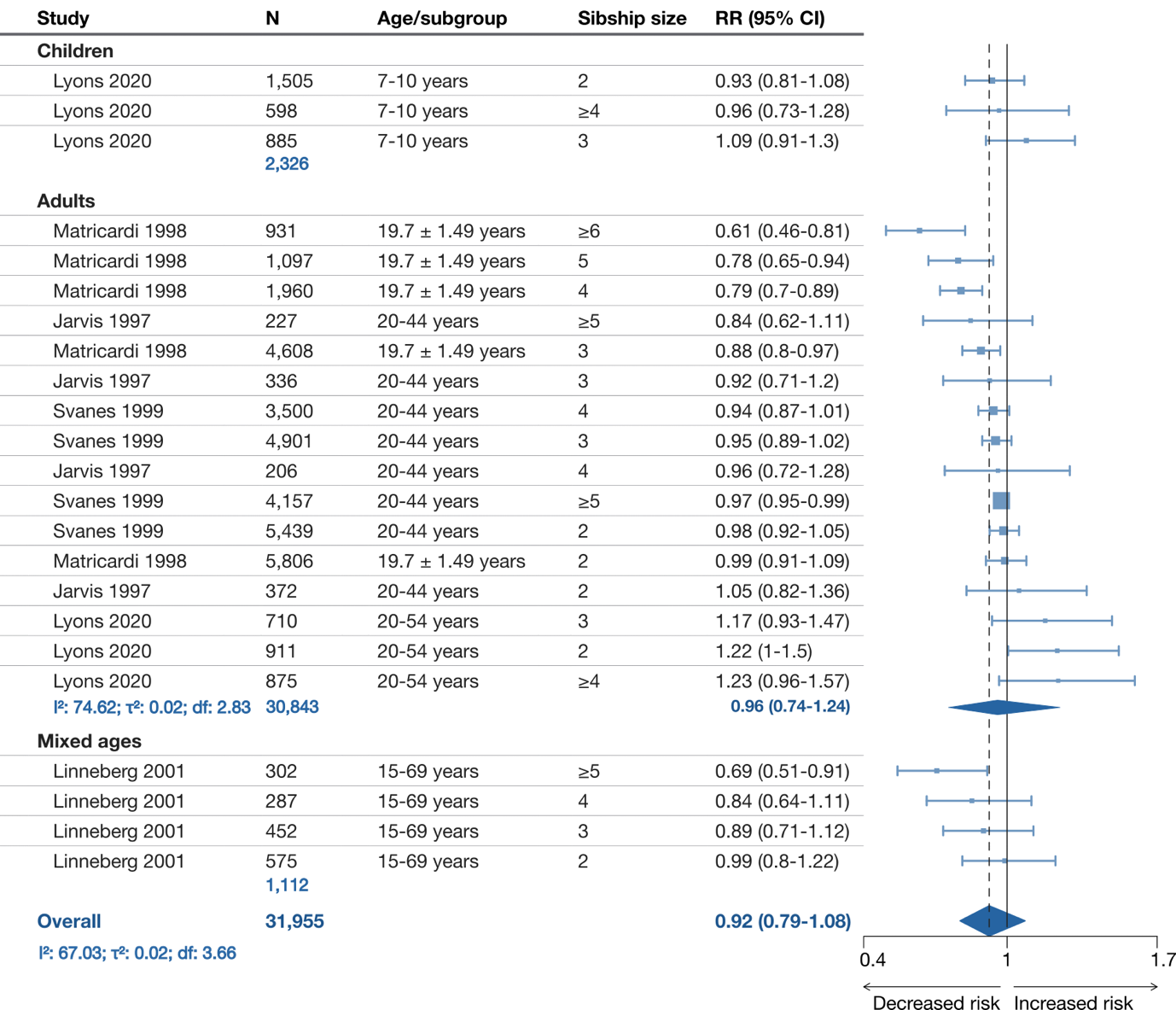
**

**Figure E4B**. Forest plot for sibship size ≥ 2 vs 1 in relation to in relation to allergic sensitization assessed by measurement of allergen-specific immunoglobulin E (sIgE) levels to palettes of common aeroallergens, divided by children (<18 years) and adults (≥18 years). **Abbrevations.** N: number of subjects (if not available, the number of subjects for the most similar exposure-outcome pair or for the whole study is stated). RR (95% CI): risk ratio (95% confidence interval).

## Figure E5A. Forest plot (allergic sensitization [skin prick test] by birth order)

**
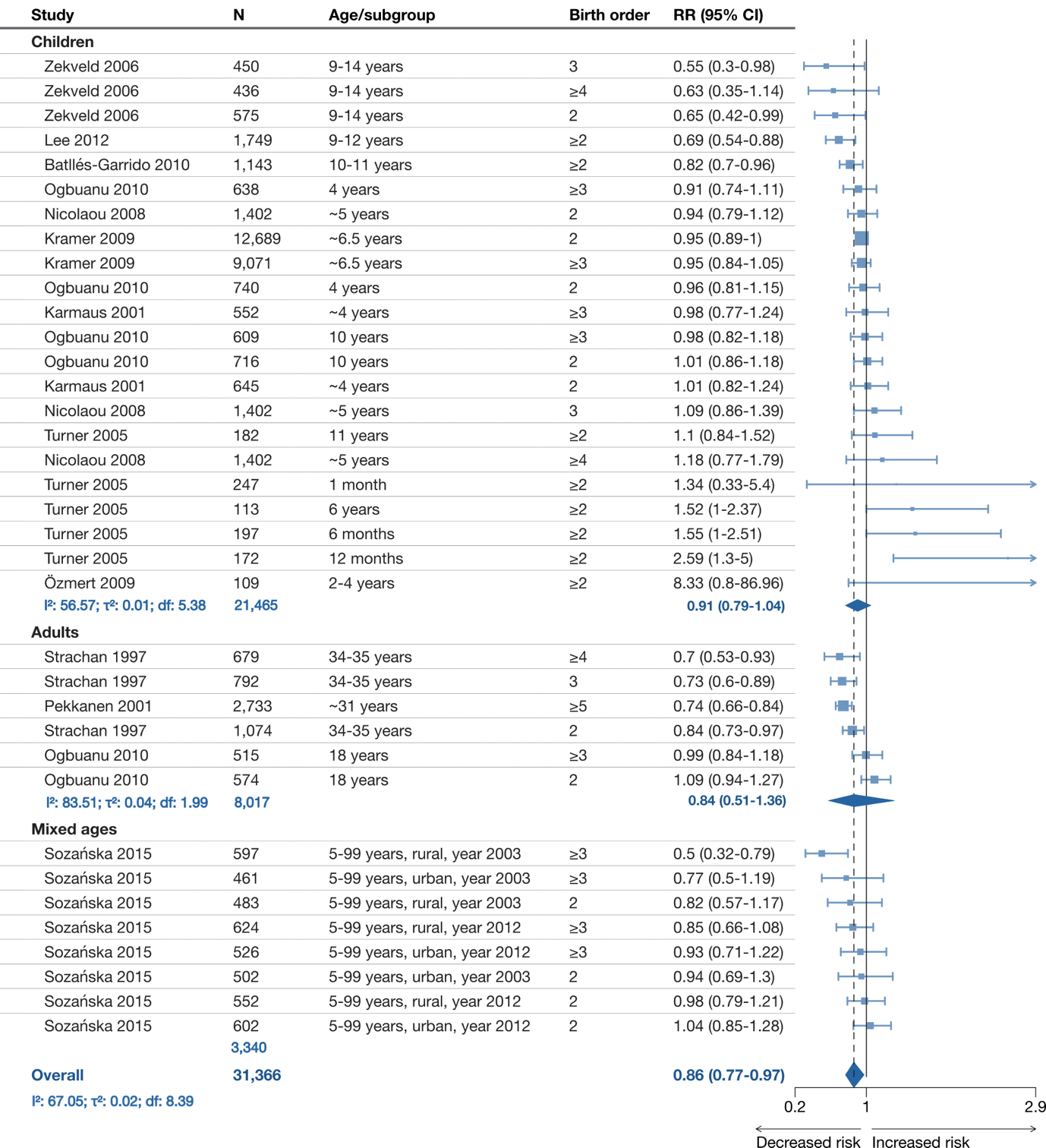
**

**Figure E5A**. Forest plot for birth order ≥ 2 vs 1 in relation to allergic sensitization assessed by skin prick tests (SPT) to common food allergens and aeroallergens, divided by children (<18 years) and adults (≥18 years). **Abbrevations.** N: number of subjects (if not available, the number of subjects for the most similar exposure-outcome pair or for the whole study is stated). RR (95% CI): risk ratio (95% confidence interval).

## Figure E5B. Forest plot (allergic sensitization [skin prick test] by sibship size)

**
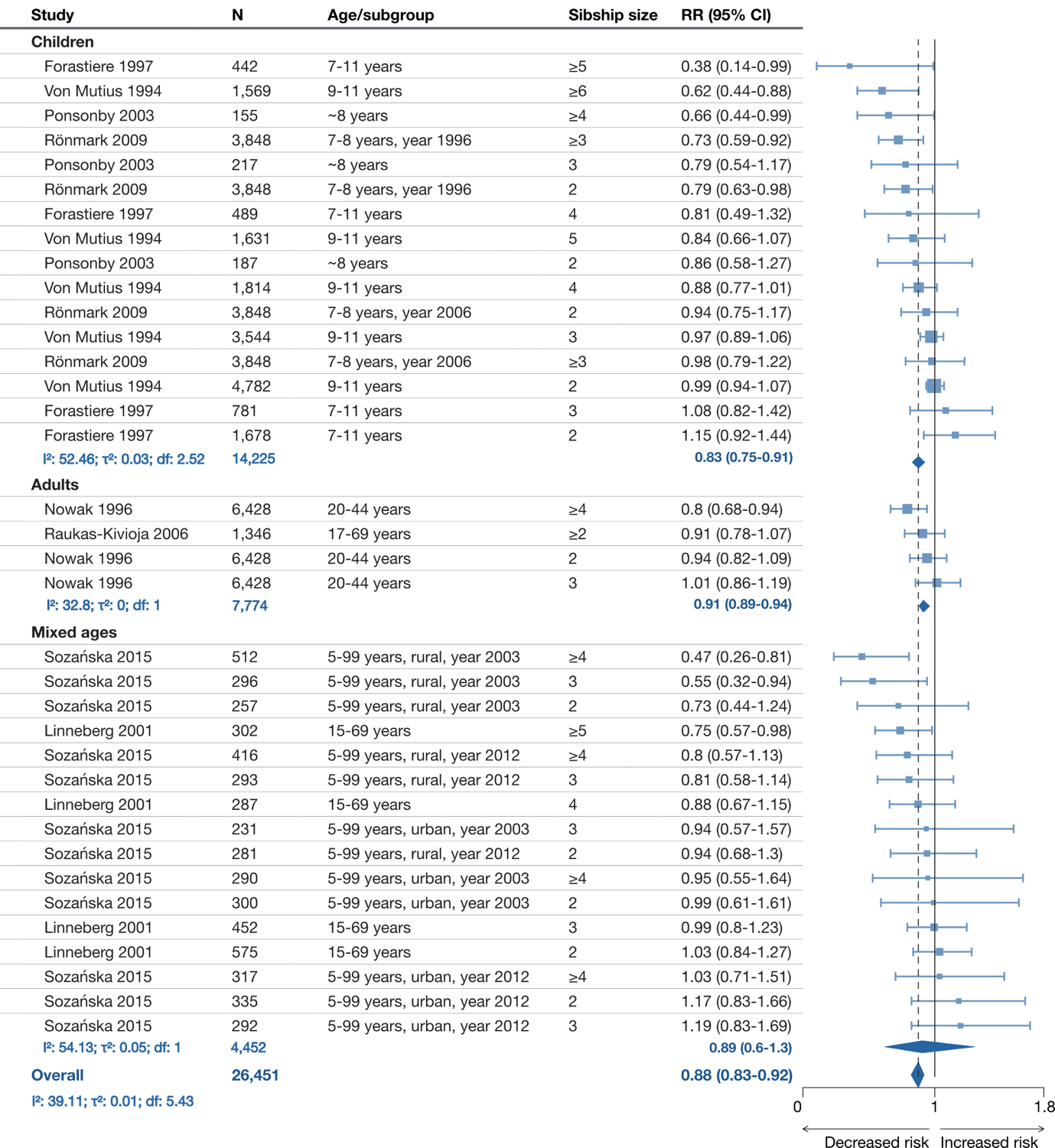
**

**Figure E5B**. Forest plot for sibship size ≥ 2 vs 1 in relation to allergic sensitization assessed by skin prick tests (SPT) to common aeroallergens, divided by children (<18 years) and adults (≥18 years). **Abbrevations.** N: number of subjects (if not available, the number of subjects for the most similar exposure-outcome pair or for the whole study is stated). RR (95% CI): risk ratio (95% confidence interval).

## Figure E6. Funnel plots (prior to trim-and-fill)

**
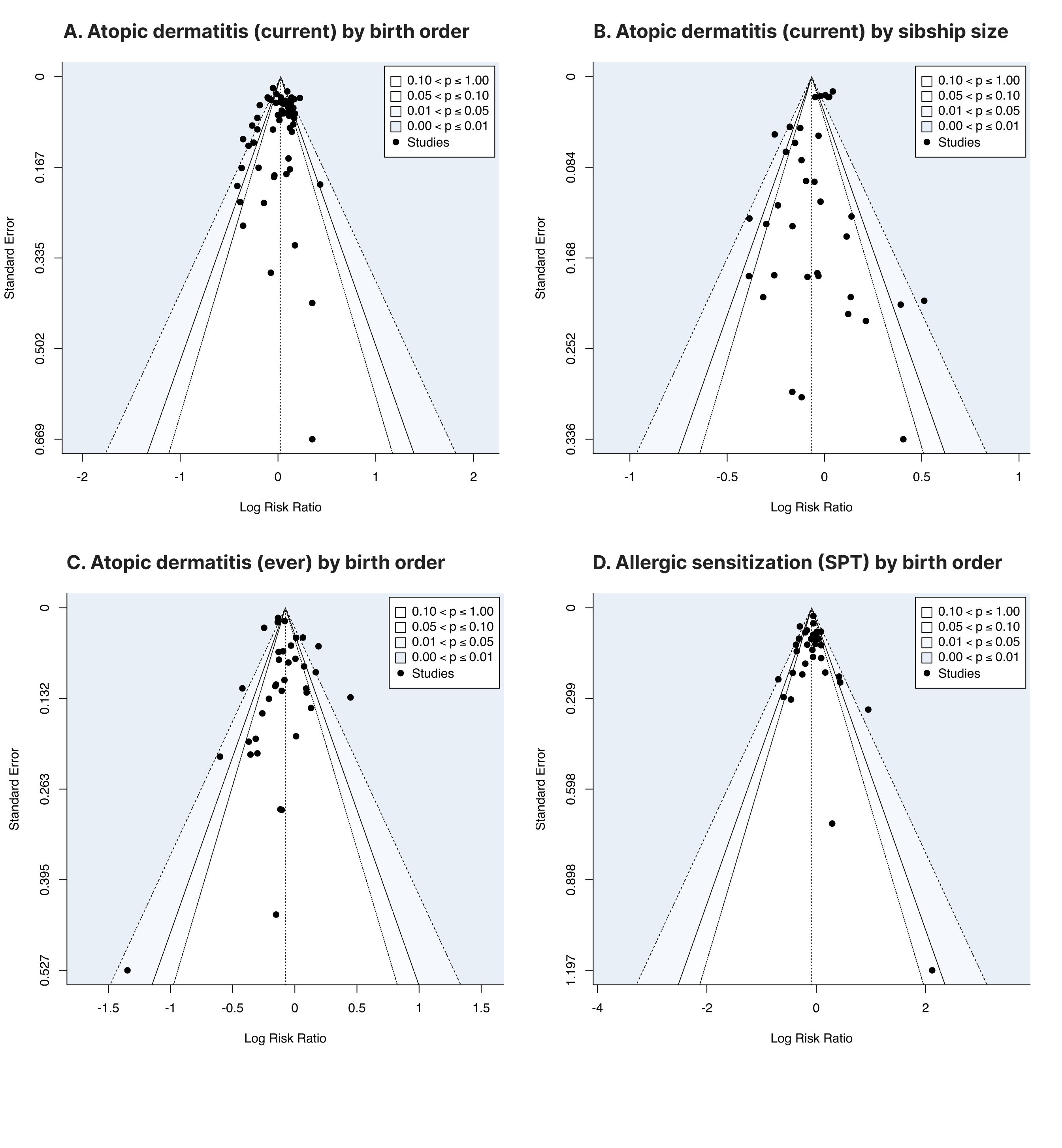
**

**Figure E4**. Funnel plots for exposure-outcome pairs with ≥10 studies.

## Figure E7. Funnel plots (after trim-and-fill)

**
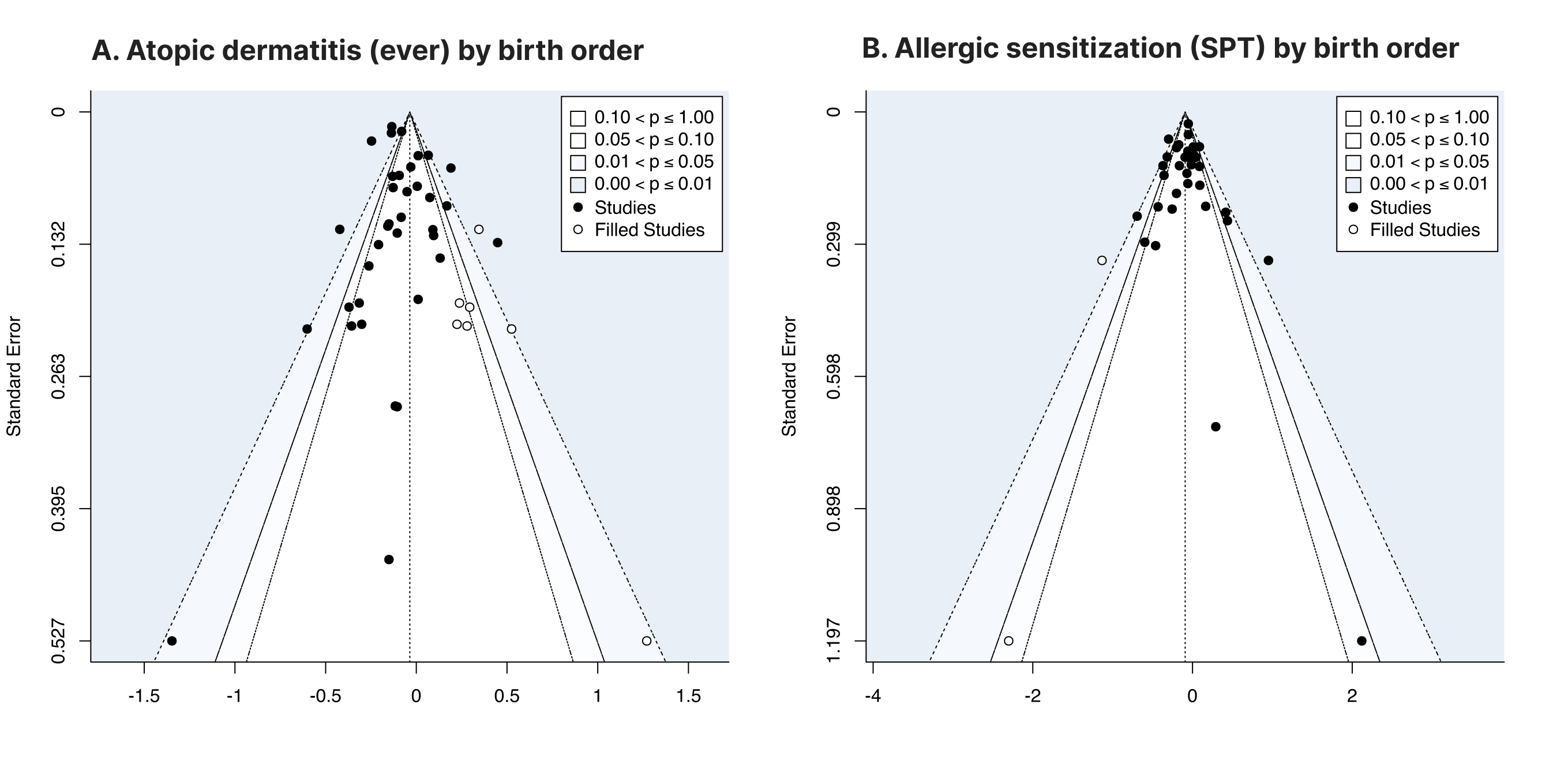
**

**Figure E5**. Funnel plots for exposure-outcome pairs with ≥ 10 studies, in which the trim-and-fill function identified asymmetry and filled ≥ 1 estimate.

# References to all included studies

1. Al-Hammadi S, Al-Maskari F, Bernsen R. Prevalence of food allergy among children in Al-Ain city, United Arab Emirates. *Int Arch Allergy Immunol*. 2010;151(4):336-42. doi:10.1159/000250442

2. Smt.Ayatollahi, H.Ghaem. Prevalence of Atopic diseases (Allergic rhinitis, Urticaria, Eczema) and its correlations in primary school children, Shiraz, Iran. شیوع بیماری های اتوپی (رنیت آلرژیک، کهیر و اگزما) و همبسته های آن در دانش آموزان دبستانی شیراز 82-1381. *J-Gorgan-Univ-Med-Sci*. 2004;6(1):29-34.

3. Batllés-Garrido J, Torres-Borrego J, Rubí-Ruiz T, et al. Prevalence and factors linked to atopy in 10-and 11-year-old children in Almería, Spain. *Allergol Immunopathol (Madr)*. Jan-Feb 2010;38(1):13-9. doi:10.1016/j.aller.2009.07.006

4. Bedolla-Barajas M, Javier Ramírez-Cervantes F, Morales-Romero J, Jesús Pérez-Molina J, Meza-López C, Delgado-Figueroa N. A rural environment does not protect against asthma or other allergic diseases amongst Mexican children. *Allergol Immunopathol (Madr)*. Jan-Feb 2018;46(1):31-38. doi:10.1016/j.aller.2017.01.010

5. Bingol A, Uygun DFK, Akdemir M, et al. Clinical phenotypes of childhood food allergies based on immune mechanisms: A multicenter study. *Allergy Asthma Proc*. May 1 2021;42(3):e86-e95. doi:10.2500/aap.2021.42.210005

6. Bodner C, Godden D, Seaton A. Family size, childhood infections and atopic diseases. The Aberdeen WHEASE Group. *Thorax*. Jan 1998;53(1):28-32. doi:10.1136/thx.53.1.28

7. Bodner C, Anderson WJ, Reid TS, Godden DJ. Childhood exposure to infection and risk of adult onset wheeze and atopy. *Thorax*. May 2000;55(5):383-7. doi:10.1136/thorax.55.5.383

8. Brooks K, Samms-Vaughan M, Karmaus W. Are oral contraceptive use and pregnancy complications risk factors for atopic disorders among offspring? *Pediatr Allergy Immunol*. Dec 2004;15(6):487-96. doi:10.1111/j.1399-3038.2004.00185.x

9. Burr ML, Merrett TG, Dunstan FD, Maguire MJ. The development of allergy in high-risk children. *Clin Exp Allergy*. Nov 1997;27(11):1247-53.

10. Butland BK, Strachan DP, Lewis S, Bynner J, Butler N, Britton J. Investigation into the increase in hay fever and eczema at age 16 observed between the 1958 and 1970 British birth cohorts. *Bmj*. Sep 20 1997;315(7110):717-21. doi:10.1136/bmj.315.7110.717

11. Celik G, Sin B, Keskin S, et al. Risk factors determining allergic airway diseases in Turkish subjects. *J Asthma*. Aug 2002;39(5):383-90. doi:10.1081/jas-120004031

12. Chatenoud L, Bertuccio P, Turati F, et al. Markers of microbial exposure lower the incidence of atopic dermatitis. <https://doi.org/10.1111/all.13990>. *Allergy*. 2020/01/01 2020;75(1):104-115. doi:<https://doi.org/10.1111/all.13990>

13. Cooper PJ, Vaca M, Rodriguez A, et al. Hygiene, atopy and wheeze-eczema-rhinitis symptoms in schoolchildren from urban and rural Ecuador. *Thorax*. Mar 2014;69(3):232-9. doi:10.1136/thoraxjnl-2013-203818

14. Cullinan P, Harris JM, Newman Taylor AJ, et al. Can early infection explain the sibling effect in adult atopy? *European Respiratory Journal*. 2003;22(6):956. doi:10.1183/09031936.03.00039102

15. Davis JB, Bulpitt CJ. Atopy and wheeze in children according to parental atopy and family size. *Thorax*. Mar 1981;36(3):185-9. doi:10.1136/thx.36.3.185

16. de Meer G, Janssen NA, Brunekreef B. Early childhood environment related to microbial exposure and the occurrence of atopic disease at school age. *Allergy*. May 2005;60(5):619-25. doi:10.1111/j.1398-9995.2005.00746.x

17. Deng L, Liu H, Wei D, et al. Incidence of Eczema in Early Infancy and the Prenatal Risk Factors - Guangzhou, Guangdong, China, 2018-2019. *China CDC Wkly*. Aug 13 2021;3(33):693-696. doi:10.46234/ccdcw2021.173

18. Draaisma E, Garcia-Marcos L, Mallol J, Solé D, Pérez-Fernández V, Brand PL. A multinational study to compare prevalence of atopic dermatitis in the first year of life. *Pediatr Allergy Immunol*. Jun 2015;26(4):359-66. doi:10.1111/pai.12388

19. Farooqi IS, Hopkin JM. Early childhood infection and atopic disorder. *Thorax*. Nov 1998;53(11):927-32. doi:10.1136/thx.53.11.927

20. Foliaki S, Annesi-Maesano I, Tuuau-Potoi N, et al. Risk factors for symptoms of childhood asthma, allergic rhinoconjunctivitis and eczema in the Pacific: an ISAAC Phase III study. *Int J Tuberc Lung Dis*. Jul 2008;12(7):799-806.

21. Forastiere F, Agabiti N, Corbo GM, et al. Socioeconomic status, number of siblings, and respiratory infections in early life as determinants of atopy in children. *Epidemiology*. Sep 1997;8(5):566-70. doi:10.1097/00001648-199709000-00015

22. Gao X, Yan Y, Zeng G, et al. Influence of prenatal and early-life exposures on food allergy and eczema in infancy: a birth cohort study. *BMC Pediatr*. Jul 17 2019;19(1):239. doi:10.1186/s12887-019-1623-3

23. Genuneit J, Strachan DP, Büchele G, et al. The combined effects of family size and farm exposure on childhood hay fever and atopy. *Pediatr Allergy Immunol*. May 2013;24(3):293-8. doi:10.1111/pai.12053

24. Gibbs S, Surridge H, Adamson R, Cohen B, Bentham G, Reading R. Atopic dermatitis and the hygiene hypothesis: a case-control study. *Int J Epidemiol*. Feb 2004;33(1):199-207. doi:10.1093/ije/dyg267

25. Goldstein IF, Perzanowski MS, Lendor C, et al. Prevalence of Allergy Symptoms and Total IgE in a New York City Cohort and Their Association with Birth Order. *International Archives of Allergy and Immunology*. 2005;137(3):249-257. doi:10.1159/000086338

26. Gupta RS, Singh AM, Walkner M, et al. Hygiene factors associated with childhood food allergy and asthma. *Allergy Asthma Proc*. Nov 2016;37(6):e140-e146. doi:10.2500/aap.2016.37.3988

27. Haileamlak A, Dagoye D, Williams H, et al. Early life risk factors for atopic dermatitis in Ethiopian children. *J Allergy Clin Immunol*. Feb 2005;115(2):370-6. doi:10.1016/j.jaci.2004.10.024

28. Harris JM, Cullinan P, Williams HC, et al. Environmental associations with eczema in early life. *Br J Dermatol*. Apr 2001;144(4):795-802. doi:10.1046/j.1365-2133.2001.04135.x

29. Harrop J, Chinn S, Verlato G, et al. Eczema, atopy and allergen exposure in adults: a population-based study. *Clin Exp Allergy*. Apr 2007;37(4):526-35. doi:10.1111/j.1365-2222.2007.02679.x

30. Ho CL, Chang LI, Wu WF. The prevalence and risk factors of atopic dermatitis in 6-8 year-old first graders in Taipei. *Pediatr Neonatol*. Apr 2019;60(2):166-171. doi:10.1016/j.pedneo.2018.05.010

31. Ho CL, Wu WF. Risk factor analysis of allergic rhinitis in 6-8 year-old children in Taipei. *PLoS One*. 2021;16(4):e0249572. doi:10.1371/journal.pone.0249572

32. Jarvis D, Chinn S, Luczynska C, Burney P. The association of family size with atopy and atopic disease. *Clin Exp Allergy*. Mar 1997;27(3):240-5.

33. Kansen HM, Lebbink MA, Mul J, et al. Risk factors for atopic diseases and recurrent respiratory tract infections in children. *Pediatr Pulmonol*. Nov 2020;55(11):3168-3179. doi:10.1002/ppul.25042

34. Karmaus W, Arshad H, Mattes J. Does the sibling effect have its origin in utero? Investigating birth order, cord blood immunoglobulin E concentration, and allergic sensitization at age 4 years. *Am J Epidemiol*. Nov 15 2001;154(10):909-15. doi:10.1093/aje/154.10.909

35. Kerkhof M, Koopman LP, van Strien RT, et al. Risk factors for atopic dermatitis in infants at high risk of allergy: the PIAMA study. *Clin Exp Allergy*. Oct 2003;33(10):1336-41. doi:10.1046/j.1365-2222.2003.01751.x

36. Kerkhof M, Wijga A, Smit HA, et al. The effect of prenatal exposure on total IgE at birth and sensitization at twelve months and four years of age: The PIAMA study. *Pediatr Allergy Immunol*. Feb 2005;16(1):10-8. doi:10.1111/j.1399-3038.2005.00217.x

37. Kikkawa T, Yorifuji T, Fujii Y, et al. Birth order and paediatric allergic disease: A nationwide longitudinal survey. *Clin Exp Allergy*. May 2018;48(5):577-585. doi:10.1111/cea.13100

38. Kim H, Jang E, Jung Y, et al. The Effect of Family History of Allergy on the Development of Atopic Dermatitis in Infancy. *Allergy Asthma & Respiratory Diseases*. 2009 2009;19(2):106-114.

39. Kinra S, Davey Smith G, Jeffreys M, Gunnell D, Galobardes B, McCarron P. Association between sibship size and allergic diseases in the Glasgow Alumni Study. *Thorax*. Jan 2006;61(1):48-53. doi:10.1136/thx.2004.034595

40. Koplin JJ, Dharmage SC, Ponsonby AL, et al. Environmental and demographic risk factors for egg allergy in a population-based study of infants. *Allergy*. Nov 2012;67(11):1415-22. doi:10.1111/all.12015

41. Kramer MS, Matush L, Bogdanovich N, Dahhou M, Platt RW, Mazer B. The low prevalence of allergic disease in Eastern Europe: are risk factors consistent with the hygiene hypothesis? *Clin Exp Allergy*. May 2009;39(5):708-16. doi:10.1111/j.1365-2222.2009.03205.x

42. Kusunoki T, Mukaida K, Morimoto T, et al. Birth order effect on childhood food allergy. *Pediatr Allergy Immunol*. May 2012;23(3):250-4. doi:10.1111/j.1399-3038.2011.01246.x

43. Kwon IH, Won CH, Lee DH, et al. The Prevalence and Risk Factors of Atopic Dermatitis and Clinical Characteristics according to Disease Onset in 19-Year-Old Korean Male Subjects. *Ann Dermatol*. Feb 2018;30(1):20-28. doi:10.5021/ad.2018.30.1.20

44. Larsson M, Hägerhed-Engman L, Sigsgaard T, Janson S, Sundell J, Bornehag CG. Incidence rates of asthma, rhinitis and eczema symptoms and influential factors in young children in Sweden. *Acta Paediatr*. Sep 2008;97(9):1210-5. doi:10.1111/j.1651-2227.2008.00910.x

45. Lee SL, Wong W, Lau YL. Increasing prevalence of allergic rhinitis but not asthma among children in Hong Kong from 1995 to 2001 (Phase 3 International Study of Asthma and Allergies in Childhood). *Pediatr Allergy Immunol*. Feb 2004;15(1):72-8. doi:10.1046/j.0905-6157.2003.00109.x

46. Lee SY, Kwon JW, Seo JH, et al. Prevalence of atopy and allergic diseases in Korean children: associations with a farming environment and rural lifestyle. *Int Arch Allergy Immunol*. 2012;158(2):168-74. doi:10.1159/000330820

47. Lewis SA, Britton JR. Consistent effects of high socioeconomic status and low birth order, and the modifying effect of maternal smoking on the risk of allergic disease during childhood. *Respir Med*. Oct 1998;92(10):1237-44. doi:10.1016/s0954-6111(98)90427-9

48. Lin C-H, Wang J-L, Chen H-H, Hsu J-Y, Chao W-C. Shared prenatal impacts among childhood asthma, allergic rhinitis and atopic dermatitis: a population-based study. *Allergy, Asthma & Clinical Immunology*. 2019/09/03 2019;15(1):52. doi:10.1186/s13223-019-0365-y

49. Linneberg A, Nielsen NH, Madsen F, Frølund L, Dirksen A, Jørgensen T. Factors related to allergic sensitization to aeroallergens in a cross-sectional study in adults: The Copenhagen Allergy Study. *Clin Exp Allergy*. Sep 2001;31(9):1409-17. doi:10.1046/j.1365-2222.2001.01178.x

50. Linneberg A, Simonsen JB, Petersen J, Stensballe LG, Benn CS. Differential effects of risk factors on infant wheeze and atopic dermatitis emphasize a different etiology. *J Allergy Clin Immunol*. Jan 2006;117(1):184-9. doi:10.1016/j.jaci.2005.09.042

51. Loo EX, Shek LP, Goh A, et al. Atopic Dermatitis in Early Life: Evidence for at Least Three Phenotypes? Results from the GUSTO Study. *Int Arch Allergy Immunol*. 2015;166(4):273-9. doi:10.1159/000381342

52. Lukkarinen M, Puosi E, Kataja EL, et al. Maternal psychological distress during gestation is associated with infant food allergy. *Pediatr Allergy Immunol*. May 2021;32(4):787-792. doi:10.1111/pai.13449

53. Lyons SA, Knulst AC, Burney PGJ, et al. Predictors of Food Sensitization in Children and Adults Across Europe. *J Allergy Clin Immunol Pract*. Oct 2020;8(9):3074-3083.e32. doi:10.1016/j.jaip.2020.04.040

54. Matricardi PM, Franzinelli F, Franco A, et al. Sibship size, birth order, and atopy in 11,371 Italian young men. *J Allergy Clin Immunol*. Apr 1998;101(4 Pt 1):439-44. doi:10.1016/s0091-6749(98)70350-1

55. McKeever TM, Lewis SA, Smith C, et al. Siblings, multiple births, and the incidence of allergic disease: a birth cohort study using the West Midlands general practice research database. *Thorax*. Oct 2001;56(10):758-62. doi:10.1136/thorax.56.10.758

56. Metsälä J, Lundqvist A, Kaila M, Gissler M, Klaukka T, Virtanen SM. Maternal and perinatal characteristics and the risk of cow's milk allergy in infants up to 2 years of age: a case-control study nested in the Finnish population. *Am J Epidemiol*. Jun 15 2010;171(12):1310-6. doi:10.1093/aje/kwq074

57. Miyake Y, Yura A, Iki M. Cross-sectional study of allergic disorders in relation to familial factors in Japanese adolescents. *Acta Paediatr*. Mar 2004;93(3):380-5. doi:10.1080/08035250410022819

58. Miyake Y, Tanaka K, Arakawa M. Sibling number and prevalence of allergic disorders in pregnant Japanese women: baseline data from the Kyushu Okinawa Maternal and Child Health Study. *BMC Public Health*. 2011/07/14 2011;11(1):561. doi:10.1186/1471-2458-11-561

59. Mommers M, Weishoff-Houben M, Swaen GM, et al. Infant immunization and the occurrence of atopic disease in Dutch and German children: a nested case-control study. *Pediatr Pulmonol*. Oct 2004;38(4):329-34. doi:10.1002/ppul.20089

60. Moncayo AL, Vaca M, Oviedo G, et al. Risk factors for atopic and non-atopic asthma in a rural area of Ecuador. *Thorax*. May 2010;65(5):409-16. doi:10.1136/thx.2009.126490

61. Musgrove K, Morgan JK. Infantile eczema: A long-term follow-up study. *Br J Dermatol*. Oct 1976;95(4):365-72. doi:10.1111/j.1365-2133.1976.tb00837.x

62. Nakamura Y, Oki I, Tanihara S, et al. Relationship between breast milk feeding and atopic dermatitis in children. *J Epidemiol*. Mar 2000;10(2):74-8. doi:10.2188/jea.10.74

63. Newson RB, van Ree R, Forsberg B, et al. Geographical variation in the prevalence of sensitization to common aeroallergens in adults: the GA(2) LEN survey. *Allergy*. May 2014;69(5):643-51. doi:10.1111/all.12397

64. Nicolaou NC, Simpson A, Lowe LA, Murray CS, Woodcock A, Custovic A. Day-care attendance, position in sibship, and early childhood wheezing: a population-based birth cohort study. *J Allergy Clin Immunol*. Sep 2008;122(3):500-6.e5. doi:10.1016/j.jaci.2008.06.033

65. Nowak D, Heinrich J, Jörres R, et al. Prevalence of respiratory symptoms, bronchial hyperresponsiveness and atopy among adults: west and east Germany. *Eur Respir J*. Dec 1996;9(12):2541-52. doi:10.1183/09031936.96.09122541

66. Nystad W, Skrondal A, Njå F, Hetlevik O, Carlsen KH, Magnus P. Recurrent respiratory tract infections during the first 3 years of life and atopy at school age. *Allergy*. Dec 1998;53(12):1189-94. doi:10.1111/j.1398-9995.1998.tb03840.x

67. Ogbuanu IU, Karmaus WJ, Zhang H, et al. Birth order modifies the effect of IL13 gene polymorphisms on serum IgE at age 10 and skin prick test at ages 4, 10 and 18: a prospective birth cohort study. *Allergy Asthma Clin Immunol*. Apr 20 2010;6(1):6. doi:10.1186/1710-1492-6-6

68. Ohfuji S, Miyake Y, Arakawa M, Tanaka K, Sasaki S. Sibship size and prevalence of allergic disorders in Japan: the Ryukyus Child Health Study. *Pediatr Allergy Immunol*. Jun 2009;20(4):377-84. doi:10.1111/j.1399-3038.2008.00804.x

69. Olesen AB, Ellingsen AR, Olesen H, Juul S, Thestrup-Pedersen K. Atopic dermatitis and birth factors: historical follow up by record linkage. *Bmj*. Apr 5 1997;314(7086):1003-8. doi:10.1136/bmj.314.7086.1003

70. Ozmert EN, Kale-Cekinmez E, Yurdakök K, Sekerel BE. Determinants of allergic signs and symptoms in 24- 48-month-old Turkish children. *Turk J Pediatr*. Mar-Apr 2009;51(2):103-9.

71. Palacios-Lopez CG, Orozco-Covarrubias L, Tamayo-Sánchez L, Duran-McKinster C, Ruiz-Maldonado R. Atopic dermatitis: increased prevalence and the influence of birth, siblings and maternal factors. *Acta Derm Venereol*. May 2001;81(2):145-6. doi:10.1080/00015550152384353

72. Parthasarathi A, Padukudru S, Rajgopal N, Holla AD, Krishna MT, Mahesh PA. Allergic disease prevalence in school children in Bengaluru, India: A cross-sectional survey. *Clin Exp Allergy*. Jul 2021;51(7):955-958. doi:10.1111/cea.13881

73. Pekkanen J, Remes S, Kajosaari M, Husman T, Soininen L. Infections in early childhood and risk of atopic disease. *Acta Paediatr*. Jul 1999;88(7):710-4. doi:10.1080/08035259950168964

74. Pekkanen J, Xu B, Järvelin MR. Gestational age and occurrence of atopy at age 31--a prospective birth cohort study in Finland. *Clin Exp Allergy*. Jan 2001;31(1):95-102.

75. Perzanowski MS, Canfield SM, Chew GL, et al. Birth order, atopy, and symptoms of allergy and asthma among inner-city children attending Head Start in New York City. *Clin Exp Allergy*. Jun 2008;38(6):968-76. doi:10.1111/j.1365-2222.2008.02967.x

76. Peters RL, Allen KJ, Dharmage SC, et al. Differential factors associated with challenge-proven food allergy phenotypes in a population cohort of infants: a latent class analysis. *Clin Exp Allergy*. May 2015;45(5):953-963. doi:10.1111/cea.12478

77. Ponsonby AL, Couper D, Dwyer T, Carmichael A. Cross sectional study of the relation between sibling number and asthma, hay fever, and eczema. *Arch Dis Child*. Oct 1998;79(4):328-33. doi:10.1136/adc.79.4.328

78. Ponsonby AL, Dwyer T, Kemp A, Lim L, Cochrane J, Carmichael A. The use of mutually exclusive categories for atopic sensitization: a contrasting effect for family size on house dust mite sensitization compared with ryegrass sensitization. *Pediatr Allergy Immunol*. Apr 2003;14(2):81-90. doi:10.1034/j.1399-3038.2003.00023.x

79. Purvis DJ, Thompson JM, Clark PM, et al. Risk factors for atopic dermatitis in New Zealand children at 3.5 years of age. *Br J Dermatol*. Apr 2005;152(4):742-9. doi:10.1111/j.1365-2133.2005.06540.x

80. Rangkakulnuwat P, Lao-Araya M. The prevalence and temporal trends of food allergy among preschool children in Northern Thailand between 2010 and 2019. *World Allergy Organ J*. Oct 2021;14(10):100593. doi:10.1016/j.waojou.2021.100593

81. Raukas-Kivioja A, Raukas ES, Meren M, Loit HM, Rönmark E, Lundbäck B. Allergic sensitization to common airborne allergens among adults in Estonia. *Int Arch Allergy Immunol*. 2007;142(3):247-54. doi:10.1159/000097027

82. Rönmark E, Bjerg A, Perzanowski M, Platts-Mills T, Lundbäck B. Major increase in allergic sensitization in schoolchildren from 1996 to 2006 in northern Sweden. *J Allergy Clin Immunol*. Aug 2009;124(2):357-63, 63.e1-15. doi:10.1016/j.jaci.2009.05.011

83. Rönmark EP, Ekerljung L, Mincheva R, et al. Different risk factor patterns for adult asthma, rhinitis and eczema: results from West Sweden Asthma Study. *Clin Transl Allergy*. 2016;6:28. doi:10.1186/s13601-016-0112-0

84. Rönmark E, Warm K, Bjerg A, Backman H, Hedman L, Lundbäck B. High incidence and persistence of airborne allergen sensitization up to age 19 years. *Allergy*. May 2017;72(5):723-730. doi:10.1111/all.13053

85. Rutter CE, Silverwood RJ, Asher MI, et al. Comparison of individual-level and population-level risk factors for rhinoconjunctivitis, asthma, and eczema in the International Study of Asthma and Allergies in Childhood (ISAAC) Phase Three. *World Allergy Organ J*. Jun 2020;13(6):100123. doi:10.1016/j.waojou.2020.100123

86. Sardecka I, Łoś-Rycharska E, Ludwig H, Gawryjołek J, Krogulska A. Early risk factors for cow's milk allergy in children in the first year of life. *Allergy Asthma Proc*. Nov 1 2018;39(6):e44-e54. doi:10.2500/aap.2018.39.4159

87. Sasaki M, Yoshida K, Adachi Y, et al. Environmental factors associated with childhood eczema: Findings from a national web-based survey. *Allergol Int*. Oct 2016;65(4):420-424. doi:10.1016/j.alit.2016.03.007

88. Schäfer S, Liu A, Campbell D, Nanan R. Analysis of maternal and perinatal determinants of allergic sensitization in childhood. *Allergy, Asthma & Clinical Immunology*. 2020/07/31 2020;16(1):71. doi:10.1186/s13223-020-00467-5

89. Sears MR, Holdaway MD, Flannery EM, Herbison GP, Silva PA. Parental and neonatal risk factors for atopy, airway hyper-responsiveness, and asthma. *Arch Dis Child*. Nov 1996;75(5):392-8. doi:10.1136/adc.75.5.392

90. Sheldrake P, Cormack M, McGuire J. Psychosomatic illness, birth order and intellectual preference - I. Men. *J Psychosom Res*. 1976;20(1):37-44. doi:10.1016/0022-3999(76)90098-2

91. Sheldrake P, Cormack M, McGuire J. Psychosomatic illness, birth order and intellectual preference - II. Women. *J Psychosom Res*. 1976;20(1):45-9. doi:10.1016/0022-3999(76)90099-4

92. Slob EMA, Brew BK, Vijverberg SJH, et al. Early-life antibiotic use and risk of asthma and eczema: results of a discordant twin study. *Eur Respir J*. Apr 2020;55(4)doi:10.1183/13993003.02021-2019

93. Sozańska B, Pearce N, Błaszczyk M, Boznański A, Cullinan P. Changes in atopy prevalence and sibship effect in rural population at all ages. *Allergy*. Jun 2015;70(6):661-6. doi:10.1111/all.12623

94. Stemeseder T, Klinglmayr E, Moser S, et al. Influence of Intrinsic and Lifestyle Factors on the Development of IgE Sensitization. *International Archives of Allergy and Immunology*. 2017;173(2):99-104. doi:10.1159/000475499

95. Strachan DP. Hay fever, hygiene, and household size. *Bmj*. Nov 18 1989;299(6710):1259-60. doi:10.1136/bmj.299.6710.1259

96. Strachan DP, Taylor EM, Carpenter RG. Family structure, neonatal infection, and hay fever in adolescence. *Arch Dis Child*. May 1996;74(5):422-6. doi:10.1136/adc.74.5.422

97. Strachan DP, Harkins LS, Johnston ID, Anderson HR. Childhood antecedents of allergic sensitization in young British adults. *J Allergy Clin Immunol*. Jan 1997;99(1 Pt 1):6-12. doi:10.1016/s0091-6749(97)70294-x

98. Strachan DP, Aït-Khaled N, Foliaki S, et al. Siblings, asthma, rhinoconjunctivitis and eczema: a worldwide perspective from the International Study of Asthma and Allergies in Childhood. *Clin Exp Allergy*. Jan 2015;45(1):126-36. doi:10.1111/cea.12349

99. Stråvik M, Barman M, Hesselmar B, Sandin A, Wold AE, Sandberg AS. Maternal Intake of Cow's Milk during Lactation Is Associated with Lower Prevalence of Food Allergy in Offspring. *Nutrients*. Nov 28 2020;12(12)doi:10.3390/nu12123680

100. Suaini NHA, Loo EX, Peters RL, et al. Children of Asian ethnicity in Australia have higher risk of food allergy and early-onset eczema than those in Singapore. *Allergy*. Oct 2021;76(10):3171-3182. doi:10.1111/all.14823

101. Svanes C, Jarvis D, Chinn S, Burney P. Childhood environment and adult atopy: results from the European Community Respiratory Health Survey. *J Allergy Clin Immunol*. Mar 1999;103(3 Pt 1):415-20. doi:10.1016/s0091-6749(99)70465-3

102. Svanes C, Jarvis D, Chinn S, Omenaas E, Gulsvik A, Burney P. Early exposure to children in family and day care as related to adult asthma and hay fever: results from the European Community Respiratory Health Survey. *Thorax*. Nov 2002;57(11):945-50. doi:10.1136/thorax.57.11.945

103. Taylor-Robinson DC, Williams H, Pearce A, Law C, Hope S. Do early-life exposures explain why more advantaged children get eczema? Findings from the U.K. Millennium Cohort Study. *Br J Dermatol*. Mar 2016;174(3):569-78. doi:10.1111/bjd.14310

104. Torfi Y, Bitarafan N, Rajabi M. Impact of socioeconomic and environmental factors on atopic eczema and allergic rhinitis: a cross sectional study. *Excli j*. 2015;14:1040-8. doi:10.17179/excli2015-519

105. Turner SW, Palmer LJ, Gibson NA, et al. The effect of age on the relationship between birth order and immunoglobulin E sensitization. *Clin Exp Allergy*. May 2005;35(5):630-4. doi:10.1111/j.1365-2222.2005.02229.x

106. Venter C, Palumbo MP, Sauder KA, et al. Incidence and timing of offspring asthma, wheeze, allergic rhinitis, atopic dermatitis, and food allergy and association with maternal history of asthma and allergic rhinitis. *World Allergy Organ J*. Mar 2021;14(3):100526. doi:10.1016/j.waojou.2021.100526

107. Victorino CC, Gauthier AH. The social determinants of child health: variations across health outcomes - a population-based cross-sectional analysis. *BMC Pediatr*. Aug 17 2009;9:53. doi:10.1186/1471-2431-9-53

108. Von Linstow ML, Porsbjerg C, Ulrik CS, Nepper-Christensen S, Backer V. Prevalence and predictors of atopy among young Danish adults. *Clin Exp Allergy*. Apr 2002;32(4):520-5. doi:10.1046/j.0954-7894.2002.01326.x

109. von Mutius E, Martinez FD, Fritzsch C, Nicolai T, Reitmeir P, Thiemann HH. Skin test reactivity and number of siblings. *Bmj*. Mar 12 1994;308(6930):692-5. doi:10.1136/bmj.308.6930.692

110. Xu B, Järvelin MR, Pekkanen J. Prenatal factors and occurrence of rhinitis and eczema among offspring. *Allergy*. Aug 1999;54(8):829-36. doi:10.1034/j.1398-9995.1999.00117.x

111. Yamazaki S, Shima M, Nakadate T, et al. Patterns of Sensitization to Inhalant Allergens in Japanese Lower-Grade Schoolchildren and Related Factors. *Int Arch Allergy Immunol*. 2015;167(4):253-63. doi:10.1159/000439534

112. Zekveld C, Bibakis I, Bibaki-Liakou V, et al. The effects of farming and birth order on asthma and allergies. *European Respiratory Journal*. 2006;28(1):82. doi:10.1183/09031936.06.00021305

113. Ziyab AH. Prevalence of food allergy among schoolchildren in Kuwait and its association with the coexistence and severity of asthma, rhinitis, and eczema: A cross-sectional study. *World Allergy Organ J*. 2019;12(4):100024. doi:10.1016/j.waojou.2019.100024

114. Zutavern A, Hirsch T, Leupold W, Weiland S, Keil U, von Mutius E. Atopic dermatitis, extrinsic atopic dermatitis and the hygiene hypothesis: results from a cross-sectional study. *Clin Exp Allergy*. Oct 2005;35(10):1301-8. doi:10.1111/j.1365-2222.2005.02350.x
